# Integrated Pharmacokinetics, Pharmacodynamics, and Pharmacometabolomics to Elucidate Guizhi Fuling Capsule’s Homeostatic Mechanism Against Acute Dysmenorrhea

**DOI:** 10.3390/ph19091438

**Published:** 2026-09-10

**Authors:** Xin-Ru Lyu, Min Lin, Zi-Han Xu, Si-Tao Xu, Xiang Li, Zhi-Hui Lu, Tong-Tong Wei, Shi-Yu Zhang, Guang-Ji Wang, Ying Peng, Jian-Guo Sun

**Affiliations:** 1 Jiangsu Provincial Key Laboratory of Drug Metabolism and Pharmacokinetics, China Pharmaceutical University, Nanjing 210009, China; 3123074303@stu.cpu.edu.cn (X.-R.L.); 3224071792@stu.cpu.edu.cn (M.L.); 3225071719@stu.cpu.edu.cn (Z.-H.X.); 3123074310@stu.cpu.edu.cn (S.-T.X.); 3324071899@stu.cpu.edu.cn (X.L.); 3225071715@stu.cpu.edu.cn (Z.-H.L.); 3325071807@stu.cpu.edu.cn (T.-T.W.); 3325071721@stu.cpu.edu.cn (S.-Y.Z.); gjwang@cpu.edu.cn (G.-J.W.); 2 State Key Laboratory of Natural Medicines, China Pharmaceutical University, Nanjing 210009, China; 3 School of Basic Medicine and Clinical Pharmacy, China Pharmaceutical University, Nanjing 211198, China

**Keywords:** Guizhi Fuling capsule, homeostasis, pharmacometabolomics, primary dysmenorrhea, Ptgs2, traditional Chinese medicine

## Abstract

**Background/Objectives:** Guizhi Fuling Capsule (GZFL), a Traditional Chinese Medicine (TCM) formula, is widely used for primary dysmenorrhea and other blood-stasis gynecological disorders. This study aimed to characterize its material basis, elucidate its multi-component, multi-target mechanism against acute primary dysmenorrhea, and establish an integrated pharmacokinetic-pharmacometabolomic-pharmacodynamic (PK-PM-PD) framework for TCM efficacy evaluation. **Methods:** GZFL constituents and serum metabolites in an oxytocin-/estradiol-induced rat dysmenorrhea model were characterized by UPLC/Q-TOF-MS. Uterine effects of GZFL-containing serum were assessed ex vivo. The active components of GZFL were screened by Chinmedomics, with candidate targets investigated through network pharmacology, transcriptomics, and molecular docking. In total, 23 pharmacodynamic indicators were integrated by principal component analysis into an Efficacy Index (EI). Correlation analysis between pharmacometabolomic and pharmacodynamic data yielded a Metabolite-Efficacy Index (MEI), evaluated across a 21-day time course. **Results:** Among 197 constituents characterized in GZFL extract, 136 serum-exposed components were detected, with several key metabolites enriched via biotransformation. GZFL-containing serum bidirectionally regulated uterine contractility toward the control level. Integrated analyses revealed 68 candidate therapeutic targets. GZFL suppressed NF-κB/IKKβ signaling, down-regulated COX-2/iNOS, restored the PGF2α/PGE2 balance, and normalized inflammatory cytokines. Eleven efficacy-associated metabolites correlated with pharmacodynamic recovery were revealed and integrated, with MEI achieving the highest predictive performance among five integration strategies (AUC = 0.9) and robustly tracking the full 21-day disease-recovery trajectory. **Conclusions:** GZFL attenuates dysmenorrhea through coordinated regulation of inflammation, prostaglandin metabolism, and uterine functional homeostasis, rather than through inhibition of a single target. The PK-PM-PD framework, with EI and MEI, offers a reproducible paradigm for evaluating complex TCM therapies.

## 1. Introduction

Guizhi Fuling (GZFL) prescription, a classical traditional Chinese medicine (TCM) formula first documented in the *Synopsis of the Golden Chamber* by Zhang Zhongjing during the Eastern Han dynasty, has been employed for over two millennia in the treatment of gynecological disorders characterized by blood stasis, postpartum lochiorrhea, amenorrhea, and menstrual pain [1]. The formula is composed of five medicinal herbs: *Ramulus Cinnamomi*, *Poria, Cortex Moutan*, *Semen Persicae*, and *Radix Paeoniae Alba* [2]. Building upon this classical foundation, the modern GZFL capsule was developed through contemporary pharmaceutical technology, and is now widely prescribed for primary dysmenorrhea, uterine fibroids, pelvic inflammatory disease, and endometriosis, demonstrating notable clinical efficacy with a favourable safety profile [3].

Modern pharmacological investigations have demonstrated that the therapeutic effects of GZFL are associated with multiple bioactive constituents derived from its component herbs [4,5]. Paeoniflorin, a characteristic monoterpene glycoside from *Radix Paeoniae Alba* and *Cortex Moutan*, has been reported to exhibit anti-inflammatory, analgesic, and immunomodulatory activities [6]. Paeonol, a major phenolic constituent of *Cortex Moutan*, possesses antioxidative and inflammation-regulating properties [7]. Cinnamic acid and amygdalin, originating from *Ramulus Cinnamomi* and *Semen Persicae*, respectively, have also been implicated in vascular regulation, inflammatory modulation, and pain-related responses [8,9]. However, the contributions of these compounds and their circulating metabolites to the overall therapeutic effects of GZFL remain incompletely understood.

Primary dysmenorrhea is among the most prevalent gynecological conditions, substantially affecting quality of life due to its marked periodicity and multifactorial etiology. Epidemiological studies report a prevalence of 45–95% among women of reproductive age, with an apparently increasing trend in recent years [10]. In the absence of underlying organic pathology, disruption of endocrine balance elevates the ratio of prostaglandin F2α (PGF2α) to prostaglandin E2 (PGE2), promoting excessive uterine smooth muscle contraction and cyclic menstrual pain [11]. As early as the 1980s, Finn proposed that menstruation could be conceptualized as an inflammatory process [12,13], a view now supported by extensive evidence implicating prostaglandins, cytokines, and the NF-κB signalling axis in dysmenorrhea pathogenesis.

As a first-line pharmacological treatment for menstrual pain [14], nonsteroidal anti-inflammatory drugs (NSAIDs) act primarily by inhibiting cyclooxygenase (COX-1/2), thereby reducing prostaglandin synthesis and alleviating excessive uterine contractions. However, approximately 18% of women with dysmenorrhea are unresponsive to NSAID therapy, potentially owing to factors such as poor adherence, suboptimal drug exposure, and prostaglandin-independent mechanisms [15]. Emerging evidence suggests that COX-2 functions as a double-edged sword in chronic inflammation: while its early up-regulation drives the initiation of the inflammatory response, elevated COX-2 expression is also required for the resolution phase, a process that is abrogated by selective COX-2 inhibitors such as NS-398 [16,17]. These findings suggest that indiscriminate COX inhibition may provide symptomatic relief while potentially interfering with endogenous inflammatory resolution processes and may not fully address the complex pathophysiology underlying dysmenorrhea. Therapeutic strategies capable of restoring prostaglandin balance rather than simply blocking prostaglandin synthesis may therefore represent a more physiological approach.

In contrast to the single-target approach of NSAIDs, TCM advocates for simultaneously treating both the symptomatic manifestation and the underlying root cause of disease. In classical TCM theory, dysmenorrhea arises from obstruction of Qi and blood (“*bu tong ze tong*”, pain from obstruction) and malnourishment of the uterine collaterals (“*bu rong ze tong*”, pain from deficiency) [18]. The *Jingyue Quanshu* characterised the pathogenesis of primary dysmenorrhea as a condition of combined deficiency and excess, while the *Yi Zong Jinjian* advocated syndrome-differentiation-based treatment tailored to each patient’s constitution. These classical principles are conceptually consistent with the multi-target, homeostatic effects attributed to GZFL. Rather than suppressing a single enzyme, the formula is proposed to regulate blood circulation, resolve stasis, and restore the balance of the uterine microenvironment. GZFL’s capacity to exert bidirectional regulatory effects on uterine smooth muscle contractility is consistent with this classical rationale and distinguishes it mechanistically from NSAIDs.

The concept of “preventive treatment of disease” (*zhi wei bing*) [19], which is consistent with the emphasis on disease prevention and TCM modernization in China’s 15th Five-Year Plan, originates from the *Yellow Emperor’s Internal Canon of Medicine* [20], and emphasizes recognition of disease trajectories and intervention before pathological states become fully manifested. The pre-disease state (*wei bing*) encompasses subtle and dynamic changes preceding overt disease [21], highlighting the importance of monitoring disease progression rather than relying solely on symptomatic endpoints. This concept is particularly relevant to primary dysmenorrhea, a highly periodic condition in which pathological changes and clinical symptoms recur across menstrual cycles. In 2023, the China Association of Chinese Medicine promulgated the Guidelines for Preventive Treatment of Diseases in Female Physiological Cycle Recuperation, further supporting the application of preventive TCM strategies to menstrual disorders. However, given the etiological complexity and phenotypic heterogeneity of dysmenorrhea, standardized efficacy endpoints capable of capturing the holistic and dynamic therapeutic response to TCM interventions remain lacking.

The conceptual framework of Chinese medicine metabolomics (Chinmedomics) was first introduced by Wang Xijun in 2011 as an integrative discipline grounded in metabolomics methodology. Within this framework, disease-specific biomarkers are identified through comprehensive metabolic profiling, while the bioactive constituents corresponding to a given TCM syndrome are characterized via serum pharmacochemistry. Exogenous TCM components detected in serum are then correlated with endogenous pharmacodynamic markers, and those showing high correlation are postulated to constitute the material basis of the TCM formulation’s pharmacological effects [22]. This paradigm offers a rigorous and reproducible approach for elucidating both the therapeutic efficacy and the mechanistic underpinnings of TCM interventions [23]. Applied to GZFL, this approach permits simultaneous elucidation of the absorbed material basis and its dynamic interaction with the host’s endogenous metabolic network. Pharmacometabolomics can further link drug exposure to endogenous metabolic responses, whereas pharmacodynamics reflects the integrated biological outcome. Therefore, integrating these dimensions within a PK-PM-PD framework may provide a more comprehensive strategy for deciphering how multi-component herbal medicines regulate disease-associated biological networks.

Previous studies have confirmed GZFL’s therapeutic efficacy in cold coagulation dysmenorrhea rat models, primarily through modulation of COX-2 expression [24,25]. Pharmacokinetic investigations [26] identified absorbed GZFL components acting on the PI3K/Akt–Erk pathway. However, owing to GZFL’s multi-component, multi-target pharmacological profile, the precise material basis responsible for its therapeutic effects remains incompletely characterized. Critically, no prior study has established an objective, integrated framework for evaluating GZFL’s holistic therapeutic response in a manner that captures the full disease-recovery trajectory.

Based on the existing literature and our prior investigations, the present study first aimed to systematically characterize the material basis of GZFL and its in vivo metabolites using UPLC/Q-TOF-MS. We then evaluated the multi-component, multi-target mechanism of GZFL against acute primary dysmenorrhea in a rat model at behavioural, biochemical, transcriptomic, and metabolomic levels. Finally, an integrated pharmacokinetic–pharmacometabolomic–pharmacodynamic (PK-PM-PD) analytical framework was established for objectively evaluating TCM therapeutic efficacy, incorporating a composite Efficacy Index (EI) and a Metabolite–Efficacy Index (MEI). This work advances the mechanistic understanding of the material basis of a clinically important classical Chinese formula and provides a framework with potential applicability to the integrated evaluation of other complex TCM therapies.

## 2. Results

### 2.1. Chemical Characterization and Serum Pharmacochemistry of GZFL

A “diagnostic ion” bridge strategy was employed in conjunction with UPLC/Q-TOF-MS technology to systematically characterize the complex chemical constituents of GZFL and GZFL-related constituents in serum. Qualitative analysis of the GZFL extract yielded a total of 197 chemical components (Figure S1a, Table S1): 151 in negative ion mode and 46 in positive ion mode. These components were distributed across the five constituent herbs as follows: 51 from *Ramulus Cinnamomi*, 32 from *Poria*, 134 from *Radix Paeoniae Alba*, 59 from *Cortex Moutan*, and 17 from *Semen Persicae*, with 50 components shared across multiple herbs (Table 1).

Using the extract-derived compound library as a reference, chemical components were subsequently characterized in drug-containing rat serum. A total of 136 components were detected across both ionization modes (Figure S1b, Table S1): 111 in negative ion mode and 25 in positive ion mode. The herb-specific distributions were as follows: 40 from *Ramulus Cinnamomi*, 19 from *Poria*, 71 from *Radix Paeoniae Alba*, 37 from *Cortex Moutan*, and 15 from *Semen Persicae*, with 37 components shared among multiple herbs. Among the 136 serum-absorbed components, terpenoid glycosides (31, 22.8%) and terpenoids (25, 18.4%) were the predominant chemical classes (Table 2).

### 2.2. Component Abundance Analysis in GZFL Extract and Rat Serum

Serum components were ranked by relative abundance based on peak area. The 10 most abundant serum-absorbed components were: paeonoside, cinnamic acid, salicylpaeoniflorin, paeonol, prunasin, paeoniflorin, prupersin E, 3-hydroxypaeonol, dihydrocinnacasside, and poricoic acid F (Table 3). According to the Chinese Pharmacopoeia, the established quality markers (Q-markers) of GZFL include paeonol, paeoniflorin, amygdalin, and cinnamic acid. Among the serum-absorbed components, several metabolites derived from these Q-markers were identified, including paeonoside and 3-hydroxypaeonol derived from paeonol, salicylpaeoniflorin from paeoniflorin, prunasin from amygdalin, and dihydrocinnacasside from cinnamic acid.

**Figure 1:**
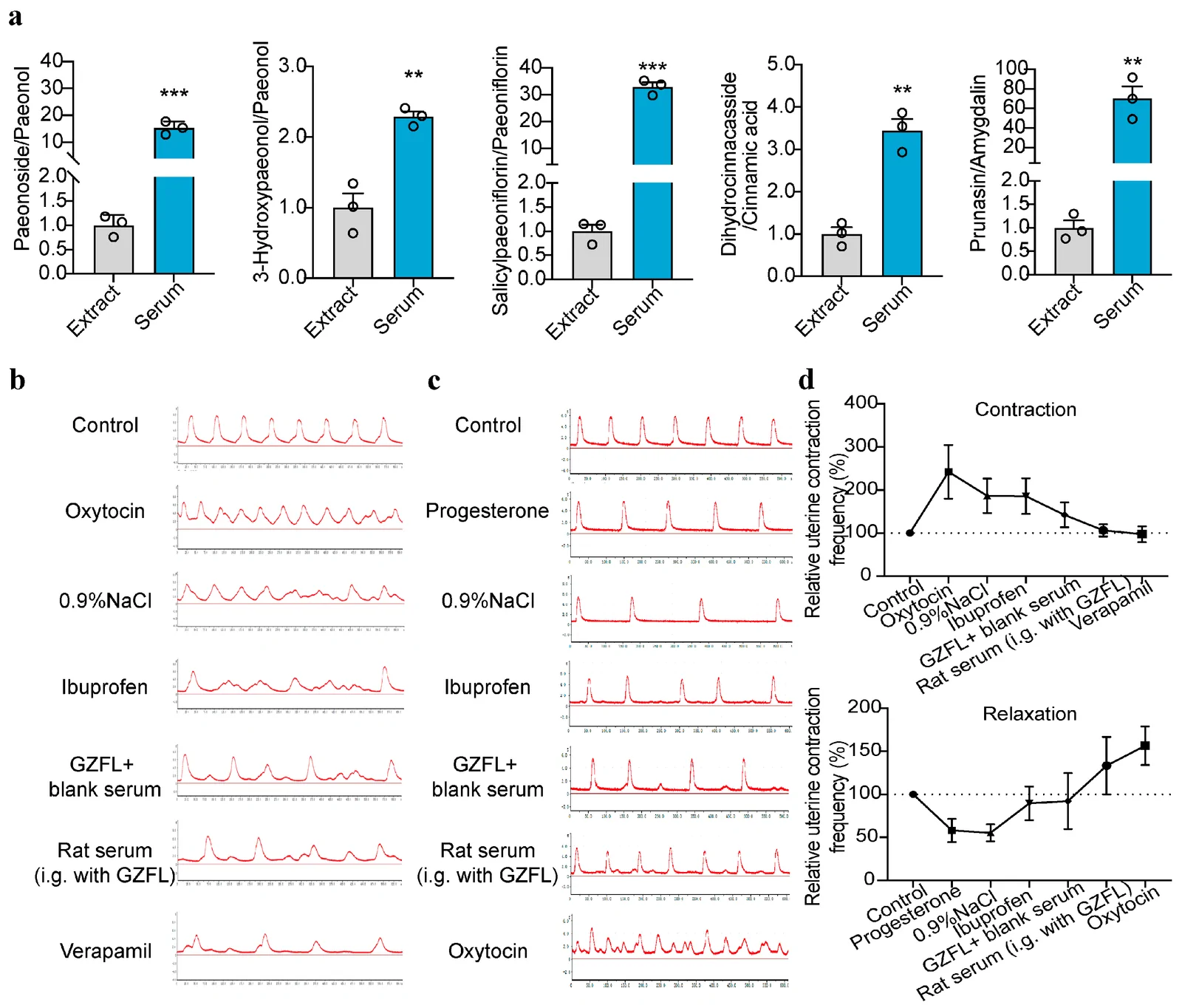
Characterization of representative serum-exposed constituents and ex vivo pharmacological effects of GZFL. (**a**). Metabolite-to-prototype ratios of representative constituents in GZFL extract and drug-containing rat serum. (**b**). Representative uterine contraction traces showing the effects of GZFL extract and drug-containing serum on oxytocin-induced uterine hypercontraction. (**c**). Representative uterine contraction traces showing the effects of GZFL extract and drug-containing serum on progesterone-induced uterine hypocontraction. (**d**). Quantitative analysis of relative uterine contractile rhythm following the indicated treatments. Data are presented as the mean ± SEM (*n* = 3). Compared with the control group: ** *p* < 0.01, and *** *p* < 0.001. Abbreviations: GZFL, Guizhi Fuling capsule; i.g., intragastric administration; NaCl, sodium chloride.

To compare the relative distribution of these metabolites and their corresponding prototype compounds between the extract and systemic circulation, the metabolite-to-prototype peak area ratios were calculated in both the GZFL extract and drug-containing serum. Compared with the GZFL extract, the metabolite-to-prototype peak area ratios were significantly elevated in rat serum following oral administration, indicating substantial changes in the relative abundance of these metabolites after systemic exposure (Figure 1a).

### 2.3. Bidirectional Regulation of Isolated Rat Uterine Contractility by GZFL

Neither GZFL extract-supplemented blank serum nor GZFL-containing serum altered the spontaneous contractile rhythm of the normal rat uterus (Figure S1c). Oxytocin and progesterone were subsequently used to induce models of abnormal uterine hypercontraction and hypocontraction, respectively. In the oxytocin-induced hypercontraction model, both ibuprofen and blank serum supplemented with GZFL extract attenuated the increased contraction frequency, whereas GZFL-containing serum tended to restore contraction frequency closer to the control level (Figure 1b,d). In the progesterone-induced hypocontraction model, ibuprofen and GZFL extract-supplemented blank serum partially restored the reduced contraction frequency, whereas GZFL-containing serum similarly tended to restore contraction frequency closer to the control level (Figure 1c,d). Collectively, these findings support a bidirectional regulatory effect of GZFL on abnormal uterine contractility and suggest that circulating GZFL-related constituents may contribute to its pharmacological activity.

### 2.4. Tentative Identification of Serum Metabolites and Network Pharmacological Analysis

**Figure 2:**
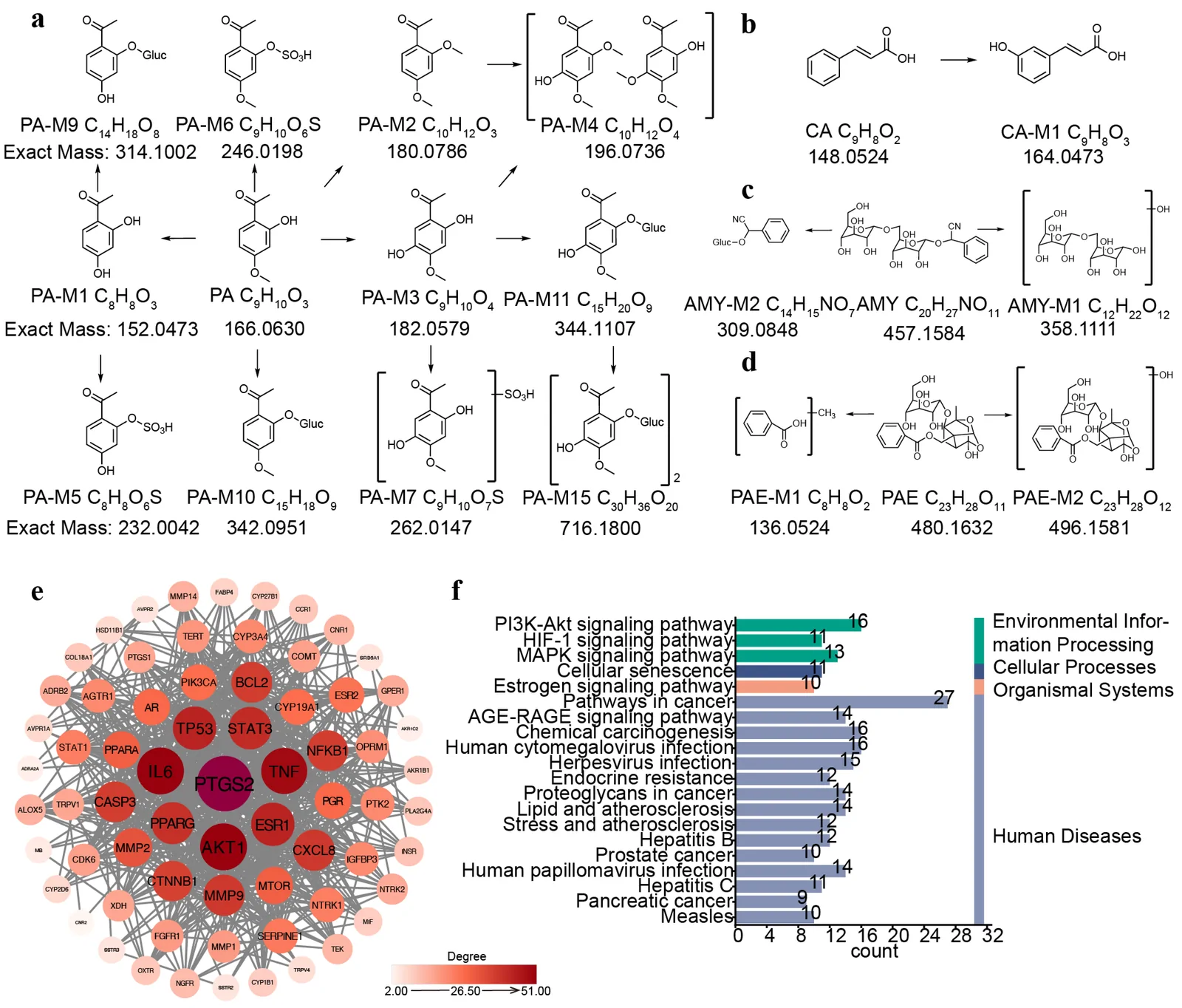
In vivo metabolite characterization and network pharmacology analysis of GZFL. (**a**). Proposed metabolic pathways of paeonol in drug-containing rat serum. (**b**). Proposed metabolic pathway of cinnamic acid in drug-containing rat serum. (**c**). Proposed metabolic pathways of amygdalin in drug-containing rat serum. (**d**). Proposed metabolic pathways of paeoniflorin in drug-containing rat serum. (**e**). PPI network of 68 candidate target proteins. (**f**). KEGG pathway enrichment analysis of the 68 candidate target proteins. Abbreviations: GZFL, Guizhi Fuling capsule; PPI, protein–protein interaction; KEGG, Kyoto Encyclopedia of Genes and Genomes.

The serum metabolic profiles of the four Q-markers of GZFL, namely paeonol, paeoniflorin, amygdalin, and cinnamic acid, were characterized in drug-containing rat serum, yielding a total of 16 metabolites. Specifically, 11 metabolites of paeonol were detected (Figure 2a), whereas one metabolite of cinnamic acid (Figure 2b), two metabolites of amygdalin (Figure 2c), and two metabolites of paeoniflorin (Figure 2d) were characterized. Complementary studies in primary human hepatocytes revealed additional metabolic profiles of paeonol, paeoniflorin, and amygdalin. Based on accurate-mass and MS/MS fragmentation data, the detected metabolites were tentatively assigned as follows: paeonol was partially metabolized via demethylation, mono-oxidation, and glucuronidation to produce three metabolites. Paeoniflorin was extensively metabolized through deglycosylation, cleavage, ring opening, and glycine conjugation to yield two metabolites. Amygdalin underwent partial metabolism via cleavage of the cyanogenic moiety and oxidation to produce one metabolite (Table 4, Figures S2–S4).

Target prediction was subsequently performed for all 136 characterized components, yielding 690 putative therapeutic targets. Multi-keyword database searches retrieved 4569 targets associated with uterine contraction, 3292 with uterine relaxation, and 560 with primary dysmenorrhea. Intersection analysis of these disease-associated target sets and the predicted compound targets revealed 68 candidate therapeutic targets (Figure S1d). KEGG (Figure 2f) and GO (Figure S1e) enrichment analyses of these 68 candidate targets revealed that GZFL primarily modulates cellular responses to hormones, nitrogen-containing compounds, and steroids, as well as processes of cell motility, proliferation, and hemodynamic regulation in the context of primary dysmenorrhea. The implicated biological processes involve the activation of nuclear receptors, oxidoreductases, transcription factors, and estrogen-responsive proteins, with downstream regulation of the PI3K–Akt, MAPK, and estrogen signaling pathways, along with modulation of cellular senescence and endocrine homeostasis.

Protein–protein interaction (PPI) network analysis demonstrated that the 68 candidate targets formed a network comprising 689 edges. Ranking the targets by degree centrality revealed PTGS2, TP53, STAT3, IL-6, TNF, PPARG, ESR1, AKT1, CXCL8, and MMP9 as potential core targets underlying the anti-dysmenorrhea effects of GZFL (Figure 2e). A component–target interaction network was subsequently constructed (Figure S1f). The network comprised 117 candidate active components, including 34 from *Ramulus Cinnamomi* (22 herb-specific), 58 from *Radix Paeoniae Alba* (27 herb-specific), 14 from *Semen Persicae* (9 herb-specific), 34 from *Cortex Moutan* (8 herb-specific), and 19 from *Poria* (17 herb-specific). Additionally, 26 components were shared between *Cortex Moutan* and *Radix Paeoniae Alba*, and 10 were shared between *Ramulus Cinnamomi* and *Radix Paeoniae Alba* (Table S2). The resulting network contained 790 compound–target interactions involving the 117 candidate active components and 68 candidate targets, supporting a multi-component, multi-target pharmacological basis for the effects of GZFL in primary dysmenorrhea.

### 2.5. Therapeutic Efficacy of GZFL in Rat Models of Dysmenorrhea

**Figure 3:**
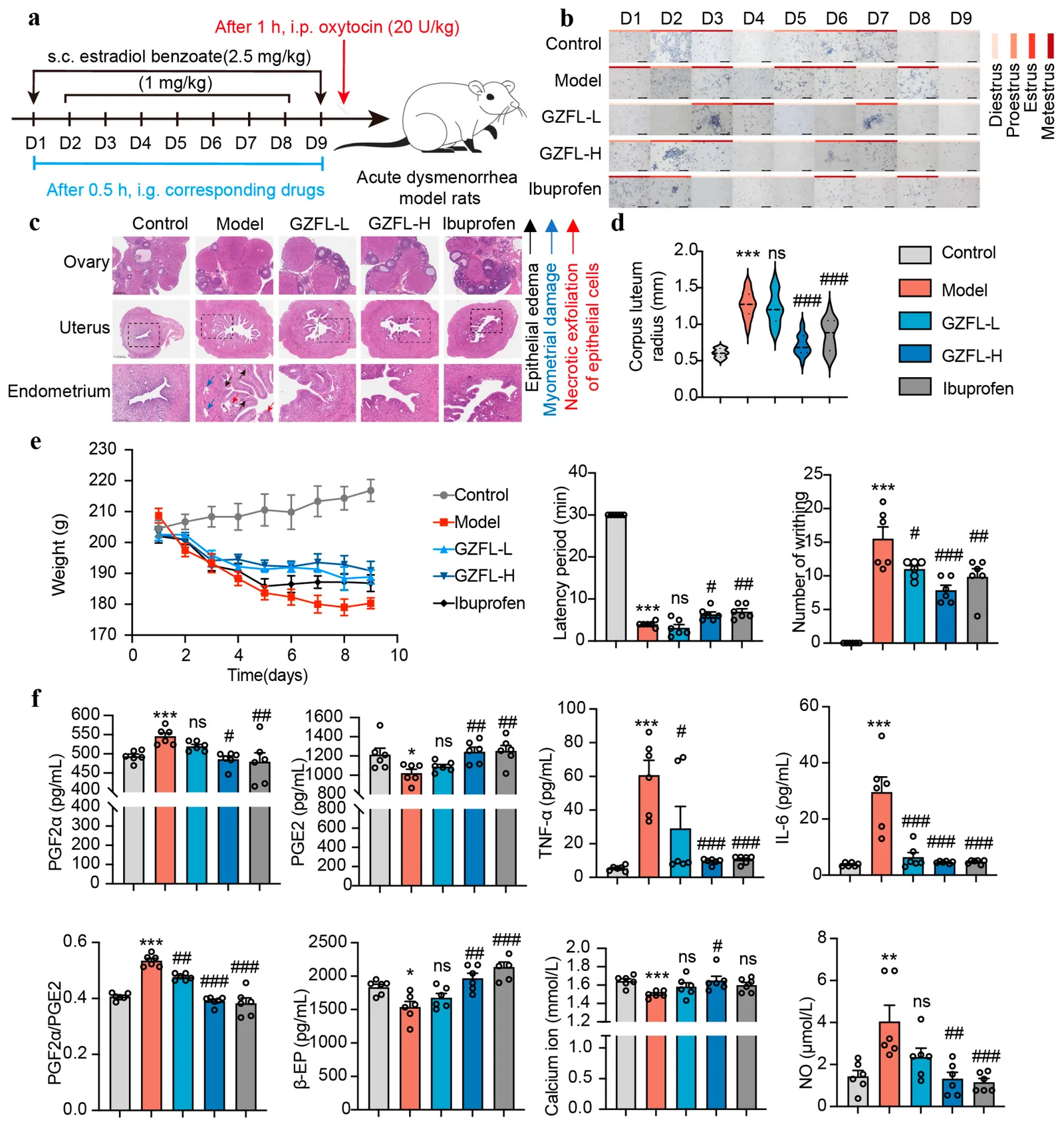
Assessment of the in vivo pharmacological efficacy of GZFL in an acute dysmenorrhea rat model. (**a**). Schematic timeline of acute dysmenorrhea model induction and drug administration. (**b**). Estrous cycle dynamics across experimental groups during model establishment (scale bar = 100 μm). (**c**). Representative histopathological sections of ovarian and uterine tissues (ovary and uterus: scale bar = 400 μm; endometrium: scale bar = 200 μm). (**d**). Quantitative analysis of corpus luteum radius. (**e**). Changes in body weight during the experimental period and behavioral assessment of dysmenorrhea, including latency period and writhing frequency. (**f**). Serum levels of PGF2α, PGE2, TNF-α, IL-6, β-EP, Ca^2+^, and NO, and the PGF2α/PGE2 ratio. Data are presented as the mean ± SEM (*n* = 6). Compared with the control group: * *p* < 0.05, ** *p* < 0.01, and *** *p* < 0.001; compared with the model group: # *p* < 0.05, ## *p* < 0.01, and ### *p* < 0.001; ns, not significant. The dotted boxes indicate the regions shown at higher magnification. Abbreviations: GZFL, Guizhi Fuling capsule; PGF2α, prostaglandin F2α; PGE2, prostaglandin E2; TNF-α, tumor necrosis factor-α; IL-6, interleukin-6; β-EP, β-endorphin; Ca^2+^, calcium ion; NO, nitric oxide; i.p., intraperitoneal injection; i.g., intragastric administration; s.c., subcutaneous injection.

A 9-day rat model of acute dysmenorrhea was established and treated with the respective interventions (Figure 3a). Daily monitoring of the estrous cycle revealed distinct patterns across groups. Rats in the control group exhibited a generally normal estrous cycle, whereas those in the model group showed disrupted cyclicity. Following treatment, estrous cyclicity was improved in the GZFL-L and GZFL-H groups, whereas disruption persisted in the ibuprofen group (Figure 3b, Table 5).

Histopathological examination revealed well-ordered follicular arrangement with minimal follicular atresia in the control group. Following model induction, follicular disorganization, increased atresia, and corpus luteum enlargement were observed. These changes were ameliorated by drug treatment, particularly in the GZFL-H and ibuprofen groups. Uterine histology in the control group showed intact endometrial epithelium with normal endometrial and myometrial morphology. Model induction resulted in disorganized uterine architecture, an irregular luminal surface, marked endometrial hyperplasia, epithelial edema, myometrial damage, and necrotic exfoliation of epithelial cells, all of which were attenuated following treatment (Figure 3c,d).

Body weights were comparable across groups on Day 0. After 9 days, body weight was decreased in all experimental groups relative to the control group. Estradiol benzoate administration increased uterine and ovarian weights compared with the control group (Figure S5a). Writhing behavior was absent in the control group, whereas model rats displayed pronounced writhing responses with markedly shortened writhing latency. GZFL-H and ibuprofen significantly reduced writhing frequency and prolonged writhing latency compared with the model group (Figure 3e). Serum PGF2α levels were significantly elevated in model rats and markedly reduced following GZFL-H treatment, whereas serum PGE2 levels were significantly decreased in the model group and increased following treatment. Consequently, the PGF2α/PGE2 ratio was significantly elevated in the model group and reduced by GZFL treatment. Serum TNF-α, IL-6, and nitric oxide (NO) levels were significantly increased in model rats and reduced following treatment. Alterations in serum β-endorphin (β-EP) and calcium levels were also ameliorated following treatment (Figure 3f).

**Figure 4:**
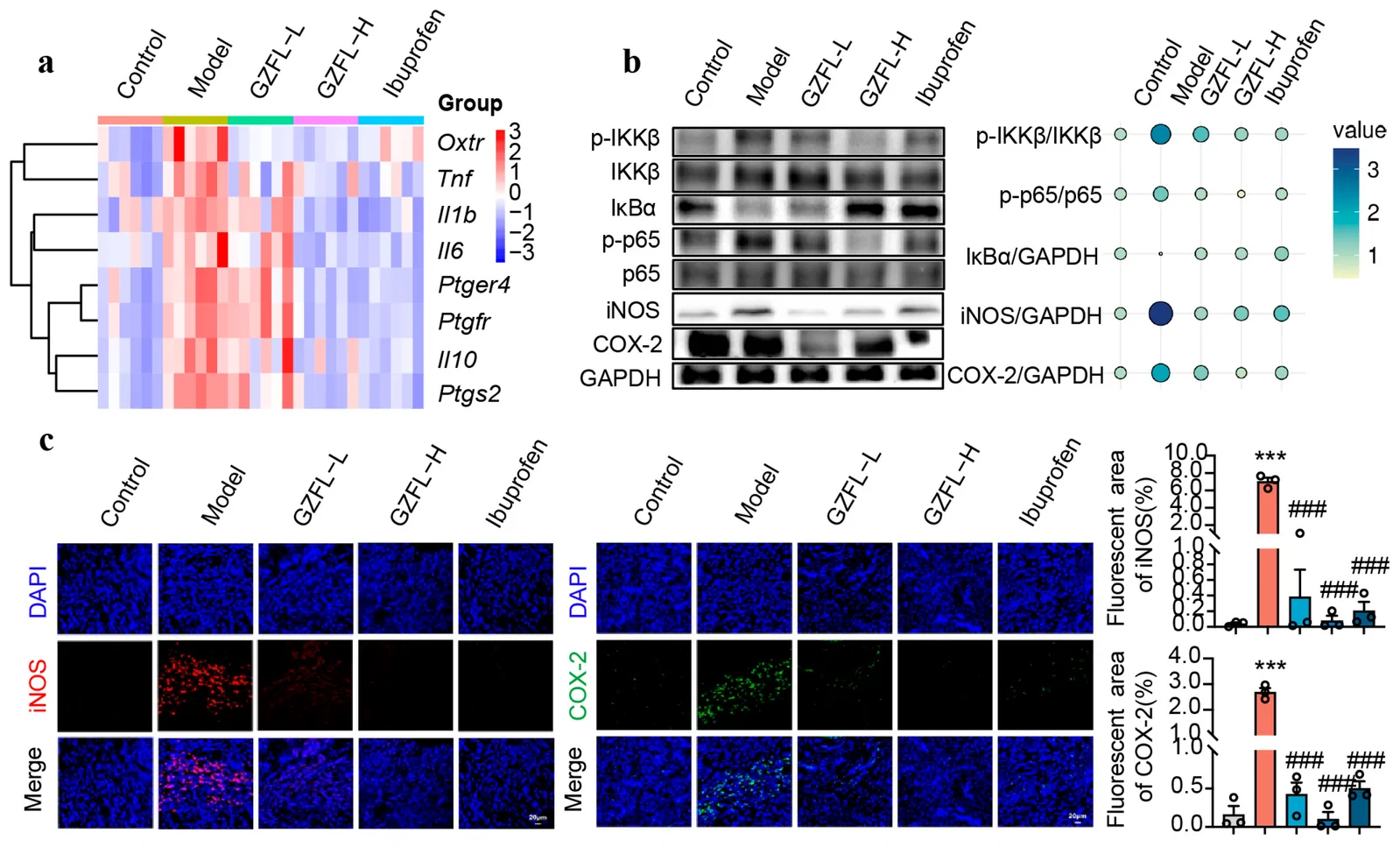
Effects of GZFL on uterine inflammatory responses in rats with acute dysmenorrhea. (**a**). Relative expression of inflammatory and uterine contraction-related genes in uterine tissue across experimental groups. (**b**). Expression and quantitative analysis of inflammation-related proteins in uterine tissue across experimental groups. (**c**). Representative immunofluorescence staining and quantitative analysis of iNOS and COX-2 expression in uterine tissue (scale bar = 20 μm). Data are presented as the mean ± SEM (*n* = 3). Compared with the control group: and *** *p* < 0.001; compared with the model group: and ### *p* < 0.001. Abbreviations: GZFL, Guizhi Fuling capsule; IKKβ, inhibitor of nuclear factor kappa-B kinase subunit beta; IκBα, inhibitor of nuclear factor kappa-B alpha; iNOS, inducible nitric oxide synthase; COX-2, cyclooxygenase-2; GAPDH, glyceraldehyde-3-phosphate dehydrogenase.

At the molecular level, GZFL and ibuprofen significantly modulated the mRNA expression of the oxytocin receptor (*Oxtr*), cyclooxygenase-2 (*Ptgs2*), prostaglandin F receptor (*Ptgfr*), prostaglandin E receptor (*Ptger4*), interleukin-10 (*Il10*), interleukin-6 (*Il6*), interleukin-1β (*Il1b*), and tumor necrosis factor (*Tnf*) (Figure 4a). Phosphorylation of IKKβ and p65 in uterine tissue was significantly elevated in model rats relative to controls and reduced following treatment with GZFL or ibuprofen. Correspondingly, IκBα expression was significantly reduced in the model group and increased following treatment. Expression of iNOS and COX-2 was markedly up-regulated in model rats and reduced following treatment (Figure 4b). Immunofluorescence staining confirmed that both GZFL and ibuprofen significantly reduced iNOS and COX-2 protein expression in uterine tissue from model rats (Figure 4c). GZFL also exhibited therapeutic effects in rats with cold coagulation and blood stasis dysmenorrhea (Figures S6 and S7).

### 2.6. Integration of Pharmacodynamic Metrics and Serum Metabolomics

**Figure 5:**
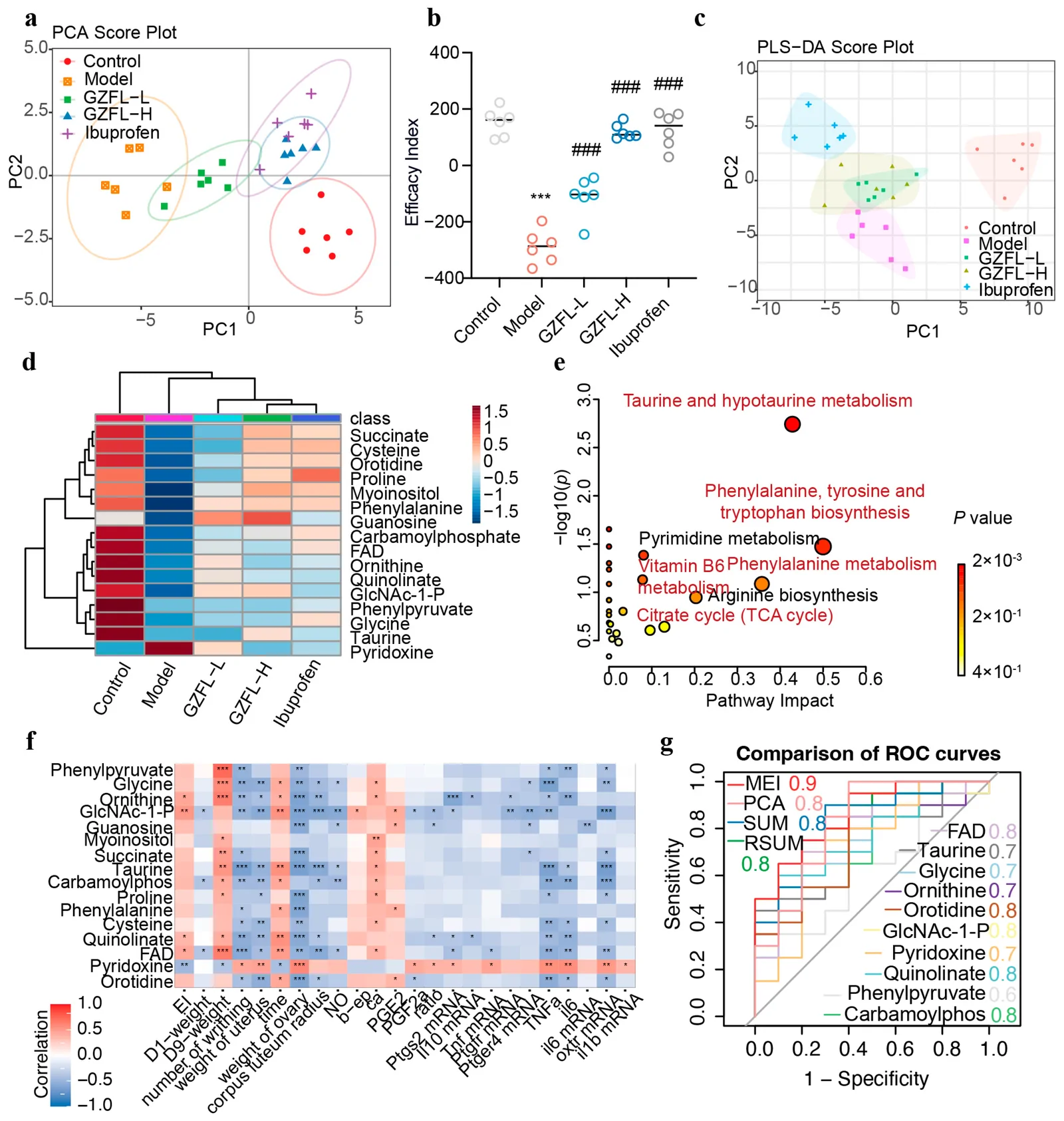
Integrated pharmacodynamic and serum metabolomics analysis of GZFL. (**a**). PCA score plot of pharmacodynamic indicators across experimental groups. (**b**). Integrated efficacy index (EI) across experimental groups. (**c**). PLS-DA score plot of serum metabolomic profiles across experimental groups. (**d**). Heatmap of differential endogenous metabolites across experimental groups. (**e**). Metabolic pathway analysis of differential endogenous metabolites. (**f**). Spearman correlation analysis between differential endogenous metabolites and pharmacodynamic indicators. (**g**). ROC curve analysis comparing different metabolite integration strategies and individual differential metabolites for evaluating therapeutic efficacy. In (**b**), *** *p* < 0.001 compared with the control group and ### *p* < 0.001 compared with the model group; in (**f**), *, **, and *** indicate correlation significance at *p* < 0.05, *p* < 0.01, and *p* < 0.001, respectively. Abbreviations: GZFL, Guizhi Fuling capsule; PCA, principal component analysis; EI, efficacy index; PLS-DA, partial least squares discriminant analysis; ROC, receiver operating characteristic; MEI, metabolite efficacy index; SUM, sum-based integration; R-SUM, rank-weighted sum; GlcNAc-1-P, N-acetylglucosamine 1-phosphate.

Principal component analysis (PCA) was performed on the 23 pharmacodynamic indicators to reduce dimensionality (Figure 5a). Five principal components were retained based on eigenvalue magnitude and a cumulative variance threshold exceeding 80% (Figure S5b, Table 6). The variance contribution rate for each principal component was calculated according to Equation (1), where $\lambda_{i}$ denotes the eigenvalue of the *i*th principal component and $\sum_{j=1}^{5}\lambda_{j}$ represents the sum of the eigenvalues of the five retained principal components.

(1) $$w_{i}=\frac{\lambda_{i}}{\sum_{j=1}^{5}\lambda_{j}},$$

Variable loadings on each principal component were computed to derive the component score coefficient matrix. Principal component scores were then calculated from the standardized pharmacodynamic data. The composite efficacy index (EI), *f*, was obtained by weighting the individual principal component scores according to their respective variance contribution rates, as defined in Equation (2). Each principal component score, *f_k_*, was calculated according to Equation (3), where $a_{kj}$ represents the loading coefficient between the *k*th principal component and the *j*th original variable, and $z_{j}$ denotes the standardized value of variable *j* (Figure 5b).

(2) $$f=w_{1}\times f_{1}+w_{2}\times f_{2}+w_{3}\times f_{3}+w_{4}\times f_{4}+w_{5}\times f_{5},$$

(3) $$f_{k}=\sum a_{kj}\times z_{j},$$

Metabolomic data were subsequently processed using validated in-house analytical methods. PCA and PLS-DA analyses showed inter-group separation and intra-group clustering. The control and model groups were well separated, while the GZFL-L, GZFL-H, and ibuprofen groups showed a shift toward the control group relative to the model group (Figure S5c and Figure 5c). Differential metabolites among groups were screened using OPLS-DA, and their overlap across pairwise group comparisons was visualized using a Venn diagram (Figure S5d). Metabolic pathway and enrichment analyses revealed alterations in several metabolic pathways, including taurine and hypotaurine metabolism, phenylalanine metabolism, vitamin B6 metabolism, and the TCA cycle (Figure 5e and Figure S5e). Heatmap visualization showed distinct alterations in endogenous metabolite profiles across groups (Figure 5d). Taurine, phenylalanine, FAD, and other differential metabolites showed notable changes across groups (Figure S8).

Spearman and Pearson correlation analyses were performed between metabolite levels and pharmacodynamic indicators, including the composite EI (Figure 5f and Figure S5f, S9 and S10). Based on the correlation analysis, 11 pharmacodynamic metabolites were selected for subsequent integration analysis, among which N-acetylglucosamine 1-phosphate, pyridoxine, and FAD showed relatively strong associations with EI (Table 7). Five integration strategies were then evaluated for their capacity to predict pharmacodynamic outcomes from metabolite data: PCA-based integration, metabolite efficacy index (MEI), metabolite SUM (SUM), rank-weighted SUM (R-SUM), and individual metabolite analysis. For PCA-based integration, the first three principal components were selected for weighting (Figure S5g). MEI was calculated according to Equation (4) where MEI denotes the metabolite efficacy index, ${\bar{C}}_{i}$ and ${\bar{M}}_{i}$ denote the mean levels of the *i*th metabolite in the control and model groups, respectively, and *GZFL_i_* represents its level in an individual sample from the treatment group.

(4) $$MEI=\sum_{i=1}^{n}\left|\frac{{GZFL}_{i}-{\bar{M}}_{i}}{{\bar{M}}_{i}-{\bar{C}}_{i}}\;\right|$$

SUM analysis involved direct summation of metabolite levels, whereas R-SUM incorporated correlation-weighted summation. Receiver operating characteristic (ROC) curves and area under the curve (AUC) values were calculated for each approach (Figure 5g). Among all methods evaluated, MEI achieved the highest AUC (0.9). MEI was therefore adopted as the preferred approach for characterizing GZFL efficacy markers and evaluating therapeutic response.

### 2.7. Chinmedomics Studies of GZFL in Rats

**Figure 6:**
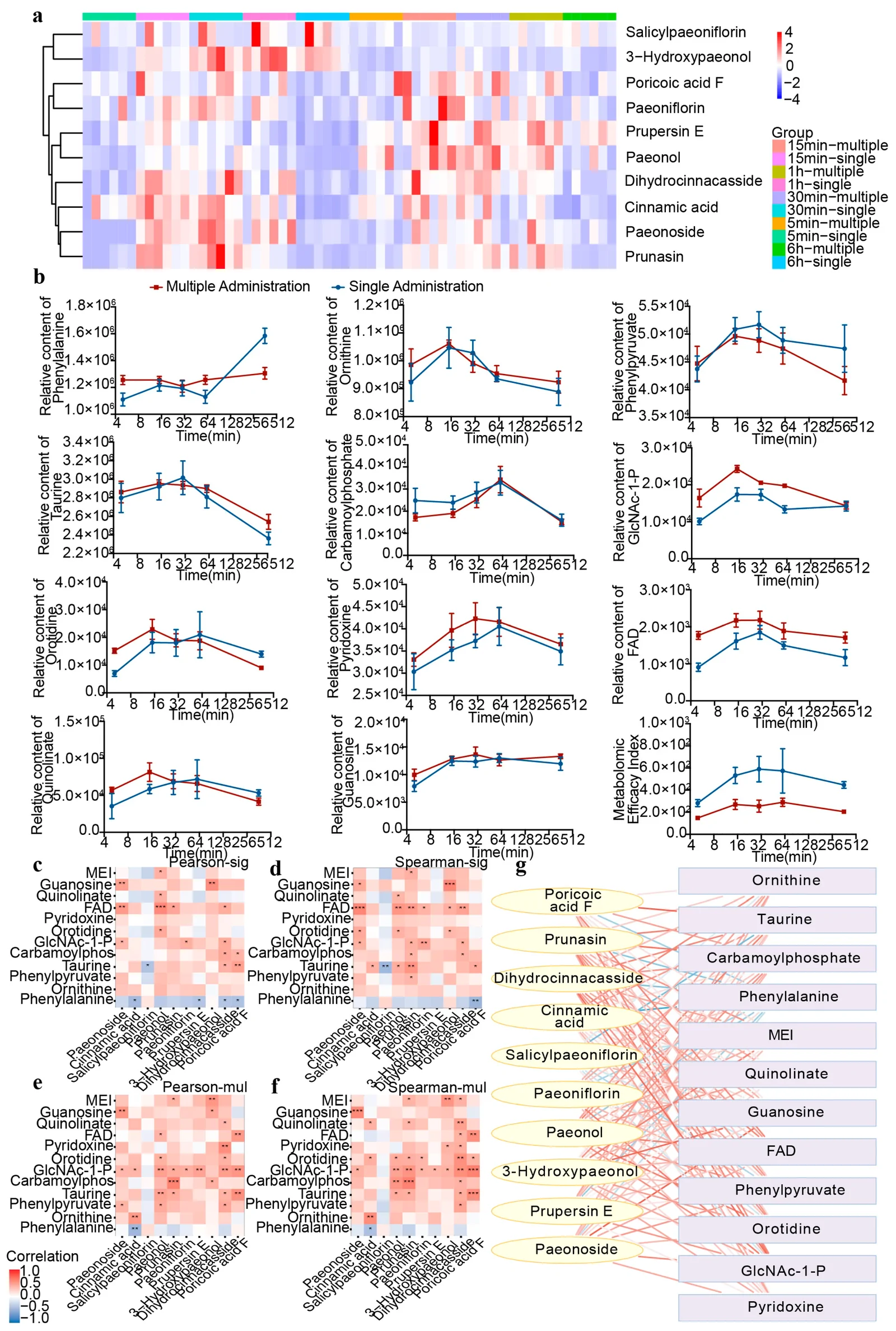
Chinmedomics investigation of GZFL. (**a**). Temporal profiles and hierarchical clustering of candidate bioactive constituents in serum following single- and multiple-dose administration. (**b**). Temporal abundance profiles of differential endogenous metabolites and the metabolite efficacy index (MEI) in serum following single- and multiple-dose administration. (**c**). Pearson correlation analysis between candidate bioactive constituents and differential endogenous metabolites following single-dose administration. (**d**). Spearman correlation analysis between candidate bioactive constituents and differential endogenous metabolites following single-dose administration. (**e**). Pearson correlation analysis between candidate bioactive constituents and differential endogenous metabolites following multiple-dose administration. (**f**). Spearman correlation analysis between candidate bioactive constituents and differential endogenous metabolites following multiple-dose administration. (**g**). Correlation network between candidate bioactive constituents and differential endogenous metabolites. Data are presented as the mean ± SEM (*n* = 6). In (**c**–**f**), * *p* < 0.05, ** *p* < 0.01, and *** *p* < 0.001 indicate statistically significant correlations. Abbreviations: GZFL, Guizhi Fuling capsule; MEI, metabolite efficacy index; GlcNAc-1-P, N-acetylglucosamine 1-phosphate.

Relative serum exposure profiles of candidate active components following single and multiple oral doses of GZFL were semi-quantitatively characterized (Figure S11a). Hierarchical clustering of the resulting exposure–time data showed four major temporal patterns (Figure 6a). Prototype components such as paeonol showed rapid appearance in serum, accompanied by the formation of downstream metabolites. Notably, multiple administration maintained higher serum levels of most compounds than single administration, suggesting greater systemic exposure following repeated administration. Parallel semi-quantitative analysis of serum metabolites enabled calculation of MEI across time points (Figure 6b). PCA and PLS-DA analyses showed inter-group differences and distinct temporal trends in metabolite profiles following single and multiple administrations of GZFL (Figure S11b,c).

Several endogenous metabolites, including FAD, showed time-dependent changes in serum following GZFL administration, with some reaching peak levels within 15 to 60 min post-dose. Pearson and Spearman correlation analyses were conducted between serum levels of candidate bioactive components and endogenous metabolite changes following single (Figure 6c,d) and multiple administrations (Figure 6e,f) of GZFL. Paeonoside, paeonol, dihydrocinnacasside, and 3-hydroxypaeonol showed significant correlations with endogenous metabolite changes. The resulting constituent–metabolite association network comprised 120 edges connecting 10 active components to 12 endogenous metabolites, demonstrating extensive associations between circulating GZFL constituents and endogenous metabolic changes (Figure 6g).

### 2.8. Temporal Dynamics of Disease Progression in Acute Dysmenorrhea Rats

**Figure 7:**
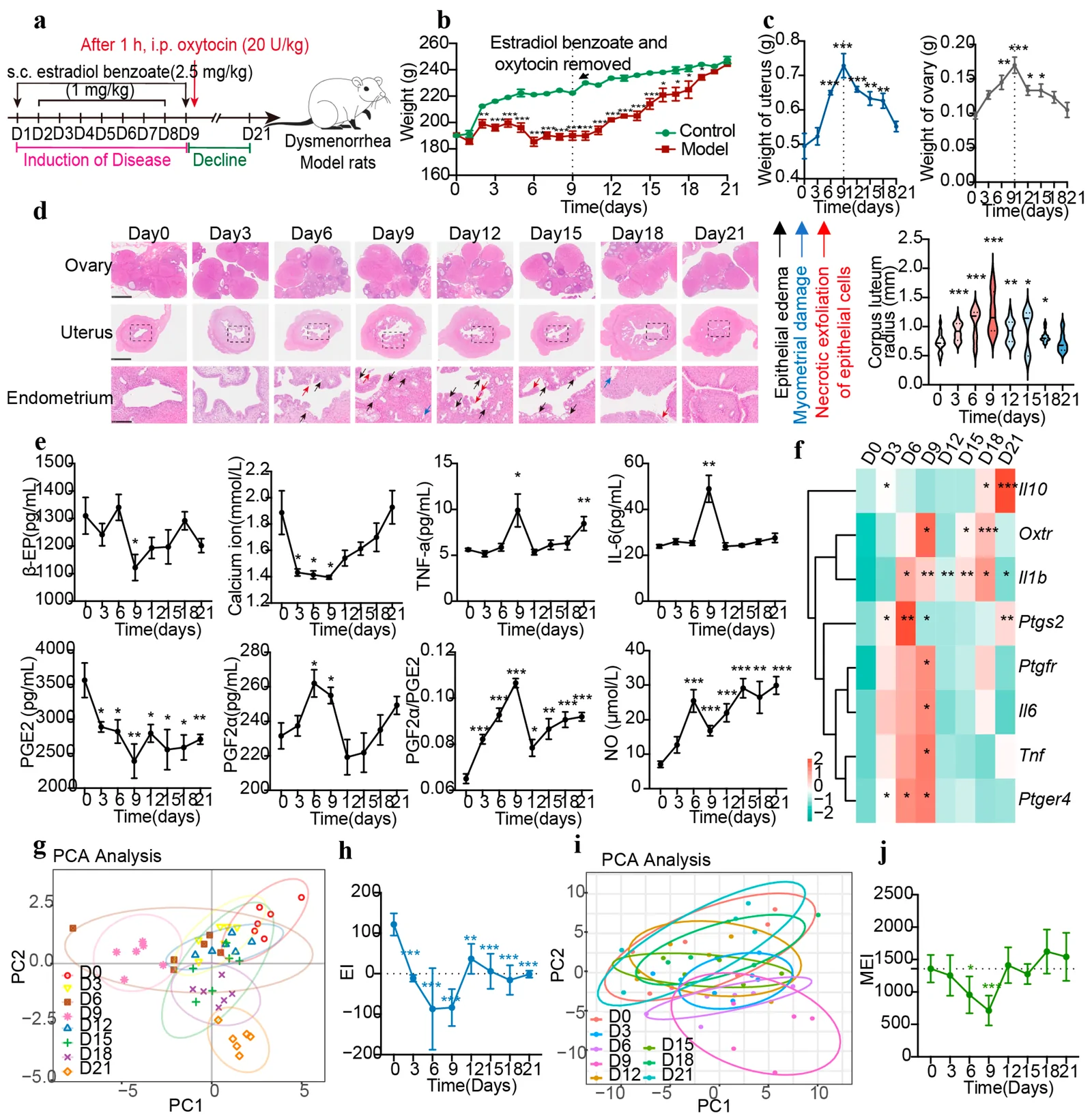
Disease progression in acute dysmenorrhea rats. (**a**). Schematic of the 21-day disease progression study in acute dysmenorrhea rats. (**b**). Time-course changes in body weight during disease progression. (**c**). Time-course changes in uterine and ovarian weights. (**d**). Representative histopathological sections of ovarian and uterine tissues at successive disease time points and quantitative analysis of corpus luteum radius (ovary and uterus: scale bar = 400 μm; endometrium: scale bar = 200 μm). (**e**). Time-course changes in serum biochemical indicators. (**f**). Time-course changes in inflammation-related mRNA expression in uterine tissue. (**g**). PCA score plot of integrated pharmacodynamic indicators at successive disease time points. (**h**). Time-course changes in the efficacy index (EI) during disease progression. (**i**). PCA score plot of serum metabolomic profiles at successive disease time points. (**j**). Time-course changes in the metabolite efficacy index (MEI) during disease progression. Data are presented as the mean ± SEM (*n* = 6). Compared with Day 0 (D0): * *p* < 0.05, ** *p* < 0.01, and *** *p* < 0.001. In (**d**), the dotted boxes indicate the regions shown at higher magnification. Abbreviations: PCA, principal component analysis; EI, efficacy index; MEI, metabolite efficacy index.

To characterize the temporal progression and recovery of dysmenorrhea, key indices were monitored across both the disease induction phase (days 0–9) and the spontaneous recovery phase (days 9–21) (Figure 7a). Body weight gain was suppressed during model induction and gradually recovered after withdrawal of the modeling stimulus, approaching control levels between days 18 and 21 (Figure 7b). Uterine and ovarian weights increased markedly after modeling, peaked at day 9, and approached normal values by days 18 and 21, respectively (Figure 7c). Histological analysis (Figure 7d) demonstrated progressive disruption of follicular architecture and uterine structure from day 0 to day 9, characterized by increasing follicular disorganization, corpus luteum enlargement, atretic follicle accumulation, irregular uterine lumen, and endometrial hyperplasia. These pathological changes were gradually ameliorated following withdrawal of the modeling stimulus between days 9 and 21.

Serum concentrations of NO, IL-6, TNF-α, β-EP, calcium, and prostaglandins all showed marked changes during the modeling phase. While serum calcium, IL-6, and TNF-α returned toward baseline during the recovery phase, NO levels and the PGF2α/PGE2 ratio remained persistently elevated (Figure 7e). At the transcriptional level, inflammation- and uterine contraction-related genes showed distinct temporal expression patterns during disease progression and recovery. Most genes showed marked alterations during the modeling phase followed by varying degrees of recovery after withdrawal of the modeling stimulus. Notably, *Ptgs2* showed an early increase followed by a second increase at day 21, while *Il10* showed a pronounced increase at day 21 (Figure 7f). A similar recovery pattern was observed in the LPS-induced RAW264.7 cell model (Figure S12).

PCA of the integrated pharmacodynamic indicators across the disease time course showed clear displacement of the day 9 cluster relative to day 0, with a partial but incomplete return toward baseline at day 21. Weighting of the first seven principal components yielded composite EI scores across the time course (Figure 7g). EI declined progressively during the induction phase, reaching its lowest level at day 9, followed by a marked rebound at day 12 and subsequent fluctuations without complete restoration to baseline levels from days 15 to 21 (Figure 7h). Complementary endogenous metabolome analysis by PCA revealed marked day 9 divergence from day 0, with day 21 metabolite profiles partially returning toward baseline (Figure 7i). MEI decreased progressively during model induction and reached its lowest level at day 9. Following withdrawal of the modeling stimulus, MEI increased markedly from day 12 and remained close to baseline levels during the later recovery phase from day 15 to day 21 (Figure 7j).

### 2.9. Transcriptomics and Molecule Docking

**Figure 8:**
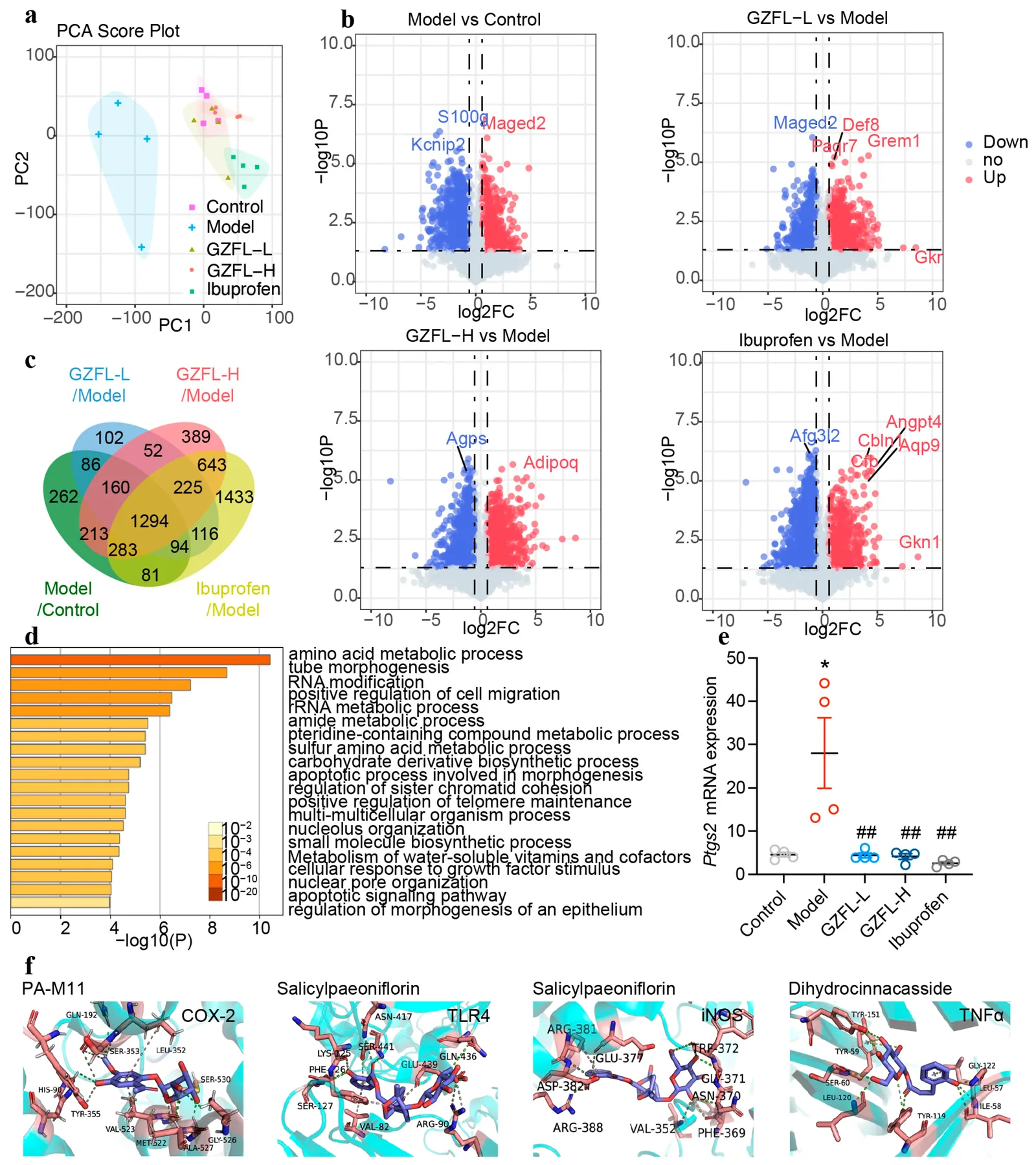
Transcriptomic analysis and molecular docking of dysmenorrhea-related target proteins with candidate GZFL bioactive constituents. (**a**). PCA score plot of transcriptomic profiles across experimental groups. (**b**). Volcano plots depicting differentially expressed mRNAs between pairwise group comparisons. (**c**). Venn diagram of differentially expressed mRNAs across groups. (**d**). Enrichment analysis of transcriptomic data. (**e**). *Ptgs2* mRNA expression levels across experimental groups. (**f**). Representative molecular docking interaction profiles between dysmenorrhea-associated target proteins and candidate GZFL bioactive constituents. Data are presented as the mean ± SEM (*n* = 4). Compared with the control group: * *p* < 0.05, compared with the model group: *p* < 0.05, ## *p* < 0.01, *p* < 0.001. Abbreviations: GZFL, Guizhi Fuling capsule; PCA, principal component analysis; *Ptgs2*, prostaglandin-endoperoxide synthase 2; COX-2, cyclooxygenase-2; TLR4, Toll-like receptor 4; iNOS, inducible nitric oxide synthase; TNF-α, tumor necrosis factor alpha.

The transcriptomic PCA score plot showed distinct clustering patterns across the experimental groups (Figure 8a). The model group exhibited marked transcriptomic divergence from the control group, whereas the treatment groups showed distinct transcriptomic shifts relative to the model group, with the GZFL-treated groups showing profiles closer to that of the control group. Volcano plot analysis (Figure 8b) revealed that, relative to the control group, 1018 and 1460 mRNAs were significantly down-regulated and up-regulated, respectively, in the model group. Upon comparison with the model group, the GZFL-L group demonstrated 1443 down-regulated and 689 up-regulated mRNAs, the GZFL-H group exhibited 2386 down-regulated and 879 up-regulated mRNAs, and the ibuprofen-treated group showed 3553 down-regulated and 622 up-regulated mRNAs.

Intersection analysis of differentially expressed mRNAs (Figure 8c) combined with functional enrichment analysis (Figure 8d and Figure S13a) indicated that GZFL modulated multiple biological processes in dysmenorrhea rats, including amino acid metabolism, epithelial morphogenesis, water-soluble vitamin and cofactor metabolism, carbohydrate derivative biosynthesis, and apoptosis-related processes. Notably, *Ptgs2* mRNA expression (Figure 8e) was significantly elevated in the uterine tissue of the model group and was markedly reduced in all treatment groups.

To explore the molecular basis of GZFL’s multi-target activity, molecular docking was performed for candidate bioactive constituents against dysmenorrhea-associated target proteins (Figure 8f and Figure S13b–f, Table 8). Computational modeling predicted that compounds including paeonoside, salicylpaeoniflorin, prunasin, paeoniflorin, prupersin E, dihydrocinnacasside, PA-M9, and PA-M11 may interact with key disease-relevant targets, including COX-2, TLR4, iNOS, TNF-α, PTGER4, PTGFR, PGR, and OXTR, as indicated by their predicted docking energies. Furthermore, compared with the reference prototype compounds cinnamic acid and paeonol, the ingredients paeonoside, PA-M11, and dihydrocinnacasside showed lower predicted docking energies. These compounds also showed relatively high serum exposure and were supported by the preceding pharmacodynamic analyses. These findings generate testable hypotheses regarding the molecular targets of key GZFL metabolites. Experimental validation through binding assays will be required to confirm these predicted interactions. Taken together, the transcriptomic and molecular docking data provide a preliminary mechanistic framework consistent with a multi-component, multi-target mode of action for GZFL in primary dysmenorrhea.

## 3. Discussion

The present study provides systematic evidence supporting the traditional TCM-documented use of GZFL for the treatment of blood stasis and menstrual pain. In classical TCM pathology, dysmenorrhea is associated with impaired Qi and blood circulation or insufficient nourishment of the uterus, whereas contemporary biomedical studies implicate excessive uterine smooth muscle contraction, prostaglandin imbalance, and inflammatory responses in its pathophysiology. GZFL suppressed NF-κB/IKKβ signalling, down-regulated COX-2 and iNOS, restored the PGF2α/PGE2 ratio, and normalized serum inflammatory cytokines, collectively indicating coordinated regulation of inflammatory and prostaglandin-related responses. Moreover, the bidirectional regulation of uterine contractility by GZFL-containing serum suggests that GZFL may modulate uterine function toward a more balanced physiological state rather than exerting a purely inhibitory effect. The prominent presence of biotransformation-derived metabolites in serum further suggests that circulating metabolites may contribute substantially to the pharmacologically active material basis of orally administered GZFL.

TCM is a complex therapeutic system characterized by multi-component formulations and multi-target pharmacological actions [27]. In this study, we integrated complementary “top-down” and “bottom-up” research paradigms to elucidate the material basis of GZFL [28,29]. The top-down strategy, proceeding from holistic formulation efficacy through systematic chemical characterisation and serum pharmacochemistry, narrowed 197 characterized components to 136 circulating constituents. The bottom-up strategy, based on network pharmacology and component–disease–target interaction networks, revealed 68 candidate therapeutic targets and 790 compound–target interactions, underscoring the multi-component, multi-target nature of GZFL’s mechanism. This integrative approach simultaneously reduced the compositional complexity of GZFL and preserved the holistic integrity inherent to compound TCM formulation research [30].

A central finding of this study is the prominent contribution of GZFL metabolites to its pharmacologically active material basis. Paeonoside and 3-hydroxypaeonol arise from the biotransformation of paeonol; salicylpaeoniflorin from paeoniflorin; prunasin from amygdalin; and dihydrocinnacasside from cinnamic acid. The serum ratios of these metabolites to their parent compounds were substantially elevated relative to those in the GZFL extract, suggesting that in vivo absorption and biotransformation altered the relative abundance of these constituents in the systemic circulation. This finding provides pharmacokinetic insight into the contribution of circulating constituents following oral administration of GZFL. Although the oral bioavailability of several parent compounds is limited, the increased relative abundance of their metabolites in serum, together with their pharmacodynamic associations and predicted interactions with disease-relevant targets, suggests that these circulating metabolites may contribute importantly to the systemic pharmacological effects of GZFL [5].

Whereas conventional NSAIDs primarily provide symptomatic relief through cyclooxygenase inhibition, GZFL modulated the upstream NF-κB/IKKβ signalling pathway, suppressing phosphorylation of IKKβ and p65, restoring IκBα expression, and normalizing COX-2 and iNOS levels, suggesting broader regulation of the pathophysiological processes underlying dysmenorrhea. Rather than simply suppressing prostaglandin production, GZFL appeared to promote restoration of prostaglandin homeostasis by normalizing COX-2 expression and correcting the elevated PGF2α/PGE2 ratio. Given the opposing roles of PGF2α and PGE2 in regulating uterine contractility, restoration of this balance may contribute to normalization of uterine contractile function rather than indiscriminate suppression of prostaglandin signaling. Moreover, GZFL restored estrous cyclicity in treated rats, an effect that was less evident with ibuprofen, suggesting broader regulation of reproductive function beyond symptomatic relief. Together, these findings provide a pharmacological basis for the traditional therapeutic concept of addressing both symptomatic manifestations and underlying pathological processes through coordinated regulation of inflammatory signaling, prostaglandin homeostasis, uterine contractility, and reproductive function.

A key methodological contribution of this study is the development of two composite indices for evaluating TCM therapeutic efficacy, based on the animal model established through a sequential sensitization-then-pain-induction protocol [31]. The Efficacy Index (EI), derived by PCA-based dimensionality reduction [32] of 23 pharmacodynamic indicators, provided an integrated measure of pharmacodynamic response. However, limitations of EI became apparent in longitudinal follow-up studies. During the spontaneous recovery phase (days 9–21), EI remained influenced by indicators with persistent or biphasic changes, including *Ptgs2*, NO, and *Il10*, and therefore did not fully reflect the later spontaneous recovery of the disease state. Integration of metabolomic alterations with pharmacodynamic indicators revealed 11 efficacy-associated endogenous metabolites, among which N-acetylglucosamine 1-phosphate, pyridoxine, and FAD showed relatively strong associations with EI, providing the basis for construction of the MEI. The Metabolite–Efficacy Index (MEI), derived from correlation analysis and chinmedomics investigation, addressed these limitations, achieving an AUC of 0.9 in ROC analysis, higher than those of the other evaluated strategies, and robustly tracked the full disease-recovery trajectory across the 21-day time course. Importantly, MEI was less affected by the biphasic behaviour of *Il10* and *Ptgs2*, more accurately capturing both disease progression and spontaneous recovery. These findings support MEI as a more integrative indicator of disease state than individual biomarkers or conventional pharmacodynamic endpoints and suggest its potential utility for evaluating the dynamic efficacy of complex therapies.

Semi-quantitative serum exposure analysis following single and multiple administrations showed that multiple dosing of GZFL sustained higher serum levels of most constituents, suggesting that repeated administration may enhance systemic exposure to circulating GZFL constituents. Pearson and Spearman correlation analyses between serum constituent concentrations and endogenous metabolite dynamics highlighted paeonoside, paeonol, dihydrocinnacasside, and 3-hydroxypaeonol as key constituents associated with changes in pharmacodynamically relevant metabolites including FAD, taurine, and phenylalanine. The resulting active-ingredient–metabolite association network narrows the candidate space of pharmacologically active GZFL constituents, providing a foundation for subsequent target identification and mechanistic validation.

Transcriptomic and molecular docking analyses provided additional support for the proposed multi-target mechanism of GZFL. Transcriptomic profiling showed that GZFL principally modulated amino acid metabolism, water-soluble vitamin and cofactor metabolism, and carbohydrate biosynthetic pathways, which were pertinent to the endogenous metabolite perturbations observed in the pharmacometabolomics analysis.

In silico docking predicted that several circulating constituents, including salicylpaeoniflorin, prupersin E, PA-M11, paeoniflorin, dihydrocinnacasside, and paeonoside, may interact with multiple dysmenorrhea-associated targets. Notably, paeonoside and dihydrocinnacasside were among the abundant serum-absorbed constituents and also showed associations with pharmacodynamically relevant endogenous metabolite changes, providing convergent evidence for their potential contribution to the pharmacological effects of GZFL. Salicylpaeoniflorin also showed relatively high serum abundance together with favorable predicted interactions across multiple targets, further supporting its potential pharmacological relevance. These predicted interactions warrant further experimental validation.

The detection of the cyanogenic glycosides prupersin and prupersin E among the circulating constituents of GZFL also warrants consideration of their potential toxicological relevance. Given their potential to release hydrogen cyanide upon enzymatic hydrolysis, systemic exposure to these constituents should be considered in the safety evaluation of GZFL. Although no overt signs of toxicity were observed during the present experimental period, this study was not designed to systematically evaluate the toxicological or toxicokinetic profiles of these constituents. Further studies characterizing their systemic exposure, metabolic fate, potential cyanide release, and toxicity following repeated administration are therefore warranted.

The present study advances prior GZFL research in several important respects. Firstly, the present findings highlight the prominent contribution of in vivo metabolites to the pharmacologically active material basis of GZFL. Secondly, our study characterized bidirectional uterine contractility regulation using GZFL-containing serum. Finally, MEI was proposed and evaluated as a dynamic, integrative efficacy indicator that more accurately captured the disease–recovery trajectory than the other evaluated strategies.

Several limitations of the present study should be acknowledged. First, the mechanistic origins of the differential metabolite-to-parent compound ratios between extract and serum require further characterization through comprehensive absorption, distribution, metabolism, and excretion studies. Second, the transcriptomic and molecular docking analyses are primarily hypothesis-generating and do not provide definitive evidence of target engagement or causal mechanisms. Further in vitro and in vivo studies using biophysical, genetic, and pharmacological approaches are required to validate the proposed molecular interactions and mechanisms. Third, the translatability of the rat PK-PM-PD findings to human clinical contexts requires validation through clinical pharmacokinetic and metabolomic studies. Finally, given the detection of cyanogenic glycosides among the circulating constituents, systematic toxicokinetic and safety evaluations are needed to characterize their metabolic fate, potential cyanide exposure, and risks associated with repeated administration.

## 4. Materials and Methods

### 4.1. Chemicals and Reagents

Guizhi Fuling capsule (GZFL; batch No. 180516 20540) was provided by Jiangsu Kanion Pharmaceutical Co., Ltd. (Lianyungang, China). Estradiol benzoate injection (veterinary drug approval No. 163232511), oxytocin injection (veterinary drug approval No. 140062778), and progesterone injection (veterinary drug approval No. 140061438) were purchased from Hefei Qianfang Animal Health Technology Co., Ltd. (Hefei, China). Ibuprofen (lot No. C10112589) was purchased from Macklin (Shanghai, China). Mass spectrometry-grade acetonitrile and methanol were obtained from Merck (Darmstadt, Germany). All remaining reagents were commercially available and of analytical grade.

### 4.2. Apparatus and Equipment

The following instruments were used: high-performance liquid chromatography system (LC-30AD, Shimadzu, Kyoto, Japan) coupled with a TripleTOF 5600 quadrupole time-of-flight mass spectrometer (AB SCIEX, Framingham, MA, USA); PowerWave X-type microplate reader (BioTek Instruments, Winooski, VT, USA); IEC low-temperature high-speed centrifuge (Thermo Fisher Scientific, Waltham, MA, USA); Sorvall Biofuge Stratos high-speed refrigerated centrifuge (Thermo Fisher Scientific, Waltham, MA, USA); Milli-Q Gradient A10 ultrapure water purification system (Millipore, Burlington, MA, USA); MiniSpin high-speed centrifuge (Eppendorf, Hamburg, Germany); Synergy H1 microplate reader (BioTek, VT, USA); AUW120D and AW120 electronic analytical balances (Shimadzu, Kyoto, Japan); GENIE VORTEX-2 mixer (Scientific Industries, Bohemia, NY, USA); Mini-PROTEAN^®^ Tetra Cell electrophoresis system (Bio-Rad, Hercules, CA, USA); and ChemiDoc XRS+ Gel Imaging System (Bio-Rad, CA, USA).

### 4.3. Characterization of GZFL Constituents in Extracts and Drug-Containing Rat Serum

#### 4.3.1. Animals and Treatments

Twenty-four healthy female Wistar rats, weighing approximately 180–220 g, were provided by SPF (Suzhou) Biotechnology Co., Ltd. (Suzhou, China; Experimental Animal Production Certificate A202406070044). Animals were housed under controlled conditions at a constant temperature of 22 ± 2 °C and relative humidity of 50 ± 10%. All animals were acclimatized for one week prior to experimentation. All procedures received ethical approval from the Animal Ethics Committee of China Pharmaceutical University. Animals in the control group received CMC-Na by oral gavage, while those in the treatment group received GZFL suspension. Thirty minutes after administration, blood was collected; whole blood was allowed to stand at room temperature for 30 min, followed by centrifugation at 8000 rpm for 5 min. The resulting supernatant was collected as drug-containing serum, and serum samples from animals within the same group were pooled and mixed.

#### 4.3.2. Sample Processing and Data Analysis

GZFL contents were extracted by ultrasonication in 50% methanol–water. 10 μL of GZFL extract was diluted 100-fold with ultrapure water, vortexed for 5 min, and centrifuged at 18,000 rpm at 4 °C for 5 min. 120 μL of supernatant was transferred and re-centrifuged under the same conditions; 80 μL of the resulting supernatant was then transferred to an autosampler vial for LC-MS analysis. For drug-containing serum, 200 μL of serum was combined with 600 μL of methanol, vortexed for 5 min, and centrifuged at 18,000 rpm at 4 °C for 5 min. 120 μL of supernatant was transferred and re-centrifuged; 80 μL of the resulting supernatant was transferred to an autosampler vial for LC-MS analysis.

Chemical constituents of the five herbs comprising GZFL were retrieved from CNKI, PubMed, Web of Science, and ChemSpider, and an in-house GZFL chemical database was constructed containing compound names, molecular formulae, exact molecular weights, and structural formulae [33,34,35,36,37,38,39,40]. Data acquisition was performed using Analyst software (version 1.7.1, AB SCIEX, Framingham, MA, USA). PeakView software (version 1.2, AB SCIEX, Framingham, MA, USA) was used to match acquired spectra against this database, together with the diagnostic ion network analysis (DINA) strategy [41]. Candidate peaks with a mass error within ±15 ppm were selected. Compound assignments were confirmed by comparison with the corresponding standards where authentic reference standards were available. For compounds without authentic standards, structural assignments were considered tentative and were based on accurate mass, isotope distribution, and MS/MS fragmentation patterns. Detailed MS/MS fragmentation analyses of representative constituents are provided in Supplementary Note S1. Detailed LC-MS/MS conditions are provided in Supplementary Method S1.

#### 4.3.3. Metabolism of GZFL in Primary Human Hepatocytes

Primary human hepatocytes (Lot No. 386549; Liver Biotechnology (Shenzhen) Co., Ltd., Shenzhen, China) were seeded onto collagen-coated 48-well plates and maintained at 37 °C with 5% CO_2_. After attachment, the cells were equilibrated in serum-free maintenance medium for 4 h and subsequently incubated with GZFL (80 μg/mL) for 2 h. The culture medium and hepatocytes were collected separately and subjected to protein precipitation with acetonitrile. After centrifugation, the supernatants were evaporated to dryness, reconstituted in 50% methanol–water, and analyzed by UPLC/Q-TOF-MS. Metabolites were tentatively assigned based on accurate mass and MS/MS fragmentation patterns.

### 4.4. Effect of GZFL on the Contractile Rhythm of Isolated Rat Uterus

An ex vivo rat uterine contractility model was established using a BL-420S tension transducer (Chengdu Taimeng Technology Co., Ltd., Chengdu, China) coupled with a BL-NewCentury biosignal acquisition system (Chengdu Taimeng Technology Co., Ltd., Chengdu, China). Uterine hypercontractility was induced by oxytocin, with saline serving as the negative control and verapamil as the positive control. A uterine hypocontractility model was additionally established by progesterone treatment, with saline as the negative control and oxytocin as the positive control. Blank rat serum supplemented with GZFL extract, GZFL-containing serum, and ibuprofen were each added to the organ bath to evaluate changes in uterine contraction frequency. Uterine contraction frequency was determined based on the number of contractions recorded over the observation period and expressed relative to that of the control group, which was normalized to 100%.

### 4.5. Network Pharmacology Analysis

Potential targets of the active constituents of GZFL were retrieved from the TCMSP database, SwissTargetPrediction, and other relevant databases. Targets associated with primary dysmenorrhea were obtained and curated using the GeneCards database. Protein–protein interaction (PPI) networks were constructed using the STRING platform. Enrichment analyses were conducted using the Metascape and Bioinformatics platforms, and the herb–active ingredient–target–signaling pathway network was visualized using Cytoscape software (version 3.10.3, Cytoscape Consortium, San Diego, CA, USA).

### 4.6. Dysmenorrhea Rat Model and Treatments

Thirty healthy female Wistar rats, weighing approximately 180–220 g, were provided by SPF (Suzhou) Biotechnology Co., Ltd. (Suzhou, China) (Experimental Animal Production Certificate A202406070044) and housed under controlled conditions at 22 ± 2 °C and 50 ± 10% relative humidity. Animals were acclimatized for one week prior to experimentation. Ethical approval was obtained from the Animal Ethics Committee of China Pharmaceutical University.

The acute dysmenorrhea model was established over a 9-day period. Rats were randomly allocated into five groups (*n* = 6 per group): control, model, GZFL low-dose (GZFL-L), GZFL high-dose (GZFL-H), and ibuprofen. Animals in the GZFL-L, GZFL-H, and ibuprofen groups received once-daily intragastric administration of the respective drugs suspended in CMC-Na (GZFL-L: 0.5 g/kg; GZFL-H: 1 g/kg; ibuprofen: 0.12 g/kg) for 9 consecutive days; control and model group animals received CMC-Na vehicle at equivalent volumes. Estradiol benzoate was administered subcutaneously into the dorsal neck region on days 1 and 9 at 2.5 mg/kg, and at 1 mg/kg from days 2 through 8. Prophylactic drug administration was performed 0.5 h after the subcutaneous estradiol benzoate injection each day. On day 9, oxytocin (2 U/kg) was administered intraperitoneally 1 h after the final estradiol benzoate injection. Three rats per group were selected for vaginal smear preparation to monitor estrous cyclicity. Following oxytocin injection on day 9, writhing frequency and latency were recorded over 30 min. All animals were then euthanized; uterine and ovarian tissues were collected and weighed. Portions of uterine and ovarian tissue were fixed in paraformaldehyde for hematoxylin and eosin (H&E) staining, and tissue morphology was examined using a Leica DMI3000 B microscope (Leica, Wetzlar, Germany). Corpus luteum radius was quantified using ImageJ software (version 1.53, National Institutes of Health, Bethesda, MD, USA). Remaining tissues and serum were collected for subsequent analyses. Additionally, a cold coagulation dysmenorrhea model was similarly established, with animals subjected to 2 h of cold stimulation daily.

### 4.7. Determination of Biochemical Indicators in Serum

Serum concentrations of β-endorphin (β-EP, E-EL-R0105), prostaglandin E2 (PGE2, E-EL-0034), prostaglandin F2α (PGF2α, E-EL-R0795), interleukin-6 (IL-6, E-EL-R0015), IL-1β (E-EL-R0012), and tumor necrosis factor-alpha (TNF-α, E-EL-R2856) were determined using commercially available ELISA kits from Elabscience Biotechnology Co., Ltd. (Wuhan, China), following the respective manufacturers’ instructions. Nitric oxide levels were measured using an assay kit (S0021M) from Beyotime (Shanghai, China). Serum calcium concentrations were determined using an assay kit (C004-2-1) from Nanjing Jiancheng Bioengineering Institute (Nanjing, China).

### 4.8. RNA Extraction and Quantitative Real-Time PCR

Total RNA was extracted from uterine tissue using TRIzol reagent (R401-01, Vazyme, Nanjing, China). Reverse transcription was performed using HiScript III Reverse Transcriptase SuperMix (R323-01, Vazyme, Nanjing, China) for qPCR in a T100 Thermal Cycler (Bio-Rad, Hercules, CA, USA), with the following cycling conditions: 37 °C for 15 min, followed by 85 °C for 5 s. Quantitative PCR reactions were prepared by combining 1 μL cDNA, 1 μL each of forward and reverse primers, 7.5 μL ChamQ SYBR qPCR Master Mix (Q311-02, Vazyme, Nanjing, China), and 4.5 μL DEPC water (R0022, Beyotime, Shanghai, China), and were run on a CFX96™ Real-Time PCR System (Bio-Rad, Hercules, CA, USA). PCR cycling conditions consisted of denaturation at 95 °C for 10 s, annealing at 60 °C for 30 s, and extension at 72 °C for 30 s, for a total of 40 cycles. Primer sequences (Invitrogen, Waltham, MA, USA) are provided in Table 9.

### 4.9. Western Blot Analysis

Uterine tissue samples (20 mg per sample) were homogenized in RIPA lysis buffer (Beyotime, Shanghai, China) supplemented with phenylmethylsulfonyl fluoride (PMSF, Beyotime, Shanghai, China) to extract total protein. Protein concentrations were determined using a BCA protein assay kit (P0009, Beyotime, Shanghai, China). Equal quantities of protein were resolved by SDS-PAGE using a 10% Color PAGE Gel Rapid Preparation Kit (Epizyme Biotech, Shanghai, China) and subsequently transferred to Immun-Blot^®^ PVDF membranes (Bio-Rad, CA, USA). Following blocking, membranes were incubated overnight at 4 °C with the respective primary antibodies. Primary antibodies against COX-2 (#12282), IκBα (#4814), NF-κB p65 (#8242), Phospho-IKKα/β (#2697), IKKβ (#8943), and Phospho-NF-κB p65 (#30333) were obtained from Cell Signaling Technology (Danvers, MA, USA). Antibodies against iNOS (18985-1-AP) and GAPDH (60004-1-Ig) were purchased from Proteintech (Wuhan, China). Membranes were then incubated with the appropriate HRP-conjugated secondary antibodies (BS12478, BS13278, Bioworld Technology Co., Ltd., Bloomington, MN, USA) for 2 h at room temperature. Immunoreactive bands were visualized using Clarity™ Western ECL Substrate (Bio-Rad, CA, USA). Band intensities were quantified using ImageJ software (version 1.53).

### 4.10. Pharmacometabolomics Analysis

In total, 50 μL of serum from each rat was transferred into clean microcentrifuge tubes. In total, 200 μL of precipitation solution (methanol containing 15 μg/mL [^13^C]-glutamine [Cambridge Isotope Laboratories, Tewksbury, MA, USA]) was added to each sample, followed by vortexing for 5 min and centrifugation at 20,000× *g* for 10 min. In total, 200 μL of the resulting supernatant was transferred to a new tube and dried under vacuum using a SpeedVac concentrator (Thermo Savant, Waltham, MA, USA). Quality control (QC) samples were prepared by combining equal volumes of supernatant from all samples using identical processing conditions, to monitor instrument stability throughout the analytical run. Dried residues were reconstituted in 100 μL of ultrapure water. Reconstituted samples were centrifuged at 20,000× *g* for 10 min; 80 μL of the resulting supernatant was transferred to injection vials for analysis. Statistical analyses were performed using R version 4.3.3 (R Foundation for Statistical Computing, Vienna, Austria). Differential metabolites were selected based on a variable importance in projection (VIP) value > 1 and a *p*-value < 0.05. Pathway and enrichment analyses of differential metabolites were conducted via the MetaboAnalyst (version 5.0, Xia Lab, McGill University, Montreal, QC, Canada) web platform. Based on these results, correlation analyses were performed between the selected differential endogenous metabolites and pharmacodynamic indicators, as well as the composite Efficacy Index (EI). Detailed LC-MS/MS conditions are provided in Supplementary Method S1.

### 4.11. Chinmedomics Investigation of GZFL in a Rat Model

Twelve healthy female Wistar rats, weighing approximately 180–220 g, were procured from SPF (Suzhou) Biotechnology Co., Ltd. (Suzhou, China) and maintained under standardized housing conditions at a temperature of 22 ± 2 °C and relative humidity of 50 ± 10%. All animals underwent a one-week acclimatization period prior to the initiation of experimental procedures. All experimental protocols involving animals were reviewed and approved by the Institutional Animal Ethics Committee of China Pharmaceutical University. Following single or multiple oral administration of GZFL (1 g/kg; once daily for 7 consecutive days for multiple administration), blood samples were collected at 5 min, 15 min, 30 min, 1 h, and 6 h after the single or final dose, respectively, and serum was isolated. Serum samples were analyzed using the LC-MS-based constituent characterization and pharmacometabolomic procedures described in Section 4.3 and Section 4.10, respectively. Relative exposure profiles of GZFL-derived constituents and metabolomic datasets were subsequently subjected to Pearson and Spearman correlation analyses to explore associations between the temporal changes in GZFL-derived constituents and endogenous metabolites.

### 4.12. Transcriptome Analysis and Molecular Docking

In total, 50 mg of uterine tissue per rat was collected, immediately snap-frozen on dry ice to preserve RNA integrity, and submitted to GENE DENOVO Biotechnology Co., Ltd. (Guangzhou, China) for transcriptomic sequencing. Differentially expressed genes were screened using a fold-change threshold of 1.5 and *p* < 0.05. Enrichment analysis of the resultant gene expression data was performed using the Metascape bioinformatics platform. For molecular docking studies, all small-molecule ligands were preprocessed using Discovery Studio 2016 Client (BIOVIA, San Diego, CA, USA). The three-dimensional crystal structures of the target proteins, including 5KIR, 3FXI, 3E7G, 6OOY, 9M1H, 6M9T, 7CX3, 8GD9, 8XJL, 3ZRB, and 6TPK, were retrieved from the Research Collaboratory for Structural Bioinformatics Protein Data Bank (RCSB PDB). Molecular docking simulations were performed using iGEMDOCK v2.1 (National Chiao Tung University, Hsinchu, Taiwan, China) to assess potential binding interactions and binding modes between the target proteins and candidate compounds. Protein–ligand interactions were visualized and rendered using PyMOL molecular visualization software (version 3.1.8, Schrödinger, LLC, New York, NY, USA).

### 4.13. Statistical Analysis

All data are presented as the mean ± SEM; sample sizes (*n*) are indicated in the corresponding figure legends. Comparisons between two groups were performed using unpaired Student’s t-test, while differences among multiple groups were assessed by one-way analysis of variance (ANOVA) followed by Bonferroni post hoc correction. A *p*-value < 0.05 was considered statistically significant. Conventional statistical analyses were performed using GraphPad Prism software (version 10, GraphPad Software, Boston, MA, USA).

## 5. Conclusions

Following oral administration, GZFL undergoes extensive biotransformation, and the resulting circulating metabolites may contribute importantly to its pharmacologically active material basis. GZFL modulated multiple biological processes relevant to dysmenorrhea, including inflammatory signaling, prostaglandin homeostasis, uterine contractility, and endogenous metabolic disturbances. In particular, regulation of the NF-κB/IKKβ pathway and restoration of the PGF2α/PGE2 balance support a broader homeostatic action of GZFL beyond symptomatic relief. Integration of PK-PM-PD data enabled the development of EI and MEI as composite indices of dynamic pharmacodynamic responses, with MEI more accurately capturing the disease–recovery trajectory than the other evaluated strategies. Overall, the integrated PK-PM-PD approach provides a systematic strategy for investigating the pharmacologically active material basis and multi-target effects of GZFL. Future studies should further validate efficacy-associated endogenous metabolites and predicted molecular targets, characterize the toxicokinetic and safety profiles of cyanogenic constituents, and evaluate the clinical translational potential of these findings.

## 6. Patents

A Chinese patent application related to the metabolite–efficacy integration index developed in this study has been filed (Application No. 202611091453.9).

## Figures and Tables

**Table 1:** Attribution of GZFL components in the extract and rat serum.

	GZFL Extract	Rat Serum
GZ	51	40
FL	32	19
BS	134	71
MDP	59	37
TR	17	15
Common constituents	50	37
Total components	197	136

GZ: *Ramulus Cinnamomi*; FL: *Poria*; TR: *Semen Persicae*; MDP: *Cortex Moutan*; BS: *Radix Paeoniae Alba*.

**Table 2:** Chemical class distribution of serum-absorbed components of GZFL.

Chemical Class	*n*	%
Terpenoid glycosides	31	22.8
Terpenoids	25	18.4
Phenolic compounds	11	8.1
Phenolic glycosides	10	7.4
Flavonoid glycosides	9	6.6
Other glycosides	8	5.9
Phenolic acids	7	5.1
Fatty acids and derivatives	7	5.1
Phenylpropanoids	6	4.4
Phenylpropanoid glycosides	5	3.7
Lignans	4	2.9
Cyanogenic glycosides	3	2.2
Flavonoids	3	2.2
Others	3	2.2
Hydrolyzable tannins	2	1.5
Coumarins	1	0.7
Organic acids	1	0.7
Total	136	100.0 ^1^

^1^ Percentages may not sum to 100.0% due to rounding.

**Table 3:** Ten most abundant serum-absorbed components of GZFL based on peak area.

NO.	Compound	Molecular Formula	tR/min	Ion Mode	[M + H]+/[M − H]−		Error (10^−6^)	MS/MS (*m*/*z*)	Area	Attribution	Chemical Class
					Calculated *m*/*z*	Observed *m*/*z*					
1	Paeonoside	C_15_H_20_O_8_	16.54	[M − H]−	327.1080	327.1092	3.7	149.0697, 134.0404	108,484	BS, MDP	Phenolic glycosides
2	Cinnamic acid	C_9_H_8_O_2_	20.47	[M + H + H_2_O]+	167.0708	167.0710	1.2	149.0603, 121.0653, 103.0539, 91.0543, 77.0388	58,878	GZ	Phenylpropanoids
3	Salicylpaeoniflorin	C_23_H_28_O_12_	11.58	[M + NH_4_]+	514.1925	514.1941	3.1	179.0709, 151.0755, 139.0383, 133.0647	56,276	BS, MDP	Terpenoid glycosides
4	Paeonol	C_9_H_10_O_3_	19.93	[M − H]−	165.0552	165.0561	5.5	150.0326, 122.0374, 91.0194	46,692	BS, MDP	Phenolic compounds
5	Prunasin	C_14_H_17_NO_6_	12.85	[M + HCOO]−	340.1032	340.1046	4.1	161.0471	26,353	TR	Cyanogenic glycosides
6	Paeoniflorin	C_23_H_28_O_11_	12.55	[M + H]+	481.1710	481.1707	−0.6	375.1271, 123.0801	19,993	BS, MDP	Terpenoid glycosides
7	Prupersin E	C_26_H_30_N_2_O_12_	13.2	[M + H]+	563.1877	563.1926	8.7	106.0290, 340.1466	18,874	TR	Cyanogenic glycosides
8	3-Hydroxypaeonol	C_9_H_10_O_4_	15.28	[M − H]−	181.0501	181.0508	3.9	166.0242, 108.0173	18,622	MDP	Phenolic compounds
9	Dihydrocinnacasside	C_15_H_20_O_8_	12.83	[M − H]−	327.1080	327.1073	−2.1	165.0582, 147.0519, 121.0315, 113.0237, 103.0475, 89.0438, 71.0149, 59.0148	16,002	GZ	Phenylpropanoid glycosides
10	Poricoic acid F	C_31_H_46_O_5_	26.83	[M − H]−	497.3267	497.3252	−3.0	451.2699, 145.1094	12,766	FL	Terpenoids

GZ: *Ramulus Cinnamomi*; FL: *Poria*; TR: *Semen Persicae*; MDP: *Cortex Moutan*; BS: *Radix Paeoniae Alba*.

**Table 4:** UPLC/Q-TOF-MS characterization data for metabolites of paeonol, paeoniflorin and amygdalin in human primary hepatocytes.

Parents & Metabolites	t_R_/ min	Molecular	Ion Mode	Observed *m*/*z*	Calculated *m*/*z*	Error	Main Fragments *m*/*z*	Metabolite Description	LC-MS/MS Peak Area		
									Cell	Supernatant	Total
PA	12.04	C_9_H_10_O_3_	[M − H]−	165.0538	165.0552	−8.5	150.0317; 135.0070; 122.0362; 91.0179	Prototype	1.0 × 10^3^	4.3 × 10^3^	5.3 × 10^3^
PA-M1	12.78	C_15_H_18_O_9_	[M − H]−	341.0889	341.0873	4.7	165.0559; 150.0323; 135.0096; 122.0370	Glucuronidation	7.2 × 10^3^	7.7 × 10^3^	1.5 × 10^4^
PA-M2	12.54	C_14_H_16_O_9_	[M − H]−	327.0743	327.0716	8.2	151.0405; 135.0076; 109.0282; 91.0193	Demethylation + glucuronidation	1.4 × 10^3^	3.6 × 10^3^	5.0 × 10^3^
PA-M3	12.76	C_15_H_18_O_10_	[M − H]−	357.0839	357.0822	4.8	181.0501; 151.0068; 123.0705;	Mono-oxidation + glucuronidation	9.8 × 10^2^	7.4 × 10^3^	8.4 × 10^3^
PAE	12.78	C_23_H_28_O_11_	[M − H]−	479.1585	479.1553	6.7	327.1105; 165.0564; 121.0298; 77.0403	Prototype	ND	9.8 × 10^3^	9.8 × 10^3^
PAE-M1	12.86	C_9_H_9_NO_3_	[M − H]−	178.0514	178.0504	5.6	77.0387	Deglycosylation + cleavage + glycine conjugation	3.0 × 10^3^	3.6 × 10^3^	6.6 × 10^3^
PAE-M2	14.09	C_16_H_26_O_7_	[M − H]−	329.1603	329.1600	0.9	137.0689	Cleavage + ring opening	1.2 × 10^3^	3.2 × 10^3^	4.4 × 10^3^
AMY	11.93	C_20_H_27_NO_11_	[M − H]−	456.1494	456.1506	−2.6	323.0991; 263.0786; 245.0642; 179.0556; 161.0461	Prototype	4.5 × 10^2^	2.2 × 10^4^	2.2 × 10^4^
AMY-M1	3.19	C_12_H_22_O_12_	[M − H]−	357.1039	357.1033	1.7	179.0529	Cyanogenic moiety cleavage + oxidation	5.8 × 10^3^	ND	5.8 × 10^3^

PA: Paeonol; PAE: Paeoniflorin; AMY: Amygdalin. ND: Not Detected.

**Table 5:** Estrous cycle patterns of rats in different groups.

Day 1	Day 2	Day 3	Day 4	Day 5	Day 6	Day 7	Day 8	Day 9
proestrus	estrus	metestrus	diestrus	proestrus	estrus	metestrus	diestrus	diestrus
metestrus	proestrus	metestrus	diestrus	metestrus	metestrus	diestrus	metestrus	diestrus
diestrus	diestrus	estrus	metestrus	diestrus	diestrus	estrus	diestrus	diestrus
proestrus	estrus	metestrus	diestrus	diestrus	estrus	metestrus	diestrus	diestrus
metestrus	estrus	diestrus	diestrus	diestrus	metestrus	diestrus	metestrus	diestrus

**Table 6:** Eigenvalues and cumulative variance contribution of each principal component.

Component	Eigenvalue	Percentage (%)	Cumulative Percentage (%)
1	12.05	52.41	52.41
2	2.57	11.17	63.58
3	1.67	7.28	70.86
4	1.28	5.58	76.44
5	0.93	4.05	80.49
6	0.76	3.29	83.78
7	0.69	3	86.78
…	…	…	…
22	0.01	0.04	99.98
23	0	0.01	100

**Table 7:** Correlation coefficients between differential endogenous metabolites and EI.

Metabolites	Spearman Correlation		Pearson Correlation		Average Coefficient
	Coefficient	*p* Value	Coefficient	*p* Value	
Orotidine	0.32	0.08	0.47	0.01	0.4
Pyridoxine	−0.47	0.01	−0.52	0	−0.49
FAD	0.44	0.02	0.51	0	0.47
Quinolinate	0.37	0.05	0.44	0.02	0.4
Cysteine	0.15	0.44	0.28	0.14	0.21
Phenylalanine	0.28	0.14	0.47	0.01	0.37
Proline	0.23	0.23	0.27	0.16	0.25
Carbamoylphosphate	0.35	0.06	0.48	0.01	0.42
Taurine	0.34	0.07	0.44	0.01	0.39
Succinate	0.3	0.11	0.32	0.09	0.31
Myoinositol	0.18	0.35	0.26	0.16	0.22
Guanosine	0.33	0.07	0.4	0.03	0.37
N-Acetyl-glucosamine1-phosphate	0.52	0	0.56	0	0.54
Ornithine	0.45	0.01	0.43	0.02	0.44
Glycine	0.29	0.12	0.36	0.05	0.32
Phenylpyruvate	0.31	0.1	0.39	0.03	0.35

**Table 8:** Predicted docking energies of potential pharmacoactive components against dysmenorrhea-associated targets.

Compound	COX-2	TLR4	iNOS	TNF-α	PTGER1	PTGER2	PTGER3	PTGER4	PTGFR	PGR	OXTR
Paeonoside	−98.9	−96.2	−153.8	−119.9	−102.7	−114.0	−117.7	−116.1	−119.3	−102.5	−88.5
Cinnamic acid	−78.4	−70.4	−97.6	−62.1	−67.8	−67.0	−65.3	−64.1	−71.7	−66.2	−66.7
Salicylpaeoniflorin	−101.7	−152.2	−174.4	−118.6	−142.4	−137.3	−150.2	−135.4	−166.7	−130.8	−112.1
Paeonol	−81.6	−77.8	−97.3	−69.2	−69.4	−73.1	−74	−72.7	−73.3	−67.5	−69.6
Prunasin	−103.0	−111.1	−138.9	−109.7	−104.9	−97.2	−113.7	−100.2	−112.2	−99.7	−89.3
Paeoniflorin	−107.3	−121.5	−147.7	−108.8	−142.6	−135.7	−145.3	−130.1	−139.4	−125.1	−122.5
Prupersin E	−109.0	−132.7	−144.2	−99.2	−103.8	−123.2	−127.2	−133.2	−145.8	−103.5	−127.2
Dihydrocinnacasside	−109.2	−121.5	−148.6	−137.1	−112.8	−118.5	−119.8	−105.4	−116.2	−109.4	−89.6
Poricoic acid F	−90.8	−100.2	−116.2	−82.1	−77.3	−120.0	−117.1	−105.6	−145.3	−83.2	−99.1
PAM1	−84.0	−79.1	−104.7	−68.9	−65.7	−80.0	−70.7	−70.7	−70.4	−67.2	−60.5
PAM2	−82.8	−76.0	−97.6	−72.7	−76.3	−71.5	−71.7	−77.6	−73.3	−62.5	−69.6
PAM3	−86.5	−83.7	−108.2	−76.6	−75.4	−75.2	−76.9	−79.2	−77.8	−67.0	−65.1
PAM5	−93.4	−99.8	−136.8	−85.4	−89.0	−84.9	−88.3	−78.9	−89.6	−76.9	−78.1
PAM6	−98.9	−98.1	−142.0	−79.0	−86.6	−80.8	−83.6	−91.8	−90.7	−72.1	−78.2
PAM9	−112.0	−132.8	−139.6	−121.2	−102.7	−112.4	−109.4	−104.2	−115.9	−91.4	−84.0
PAM11	−122.2	−128.4	−144.6	−121.7	−112.1	−115.0	−113.0	−107.6	−122.7	−98.9	−104.1
CAM1	−85.0	−84.3	−104.8	−68.8	−77.8	−69.1	−73.4	−69.3	−78.4	−63.9	−69.2
3-Hydroxypaeonol	−93.6	−90.1	−114.7	−78.2	−72.4	−77.1	−76.1	−81.6	−81.2	−68.8	−69.9
Positive molecule	−100.0	−127.5	−141.0	−80.4	−92.7	−97.0	−103.9	−95.6	−100.3	−84.3	−103.3

**Table 9:** PCR primers nucleotide sequences of related genes.

Genes	5′-3′ Primer Sequences	
Oxtr	Forward	CGTACTGGCCTTCATCGTGT
	Reverse	CATTGACGTCCCAAACGCTC
Tnf	Forward	ATCCGAGATGTGGAACTGGC
	Reverse	CGATCACCCCGAAGTTCAGT
Il1b	Forward	GCAGTGTCACTCATTGTGGC
	Reverse	CACGGGTTCCATGGTGAAGT
Il6	Forward	CAGTTGCCTTCTTGGGACTGA
	Reverse	TGTGGGTGGTATCCTCTGTG
Ptger4	Forward	GGAGACCAGGCATTGTGTGA
	Reverse	CTTGTCCACGTAGTGGCTGT
Ptgfr	Forward	TCACAGACTTCTTCGGCCAC
	Reverse	GAAAAGTGGGCACAAGCCAG
Il10	Forward	CCCTGTAGCCACCCAACAAA
	Reverse	TTCACGTGTGATCGCCTCAA
Ptgs2	Forward	GTGGAAAAGCCTCGTCCAGA
	Reverse	TCCTCCGAAGGTGCTAGGTT
Actb	Forward	CCTAAGGCCAACCGTGAAAAGA
	Reverse	CCCTCATAGATGGGCACAGT

## Data Availability

The original contributions presented in this study are included in the article/Supplementary Materials. Further inquiries can be directed to the corresponding authors.

## Supplementary Materials

The following supporting information can be downloaded at https://www.mdpi.com/article/10.3390/ph19091438/s1. Table S1: Tentative annotation of chemical constituents in GZFL extract based on UPLC/Q-TOF-MS analysis; Table S2: Common components in the component-target network of GZFL; Supplementary Note S1: MS/MS fragmentation analysis of representative GZFL constituents; Supplementary Method S1: LC-MS/MS conditions; Figure S1: Supplementary characterization of the material basis, uterine contractile effects, and network pharmacological analysis of GZFL; Figure S2: Proposed metabolic pathways and UPLC/Q-TOF-MS characterization of paeonol and its metabolites in primary human hepatocytes; Figure S3: Proposed metabolic pathways and UPLC/Q-TOF-MS characterization of paeoniflorin and its metabolites in primary human hepatocytes; Figure S4: Proposed metabolic pathway and UPLC/Q-TOF-MS characterization of amygdalin and its metabolite in primary human hepatocytes; Figure S5: Supplementary pharmacodynamic and serum metabolomic analyses of GZFL in the acute dysmenorrhea rat model; Figure S6: In vivo efficacy of GZFL against dysmenorrhea in rats with cold coagulation and blood stasis; Figure S7: Effects of GZFL on inflammation-related gene expression and serum biochemical indicators in rats with cold coagulation and blood stasis dysmenorrhea; Figure S8: Changes in the relative levels of differential endogenous metabolites in serum following GZFL treatment; Figure S9: Spearman correlation analysis between differential endogenous metabolites and the integrated efficacy index (EI); Figure S10: Pearson correlation analysis between differential endogenous metabolites and the integrated efficacy index (EI); Figure S11: Changes in serum levels of potential active components and multivariate analysis of serum endogenous metabolites; Figure S12: Time-course changes in inflammatory responses in LPS-stimulated RAW264.7 cells; Figure S13: Metascape enrichment network and representative molecular docking interactions of GZFL-derived components with dysmenorrhea-associated targets.

## Abbreviations

The following abbreviations are used in this manuscript:

GZFL	Guizhi Fuling Capsule
TCM	Traditional Chinese Medicine
PGF2α	prostaglandin F2α
PGE2	prostaglandin E2
NF-κB	nuclear factor kappa B
IKKβ	IκB kinase β
COX-2	cyclooxygenase-2
iNOS	inducible nitric oxide synthase
PK-PM-PD	pharmacokinetic–pharmacometabolomic–pharmacodynamic
EI	Efficacy Index
MEI	Metabolite–Efficacy Index
UPLC/Q-TOF-MS	ultra-performance liquid chromatography/quadrupole time-of-flight mass spectrometry
DINA	diagnostic ion network analysis
PPI	protein–protein interaction
PCA	principal component analysis
PLS-DA/OPLS-DA	(orthogonal) partial least squares-discriminant analysis
KEGG	Kyoto Encyclopedia of Genes and Genomes
GO	Gene Ontology
ROC	receiver operating characteristic
AUC	area under the curve
VIP	variable importance in projection
GZFL-L/GZFL-H	GZFL low-dose/high-dose
QC	quality control
H&E	hematoxylin and eosin
β-EP	β-endorphin
NO	nitric oxide
TNF-α	tumor necrosis factor-alpha
IL-6/IL-1β/IL-10	interleukin-6/-1β/-10
