# Supplementary material for: Integrated Pharmacokinetics, Pharmacodynamics, and Pharmacometabolomics to Elucidate Guizhi Fuling Capsule’s Homeostatic Mechanism Against Acute Dysmenorrhea
Source: Pharmaceuticals (Basel). 2026 Sep 10;19(9):1438. doi: 10.3390/ph19091438 (PMC13610729; doi:10.3390/ph19091438)
Supplement: Supplementary file 1 [file pharmaceuticals-19-01438-s001.zip › pharmaceuticals-4489007-supplementary.pdf]

Table S1. Tentative annotation of chemical constituents in GZFL extract based on UPLC/Q-TOF-MS analysis

NO.	Compound	Molecular formula	t_R/min	Ion mode	$[M+H]^+/[M-H]^-$ Calculated *m/z*	Observed *m/z*	Error (10^{-6})	MS/MS (*m/z*)	Area	Attribution	Chemical class
1	Paeonoside	$C_{15}H_{20}O_8$	16.54	[M-H]-	327.1080	327.1092	3.7	149.0697, 134.0404	108484	BS, MDP	Phenolic glycosides
2	Cinnamic acid	$C_9H_8O_2$	20.47	$[M+H+H_2O]+$	167.0708	167.0710	1.2	149.0603, 121.0653, 103.0539, 91.0543, 77.0388	58878	GZ	Phenylpropanoids
3	Salicylpaeoniflorin	$C_{23}H_{28}O_{12}$	11.58	$[M+NH_4]+$	514.1925	514.1941	3.1	179.0709, 151.0755, 139.0383, 133.0647	56276	BS, MDP	Terpenoid glycosides
4	Paeonol	$C_9H_{10}O_3$	19.93	[M-H]-	165.0552	165.0561	5.5	150.0326, 122.0374, 91.0194	46692	BS, MDP	Phenolic compounds
5	Prunasin	$C_{14}H_{17}NO_6$	12.85	[M+HCOO]-	340.1032	340.1046	4.1	161.0471	26353	TR	Cyanogenic glycosides
6	Paeoniflorin	$C_{23}H_{28}O_{11}$	12.55	[M+H]+	481.1710	481.1707	-0.6	375.1271, 123.0801	19993	BS, MDP	Terpenoid glycosides
7	Prupersin E	$C_{26}H_{30}N_2O_{12}$	13.20	[M+H]+	563.1877	563.1926	8.7	106.0290, 340.1466	18874	TR	Cyanogenic glycosides

Continued Table S1 Tentative annotation of chemical constituents in GZFL extract based on UPLC/Q-TOF-MS analysis

NO.	Compound	Molecular formula	t_R/min	Ion mode	$[M+H]^+/[M-H]^-$ Calculated *m/z*	Observed *m/z*	Error (10^{-6})	MS/MS (*m/z*)	Area	Attribution	Chemical class
8	3-Hydroxypaeonol	$C_9H_{10}O_4$	15.28	[M-H]-	181.0501	181.0508	3.9	166.0242, 108.0173	18622	MDP	Phenolic compounds
9	Dihydrocinnacasside	$C_{15}H_{20}O_8$	12.83	[M-H]-	327.1080	327.1073	-2.1	165.0582, 147.0519, 121.0315, 113.0237, 103.0475, 89.0438,71.0149, 59.0148	16002	GZ	Phenylpropanoid glycosides
10	Poricoic acid F	$C_{31}H_{46}O_5$	26.83	[M-H]-	497.3267	497.3252	-3.0	451.2699, 145.1094	12766	FL	Terpenoids
11	Salicylic acid	$C_7H_6O_3$	12.46	[M-H]-	137.0239	137.0255	11.7	93.0353	12737	GZ	Phenolic acids
12	Ethyl 3,4-dihydroxy-5-[(3,4,5-trihydroxybenzoyl)oxy]benzoate	$C_{16}H_{14}O_9$	14.61	[M-H]-	349.0560	349.0568	2.3	197.0476, 169.0146, 124.0170	12714	BS	Phenolic compounds
13	6-Methylcoumarin or 7-Methylcoumarin	$C_{10}H_8O_2$	17.55	[M+H]+	161.0603	161.0597	-3.7	133.0657, 105.0696	12629	GZ	Coumarins

Continued Table S1 Tentative annotation of chemical constituents in GZFL extract based on UPLC/Q-TOF-MS analysis

NO.	Compound	Molecular formula	t_R/min	Ion mode	$[M+H]^+/[M-H]^-$		Error (10^{-6})	MS/MS (*m/z*)	Area	Attribution	Chemical class
					Calculated *m/z*	Observed *m/z*					
14	3β，4β，23-Trihydroxy-24，30-dinorolean-12，20 (29)-dien-28-oic acid	$C_{28}H_{42}O_5$	22.61	[M-H]-	457.2954	457.2964	2.2	/	11851	MDP	Terpenoids
15	Lactiflorin	$C_{23}H_{26}O_{10}$	14.10	[M+HCOO]-	507.1502	507.1461	-8.1	461.1497, 339.1097	11406	BS, MDP	Terpenoid glycosides
16	Abscisic acid	$C_{15}H_{20}O_4$	15.77	[M-H]-	263.1283	263.1295	4.6	219.1401, 153.0924, 111.0494	11153	GZ	Terpenoids
17	Syringaldehyde	$C_9H_{10}O_4$	15.30	[M-H]-	181.0501	181.0520	10.5	166.0300, 151.0056, 123.0079, 95.0378, 79.0543, 67.0142	10973	GZ	Phenolic compounds
18	Mascaroside	$C_{26}H_{36}O_{11}$	12.10	[M-H]-	523.2118	523.2164	8.8	493.1310, 475.6919, 375.1081, 345.1160, 165.0181	10732	BS	Terpenoid glycosides

Continued Table S1 Tentative annotation of chemical constituents in GZFL extract based on UPLC/Q-TOF-MS analysis

NO.	Compound	Molecular formula	t_R/min	Ion mode	$[M+H]^+/[M-H]^-$ Calculated *m/z*	Observed *m/z*	Error (10^{-6})	MS/MS (*m/z*)	Area	Attribution	Chemical class
19	13-HPODE(1-)	$C_{18}H_{32}O_4$	22.09	[M-H]-	311.2222	311.2234	3.9	293.2139, 275.2020, 235.1657, 223.1710, 87.0429	10602	GZ	Fatty acids and derivatives
20	Neochlorogenic acid	$C_{16}H_{18}O_9$	15.09	[M-H]-	353.0873	353.0879	1.7	179.0568, 135.0664	10602	GZ, BS	Phenylpropanoids
21	Picrocrocinic acid	$C_{16}H_{26}O_8$	13.69	[M-H]-	345.1549	345.1547	-0.6	179.0563, 165.0943, 59.0156	9432	BS	Terpenoid glycosides
22	Coumaric acid 2-glucoside or Coumaric acid 4-glucoside	$C_{15}H_{18}O_8$	19.29	[M-H]-	325.0923	325.0947	7.4	163.0352, 145.0253	9092	GZ	Phenylpropanoid glycosides
23	Dihydrocaffeic acid	$C_9H_{10}O_4$	11.56	[M-H]-	181.0501	181.0520	10.5	163.0409, 119.0511, 110.0361, 89.0366	9027	BS	Phenolic acids
24	Palmitic acid	$C_{16}H_{32}O_2$	29.49	[M-H]-	255.2324	255.2342	7.1	/	8692	all	Fatty acids and derivatives
25	Mudanpioside E	$C_{24}H_{30}O_{13}$	13.82	[M-H]-	525.1608	525.1621	2.5	479.1564, 449.1463	8655	MDP	Terpenoid glycosides

Continued Table S1 Tentative annotation of chemical constituents in GZFL extract based on UPLC/Q-TOF-MS analysis

NO.	Compound	Molecular formula	t_R/min	Ion mode	$[M+H]^+/[M-H]^-$ Calculated *m/z*	Observed *m/z*	Error (10^{-6})	MS/MS (*m/z*)	Area	Attribution	Chemical class
26	7-Caffeoylsedo-heptulose	$C_{16}H_{20}O_{10}$	11.83	[M-H]-	371.0978	371.0965	-3.5	230.9024, 121.0287	7991	GZ	Phenylpropanoids
27	6,16α-Dihydroxy-dehydroeburiconic acid	$C_{31}H_{46}O_5$	25.42	[M-H]-	497.3267	497.3263	-0.8	467.3185, 433.6417	7919	FL	Terpenoids
28	Polyporenic acid C	$C_{31}H_{46}O_4$	26.41	[M-H]-	481.3318	481.3309	-1.9	465.2975, 309.1921	7878	FL	Terpenoids
29	16α-Hydroxytra-metenolic acid	$C_{30}H_{48}O_4$	26.40	[M-H]-	471.3474	471.3492	3.8	455.2554, 437.2175, 313.1253, 295.1267	7546	FL	Terpenoids
30	Galactitol	$C_6H_{14}O_6$	2.99	[M-H]-	181.0712	181.0726	7.7	163.0678, 119.0354, 101.0250	7309	BS	Others
31	Pinellic acid	$C_{18}H_{34}O_5$	17.43	[M-H]-	329.2328	329.2344	4.9	229.1452, 211.1341, 183.1406, 171.1034, 139.1137	7204	GZ	Fatty acids and derivatives

Continued Table S1 Tentative annotation of chemical constituents in GZFL extract based on UPLC/Q-TOF-MS analysis

NO.	Compound	Molecular formula	t_R/min	Ion mode	$[M+H]^+/[M-H]^-$ Calculated *m/z*	Observed *m/z*	Error (10^{-6})	MS/MS (*m/z*)	Area	Attribution	Chemical class
32	1- (3,4 -dimethoxyphenyl) -1, 2- ethylene glycol -1-O-β-D-glucoside	$C_{16}H_{24}O_9$	10.63	[M-H]-	359.1342	359.1333	-2.5	197.0140, 137.0228, 122.0338	7135	BS	Phenylpropanoid glycosides
33	Benzoyloxypaeo-niflorin	$C_{30}H_{32}O_{13}$	14.56	[M-H]-	599.1765	599.1797	5.3	477.1432, 137.0257, 121.0307	7127	BS, MDP	Terpenoid glycosides
34	Pelargonic acid	$C_9H_{18}O_2$	25.81	[2M-H]-	315.2535	315.2548	4.1	313.2376, 297.2452	7096	FL	Fatty acids and derivatives
35	Taxifolin	$C_{15}H_{12}O_7$	14.62	[M-H]-	303.0505	303.0506	0.3	285.0508, 241.2009, 199.0724, 153.0240, 125.0289, 123.0461	6928	BS, GZ	Flavonoids

Continued Table S1 Tentative annotation of chemical constituents in GZFL extract based on UPLC/Q-TOF-MS analysis

NO.	Compound	Molecular formula	t_R/min	Ion mode	$[M+H]^+/[M-H]^-$ Calculated *m/z*	Observed *m/z*	Error (10^{-6})	MS/MS (*m/z*)	Area	Attribution	Chemical class
36	Truxillic acid	$C_{18}H_{16}O_4$	18.73	[M-H]-	295.0970	295.0988	6.1	277.0971, 233.0960, 205.1041, 180.0642, 147.0462, 103.0553, 77.0406	6893	GZ	Phenylpropanoids
37	Naringenin-C-glucoside	$C_{21}H_{22}O_{10}$	13.02	[M-H]-	433.1135	433.1161	6.0	313.0586, 271.0615, 151.0046	6850	BS	Flavonoid glycosides
38	Resacetophenone	$C_8H_8O_3$	15.43	[M-H]-	151.0395	151.0411	10.6	135.0095, 109.0306, 65.0402	6550	MDP	Phenolic compounds
39	Anhydrocinnzeylanol	$C_{20}H_{30}O_6$	15.08	[M-H]-	365.1964	365.2006	11.5	347.0917, 329.1709, 285.1782, 218.1402, 215.1455, 167.1089, 149.0960, 123.0536, 98.0262	6448	GZ	Terpenoids

Continued Table S1 Tentative annotation of chemical constituents in GZFL extract based on UPLC/Q-TOF-MS analysis

NO.	Compound	Molecular formula	t_R/min	Ion mode	$[M+H]^+/[M-H]^-$ Calculated *m/z*	Observed *m/z*	Error (10^{-6})	MS/MS (*m/z*)	Area	Attribution	Chemical class
40	Apiopaeonoside	$C_{20}H_{28}O_{12}$	12.15	[M+HCOO]-	505.1557	505.1566	1.8	459.1526, 165.0572	6274	MDP	Phenolic glycosides
41	3,24-Dioxo-16a-hydroxylanosta-7,9(11)- dien-21-oic acid	$C_{30}H_{44}O_5$	26.20	[M-H]-	483.3111	483.3115	0.8	387.2596, 385.2453, 369.2505, 367.3382, 353.2364, 325.2430, 323.2647, 85.0687	6202	FL	Terpenoids
42	30-Norhederagenin	$C_{29}H_{44}O_4$	24.75	[M-H]-	455.3161	455.3179	4.0	/	6070	BS, MDP	Terpenoids
43	Isoleukotoxindiol	$C_{18}H_{34}O_4$	23.76	[M-H]-	313.2379	313.2398	6.1	295.2273, 195.1481, 183.1402, 129.0918, 113.0932, 99.0836	6010	TR	Fatty acids and derivatives
44	(Epi)Catechin glucopyranoside	$C_{21}H_{24}O_{11}$	3.40	[M-H]-	451.1240	451.1248	1.8	415.1430, 289.1070	5943	BS	Flavonoid glycosides

Continued Table S1 Tentative annotation of chemical constituents in GZFL extract based on UPLC/Q-TOF-MS analysis

NO.	Compound	Molecular formula	t_R/min	Ion mode	$[M+H]^+/[M-H]^-$ Calculated *m/z*	Observed *m/z*	Error (10^{-6})	MS/MS (*m/z*)	Area	Attribution	Chemical class
45	Naringenin	$C_{15}H_{12}O_5$	17.08	[M-H]-	271.0607	271.0617	3.7	253.0566, 225.0574, 151.0051, 125.0282	5875	BS	Flavonoids
46	Palbinone	$C_{22}H_{30}O_4$	19.44	[M-H]-	357.2066	357.2084	5.0	329.2139, 167.9990	5794	BS, MDP	Terpenoids
47	Protocatechuic acid	$C_7H_6O_4$	11.21	[M-H]-	153.0188	153.0192	2.6	109.0302, 108.0214, 91.0201, 81.0353, 65.0030, 53.0398	5722	GZ, BS, TR, MDP	Phenolic acids
48	1-O-Vanilloyl-beta-D-glucose	$C_{14}H_{18}O_9$	10.53	[M-H]-	329.0873	329.0871	-0.6	167.0368, 152.0115, 123.0455	5692	BS	Phenolic glycosides
49	Truxinic acid	$C_{18}H_{16}O_4$	19.26	[M-H]-	295.0970	295.0991	7.1	277.0870, 233.0974, 147.0459	5679	GZ	Phenylpropanoids
50	Digalloylglucopy-ranose	$C_{13}H_{24}O_{19}$	10.36	[M-H]-	483.0834	483.0803	-6.4	313.0579, 169.0125	5546	BS	Hydrolyzable tannins

Continued Table S1 Tentative annotation of chemical constituents in GZFL extract based on UPLC/Q-TOF-MS analysis

NO.	Compound	Molecular formula	t_R/min	Ion mode	$[M+H]^+/[M-H]^-$ Calculated *m/z*	Observed *m/z*	Error (10^{-6})	MS/MS (*m/z*)	Area	Attribution	Chemical class
51	Luteolin	$C_{15}H_{10}O_6$	2.42	[M+H]+	287.0556	287.0557	0.3	240.9177, 212.8582, 156.9679	5538	GZ, TR	Flavonoids
52	Poricoic acid H	$C_{31}H_{48}O_5$	25.40	[M-H]-	499.3424	499.3413	-2.2	345.2297, 230.0280, 154.9927	5421	FL	Terpenoids
53	7-O-Methylmangiferin	$C_{20}H_{20}O_{11}$	13.66	[M-H]-	435.0927	435.0950	5.3	273.0614, 211.0251, 169.0162, 163.0421, 123.0109, 124.0155	5339	GZ	Phenolic glycosides
54	Ellagic acid-4-0-glucoside	$C_{20}H_{16}O_{13}$	11.81	[M-H]-	463.0513	463.0547	7.3	301.0024, 299.9921	5283	BS	Phenolic glycosides
55	Mudanpioside G or F	$C_{16}H_{24}O_8$	11.15	[M+HCOO]-	389.1448	389.1472	6.2	343.1400, 181.0887, 151.0763	5129	BS, MDP	Terpenoid glycosides
56	Cinncassiol A	$C_{20}H_{30}O_7$	13.05	[M-H]-	381.1913	381.1928	3.9	333.1695, 271.1677, 167.1104	5119	GZ	Terpenoids

Continued Table S1 Tentative annotation of chemical constituents in GZFL extract based on UPLC/Q-TOF-MS analysis

NO.	Compound	Molecular formula	t_R/min	Ion mode	$[M+H]^+/[M-H]^-$ Calculated *m/z*	Observed *m/z*	Error (10^{-6})	MS/MS (*m/z*)	Area	Attribution	Chemical class
57	Erucylamide	$C_{22}H_{43}NO$	21.8	[M+H]+	338.3423	338.3419	-1.2	321.3160, 303.3041, 296.3306	5109	BS	Fatty acids and derivatives
58	4-O-Methyldebenzoyl-paeoniflorin	$C_{17}H_{26}O_{10}$	10.50	[M-H]-	389.1448	389.1461	3.3	/	4966	BS, MDP	Terpenoid glycosides
59	4-Hydroxyphenylacetic acid	$C_8H_8O_3$	15.35	[M-H]-	151.0395	151.0402	4.6	135.0091, 107.0490, 93.0355, 59.7966	4833	GZ	Phenolic acids
60	Poricoic acid C	$C_{31}H_{44}O_4$	23.19	[M+H]+	481.3318	481.3329	2.3	325.2155	4817	FL	Terpenoids
61	Dehydrotumulosic acid	$C_{31}H_{48}O_4$	26.85	[M-H]-	483.3474	483.3461	-2.7	449.2675, 439.3349, 437.3388, 421.3075, 419.2910, 405.2481, 403.2550, 311.2066	4733	FL	Terpenoids
62	Gentisoyl glucoside	$C_{13}H_{16}O_9$	10.70	[M-H]-	315.0716	315.0721	1.6	153.0209	4598	BS, GZ	Phenolic glycosides

Continued Table S1 Tentative annotation of chemical constituents in GZFL extract based on UPLC/Q-TOF-MS analysis

NO.	Compound	Molecular formula	t_R/min	Ion mode	$[M+H]^+/[M-H]^-$ Calculated *m/z*	Observed *m/z*	Error (10^{-6})	MS/MS (*m/z*)	Area	Attribution	Chemical class
63	Vanillin	$C_8H_8O_3$	15.52	[M-H]-	151.0395	151.0417	14.6	136.0153, 123.0347, 121.0279, 91.0200	4506	GZ	Phenolic compounds
64	1'-O-Benzoylsucrose or sucrose 6-benzoate	$C_{19}H_{26}O_{12}$	11.36	[M+HCOO]-	491.1401	491.1425	4.9	323.0995, 121.0269	4404	MDP	Other glycosides
65	Paeoniflorin sulfonate	$C_{23}H_{28}O_{13}S$	11.52	[M-H]-	543.1172	543.1195	4.2	497.1565, 421.0826, 375.0779, 259.0272, 213.0235, 121.0298	4317	BS, MDP	Terpenoid glycosides
66	Quercetin 3-(2-glucosylrhamno-side) or Rutin	$C_{27}H_{30}O_{16}$	13.38	[M-H]-	609.1456	609.1449	-1.1	445.1093, 325.0727, 301.0505, 300.0057, 162.0279	4312	GZ, BS	Flavonoid glycosides

Continued Table S1 Tentative annotation of chemical constituents in GZFL extract based on UPLC/Q-TOF-MS analysis

NO.	Compound	Molecular formula	t_R/min	Ion mode	$[M+H]^+/[M-H]^-$		Error (10^{-6})	MS/MS (*m/z*)	Area	Attribution	Chemical class
					Calculated *m/z*	Observed *m/z*					
67	Oxypaeoniflorin	$C_{23}H_{28}O_{12}$	11.65	[M-H]-	495.1503	495.1521	3.6	465.1422, 333.0983, 177.0558, 137.0252, 93.0352	4297	BS, MDP	Terpenoid glycosides
68	Mudanpioside J	$C_{31}H_{34}O_{14}$	14.55	[M-H]-	629.1870	629.1910	6.4	477.1397, 167.0376, 165.0557, 137.0191, 121.0283	4287	BS, MDP	Terpenoid glycosides
69	Mudanpioside H	$C_{30}H_{32}O_{14}$	13.34	[M-H]-	615.1714	615.1746	5.2	431.1336, 137.0253, 93.0989	4215	MDP	Terpenoid glycosides
70	Methyl salicylate	$C_8H_8O_3$	15.37	[M-H]-	151.0395	151.0377	-11.9	136.0078, 121.0643, 93.0003	4210	BS	Phenolic compounds
71	3′-Methoxypuerarin	$C_{22}H_{22}O_{10}$	13.54	[M-H]-	445.1135	445.1177	9.4	297.0896	4179	FL	Flavonoid glycosides

Continued Table S1 Tentative annotation of chemical constituents in GZFL extract based on UPLC/Q-TOF-MS analysis

NO.	Compound	Molecular formula	t_R/min	Ion mode	$[M+H]^+/[M-H]^-$ Calculated *m/z*	Observed *m/z*	Error (10^{-6})	MS/MS (*m/z*)	Area	Attribution	Chemical class
72	Mudanpioside D	$C_{24}H_{30}O_{12}$	12.99	[M+HCOO]-	555.1714	555.1717	0.5	509.1679, 479.1566, 461.1448, 327.1109, 165.0559	4099	MDP	Terpenoid glycosides
73	23-Hydroxybetulinic acid	$C_{30}H_{48}O_4$	26.40	[M-H]-	471.3474	471.3492	3.8	/	4050	BS	Terpenoids
74	Kaempferol-7-O-β-D-glucoside	$C_{21}H_{20}O_{11}$	13.88	[M-H]-	447.0927	447.0962	7.8	285.0425, 265.1267, 130.0545	4038	BS	Flavonoid glycosides
75	Mudanpioside A	$C_{31}H_{34}O_{13}$	16.13	[M+HCOO]-	659.1976	659.2025	7.4	461.1460, 165.0568, 151.0430, 121.0304	4033	MDP	Terpenoid glycosides
76	Geraniol primeveroside	$C_{21}H_{36}O_{10}$	14.78	[M-H]-	447.2230	447.2248	4.0	149.0461, 89.0240, 59.0133	4009	BS	Terpenoid glycosides
77	Mandelic acid amide-β-D-glucopyranoside	$C_{14}H_{19}NO_7$	10.67	[M+HCOO]-	358.1138	358.1134	-1.1	161.0449, 150.0566, 113.0244, 101.0213, 71.0148	3893	TR	Other glycosides

Continued Table S1 Tentative annotation of chemical constituents in GZFL extract based on UPLC/Q-TOF-MS analysis

NO.	Compound	Molecular formula	t_R/min	Ion mode	$[M+H]^+/[M-H]^-$ Calculated *m/z*	Observed *m/z*	Error (10^{-6})	MS/MS (*m/z*)	Area	Attribution	Chemical class
78	25-Hydroxyporicoic acid C	$C_{31}H_{46}O_5$	23.19	[M-H]-	497.3267	497.3261	-1.2	481.0456, 467.3372, 423.2475, 421.2964, 407.2678, 373.2555, 339.1962, 253.1427	3893	FL	Terpenoids
79	16α,25-Dihydroxydehy-droeburiconic acid	$C_{31}H_{46}O_5$	24.59	[M-H]-	497.3267	497.3268	0.2	479.2855, 435.2441, 419.3111, 403.2635	3857	FL	Terpenoids
80	Desbenzoylpaeoniflorin	$C_{16}H_{24}O_{10}$	4.56	[M+HCOO]-	421.1346	421.1378	7.6	375.1293, 345.1205, 213.0751, 195.0682, 165.0565	3791	BS, MDP	Terpenoid glycosides
81	16α-Hydroxydehydro-trametenonic acid	$C_{30}H_{44}O_4$	25.66	[M-H]-	467.3161	467.3179	3.9	449.3309, 423.3050, 421.3299	3791	FL	Terpenoids
82	Ethyl gallate	$C_9H_{10}O_5$	13.68	[M-H]-	197.0450	197.0470	10.1	179.0528, 169.0155	3755	All	Phenolic compounds

Continued Table S1 Tentative annotation of chemical constituents in GZFL extract based on UPLC/Q-TOF-MS analysis

NO.	Compound	Molecular formula	t_R/min	Ion mode	$[M+H]^+/[M-H]^-$ Calculated *m/z*	Observed *m/z*	Error (10^{-6})	MS/MS (*m/z*)	Area	Attribution	Chemical class
83	3-(2-Hydroxyphenyl)-Propanoic acid	$C_9H_{10}O_3$	16.58	[M-H]-	165.0552	165.0566	8.5	147.0514, 121.0301	3704	GZ	Phenylpropanoids
84	Mudanpioside B	$C_{31}H_{34}O_{14}$	15.92	[M-H]-	629.1870	629.1908	6.0	535.1616, 431.1362, 165.0555, 151.0448, 137.0223	3692	BS, MDP	Terpenoid glycosides
85	Benzyl-β-D-glucopyranosyl (1-6)-β-D-glucopyranoside	$C_{19}H_{28}O_{11}$	11.55	[M+HCOO]-	477.1608	477.1629	4.4	323.0713, 269.1044, 179.0551, 161.0458	3678	GZ, TR	Other glycosides
86	1-O-β-D-Glucopyranosyl-paeonisuffrone	$C_{16}H_{24}O_9$	8.19	[M-H]-	359.1342	359.1362	5.6	179.0723, 116.0623, 101.0243	3591	BS, GZ	Terpenoid glycosides
87	Corilagin or Strictinin	$C_{27}H_{22}O_{18}$	11.00	[M-H]-	633.0728	633.0736	1.3	481.0614, 300.9995	3566	BS	Hydrolyzable tannins
88	Kaempferitrin	$C_{27}H_{30}O_{14}$	12.38	[M+HCOO]-	623.1612	623.1625	2.1	315.0455, 299.2001	3546	BS	Flavonoid glycosides

Continued Table S1 Tentative annotation of chemical constituents in GZFL extract based on UPLC/Q-TOF-MS analysis

NO.	Compound	Molecular formula	t_R/min	Ion mode	$[M+H]^+/[M-H]^-$ Calculated *m/z*	Observed *m/z*	Error (10^{-6})	MS/MS (*m/z*)	Area	Attribution	Chemical class
89	Cinnamomulactone	$C_{17}H_{16}O_5$	15.98	[M-H]-	299.0920	299.0951	10.4	255.1044, 240.0797, 239.0727, 223.0779, 123.0468, 105.0335	3540	GZ	Lignans
90	Syringic acid	$C_9H_{10}O_5$	13.54	[M-H]-	197.0450	197.0476	13.2	169.0161, 125.0253	3494	BS	Phenolic acids
91	Diglucosyl gallic acid	$C_{19}H_{26}O_{15}$	4.91	[M-H]-	493.1194	493.1200	1.2	331.0671, 313.0566, 271.0433, 211.0263, 169.0157, 125.0242, 124.0169, 87.0060	3337	BS	Phenolic glycosides
92	Benzoylalbiflorin	$C_{30}H_{32}O_{12}$	14.35	[M+HCOO]-	629.1870	629.1848	-3.5	583.1526, 121.0286	3329	BS	Terpenoid glycosides
93	Mudanpioside C	$C_{30}H_{32}O_{13}$	14.86	[M-H]-	599.1765	599.1792	4.5	299.0845, 165.0556, 137.0250, 121.0295	3249	BS, MDP	Terpenoid glycosides

Continued Table S1 Tentative annotation of chemical constituents in GZFL extract based on UPLC/Q-TOF-MS analysis

NO.	Compound	Molecular formula	t_R/min	Ion mode	$[M+H]^+/[M-H]^-$ Calculated *m/z*	Observed *m/z*	Error (10^{-6})	MS/MS (*m/z*)	Area	Attribution	Chemical class
94	Hederagenin	$C_{30}H_{48}O_4$	26.37	[M-H]-	471.3474	471.3485	2.3	/	3212	BS, MDP	Terpenoids
95	3β,16α-Dihydroxylanosta-7,9(11),24-trien-21-oic acid	$C_{30}H_{46}O_4$	26.09	[M-H]-	469.3318	469.3333	3.2	451.2700, 407.3274	3188	FL	Terpenoids
96	Triphyllic acid	$C_{30}H_{46}O_5$	25.81	[M-H]-	485.3267	485.3299	6.6	441.3371, 303.2473	3184	BS	Terpenoids
97	Galloyl、paeoniflorin	$C_{30}H_{32}O_{15}$	13.80	[M-H]-	631.1663	631.1672	1.4	465.1434, 313.0580, 271.0462	3167	BS, MDP	Terpenoid glycosides
98	Albiflorin	$C_{23}H_{28}O_{11}$	12.70	[M+HCOO]-	525.1608	525.1607	-0.2	479.1556, 177.0562, 121.0300, 77.0402	3065	MDP	Terpenoid glycosides
99	Paeonidanin E	$C_{46}H_{54}O_{21}$	12.75	[M-H]-	941.3079	941.3134	5.8	479.1581, 449.1473, 431.1479, 327.1102, 165.0599, 121.0288	3010	BS	Terpenoid glycosides

Continued Table S1 Tentative annotation of chemical constituents in GZFL extract based on UPLC/Q-TOF-MS analysis

NO.	Compound	Molecular formula	t_R/min	Ion mode	$[M+H]^+/[M-H]^-$		Error (10^{-6})	MS/MS (*m/z*)	Area	Attribution	Chemical class
					Calculated *m/z*	Observed *m/z*					
100	Ellagic acid	$C_{14}H_6O_8$	13.17	[M-H]-	300.9984	301.0003	6.3	283.9976, 257.0103, 229.0153, 201.0200	2912	BS	Phenolic compounds
101	16α，27-Dihydroxydehydrotrametenoic acid	$C_{30}H_{46}O_5$	22.50	[M-H]-	485.3267	485.3276	1.9	/	2902	FL	Terpenoids
102	Mandelic acid amide-β-gentiobioside	$C_{20}H_{29}NO_{12}$	10.45	[M+HCOO]-	520.1666	520.1688	4.2	431.1633, 312.1102, 263.0780, 245.0661, 221.0678, 179.0566, 161.0458, 150.0562, 119.0351, 101.0244, 89.0243, 71.0139, 59.0139	2861	TR	Other glycosides
103	Penstemide	$C_{21}H_{32}O_{10}$	11.35	[M-H]-	443.1917	443.1948	7.0	101.0224	2820	BS	Terpenoid glycosides

Continued Table S1 Tentative annotation of chemical constituents in GZFL extract based on UPLC/Q-TOF-MS analysis

NO.	Compound	Molecular formula	t_R/min	Ion mode	$[M+H]^+/[M-H]^-$ Calculated *m/z*	Observed *m/z*	Error (10^{-6})	MS/MS (*m/z*)	Area	Attribution	Chemical class
104	Galloylglucose	$C_{13}H_{16}O_{10}$	3.64	[M-H]-	331.0665	331.0695	9.1	271.0267, 235.0499, 169.0147, 125.0246	2814	BS, MDP	Phenolic glycosides
105	Poriacosone B	$C_{30}H_{46}O_5$	25.36	[M-H]-	485.3267	485.3277	2.1	467.3273, 441.3178, 409.2923, 397.2740, 371.2548, 353.2171	2784	FL	Terpenoids
106	Pinicolic acid E	$C_{30}H_{46}O_4$	26.10	[M-H]-	469.3318	469.3320	0.4	423.3232, 409.3072, 391.2983, 379.1714, 367.1935	2743	FL	Terpenoids
107	Galloylsucrose	$C_{19}H_{26}O_{15}$	8.78	[M-H]-	493.1194	493.1211	3.4	331.0689, 179.0650, 169.0150, 163.0557 9, 125.0245	2687	BS, MDP	Phenolic glycosides
108	Anhydrocinnzeylanine	$C_{22}H_{32}O_7$	19.13	[M-H]-	407.2070	407.2104	8.3	347.1871	2681	GZ	Terpenoids

Continued Table S1 Tentative annotation of chemical constituents in GZFL extract based on UPLC/Q-TOF-MS analysis

NO.	Compound	Molecular formula	t_R/min	Ion mode	$[M+H]^+/[M-H]^-$ Calculated *m/z*	Observed *m/z*	Error (10^{-6})	MS/MS (*m/z*)	Area	Attribution	Chemical class
109	Galloylpaeoniflorin sulfonate	$C_{30}H_{32}O_{17}S$	12.40	[M-H]-	695.1282	695.1286	0.6	/	2592	BS, MDP	Terpenoid glycosides
110	(S)-Mandelic acid O-β-D-glucopyranoside	$C_{14}H_{18}O_8$	11.29	[M-H]-	313.0923	313.0948	8.0	269.0998, 161.0471	2591	TR	Other glycosides
111	Secoisolariciresinol	$C_{20}H_{26}O_6$	14.52	[M-H]-	361.1651	361.1654	0.8	346.1388, 179.0643, 165.0592, 122.0394	2503	GZ	Lignans
112	Balanophonin	$C_{20}H_{20}O_6$	19.35	[M-H]-	355.1182	355.1208	7.3	337.0978, 305.0959	2415	GZ	Lignans
113	Mono-galloyl-glucoside	$C_{13}H_{16}O_{10}$	5.56	[M-H]-	331.0665	331.0673	2.4	271.0479, 211.0252, 169.0140	2329	BS	Phenolic glycosides
114	Amygdalin	$C_{20}H_{27}NO_{11}$	11.88	[M-H]-	456.1506	456.1537	6.8	323.1010, 263.0780, 245.0680, 179.0565, 161.0478	2329	TR	Cyanogenic glycosides
115	Isomaltopaeoniflorin sulfonate	$C_{29}H_{38}O_{18}S$	11.25	[M-H]-	705.1701	705.1768	9.5	543.0778, 421.0621, 259.0273	2299	BS	Terpenoid glycosides

Continued Table S1 Tentative annotation of chemical constituents in GZFL extract based on UPLC/Q-TOF-MS analysis

NO.	Compound	Molecular formula	t_R/min	Ion mode	$[M+H]^+/[M-H]^-$ Calculated *m/z*	Observed *m/z*	Error (10^{-6})	MS/MS (*m/z*)	Area	Attribution	Chemical class
116	Mandelic acid gentiobioside	$C_{20}H_{28}O_{13}$	10.99	[M-H]-	475.1452	475.1472	4.2	431.1557	2268	TR	Other glycosides
117	Kaempferol-3-O-β-D-glucoside	$C_{21}H_{20}O_{11}$	13.54	[M-H]-	447.0927	447.0913	-3.1	285.0402, 130.0271, 109.0281	2253	BS	Flavonoid glycosides
118	Maltol	$C_6H_6O_3$	7.52	[M+H]+	127.0395	127.0387	-6.3	109.0287, 81.0333	2248	BS	Others
119	Galloylquinic acid	$C_{14}H_{16}O_{10}$	5.02	[M-H]-	343.0665	343.0684	5.5	191.0569, 169.0147	2227	BS	Phenolic acids
120	(2R)-[(6-O-β-D-glucopyranosyl-β-D-glucopyra-nosyl)oxy](phenyl)ethanoic acid	$C_{20}H_{28}O_{13}$	11.75	[M-H]-	475.1452	475.1458	1.3	431.2131, 269.1058, 179.0690, 161.0487, 143.0551	2129	TR	Other glycosides
121	β-Pinen-10-yl-β-vicianoside	$C_{21}H_{34}O_{10}$	14.84	[M-H]-	445.2074	445.2094	4.5	293.0883	1844	BS	Terpenoid glycosides
122	Isoquercitrin	$C_{21}H_{20}O_{12}$	12.95	[M-H]-	463.0877	463.0920	9.3	301.0358, 271.0259, 255.0353, 243.0371	1839	BS	Flavonoid glycosides

Continued Table S1 Tentative annotation of chemical constituents in GZFL extract based on UPLC/Q-TOF-MS analysis

NO.	Compound	Molecular formula	t_R/min	Ion mode	$[M+H]^+/[M-H]^-$ Calculated *m/z*	$[M+H]^+/[M-H]^-$ Observed *m/z*	Error (10^{-6})	MS/MS (*m/z*)	Area	Attribution	Chemical class
123	Desbenzoylalbiflorin	$C_{16}H_{24}O_{10}$	4.89	[M+HCOO]-	421.1346	421.1347	0.2	375.1302, 345.1199	1822	BS	Terpenoid glycosides
124	Quercetin 3-O-alpha-L-rhamnopyranosyl-(1->2)-alpha-L-arabinopyranoside	$C_{26}H_{28}O_{15}$	13.18	[M-H]-	579.1350	579.1382	5.5	417.2507, 300.0280, 245.0820, 203.0256	1813	GZ	Flavonoid glycosides
125	Isosyringinoside or its isomer	$C_{23}H_{34}O_{14}$	3.36	[M+HCOO]-	579.1925	579.1970	7.8	371.0942, 179.0614, 161.0419, 119.0892, 89.0264	1716	GZ	Phenylpropanoid glycosides
126	O-β-D-Gentiobiosyl-D-(-)-mandelamide	$C_{20}H_{29}NO_{12}$	10.46	[M-H]-	474.1612	474.1607	-1.1	323.0994, 312.1091, 179.0570, 161.0468, 150.0556	1665	TR	Other glycosides
127	Galloyl desbenzoylpaeoniflorin	$C_{23}H_{28}O_{14}$	10.94	[M-H]-	527.1401	527.1423	4.2	497.1322, 169.0146, 151.0018	1645	BS, MDP	Terpenoid glycosides
128	Protocatechualdehyde	$C_7H_6O_3$	12.28	[M-H]-	137.0239	137.0257	13.1	109.0302	1594	GZ, BS	Phenolic compounds

Continued Table S1 Tentative annotation of chemical constituents in GZFL extract based on UPLC/Q-TOF-MS analysis

NO.	Compound	Molecular formula	t_R/min	Ion mode	$[M+H]^+/[M-H]^-$		Error (10^{-6})	MS/MS (*m/z*)	Area	Attribution	Chemical class
					Calculated *m/z*	Observed *m/z*					
129	Polystachyol	$C_{22}H_{28}O_8$	13.39	[M-H]-	419.1706	419.1721	3.6	404.1495, 389.1225, 373.1326, 359.1143	1579	GZ	Lignans
130	Aconitic acid	$C_6H_6O_6$	3.95	[M-H]-	173.0086	173.0108	12.7	129.0624, 111.0036, 85.0256	1471	BS	Organic acids
131	9,12,13,TriHODE	$C_{18}H_{32}O_5$	16.54	[M-H]-	327.2172	327.2193	6.4	291.2064, 239.1197, 229.1457, 211.1362, 183.1487, 171.1035	1422	GZ	Fatty acids and derivatives
132	Gallic acid	$C_7H_6O_5$	7.42	[M-H]-	169.0137	169.0149	7.1	125.0249, 123.0092	1410	BS, MDP	Phenolic acids
133	Pyrogallol	$C_6H_6O_3$	7.36	[M-H]-	125.0239	125.0249	8.0	107.0241, 95.0181, 81.0343	1339	BS	Phenolic compounds
134	Cinnamylalcohol-6-O-α-furanara-binose-O-β-glucopyranoside	$C_{20}H_{28}O_{10}$	10.78	[M-H]-	427.1604	427.1562	-9.8	293.0364, 161.0413	1048	GZ	Phenylpropanoid glycosides

Continued Table S1 Tentative annotation of chemical constituents in GZFL extract based on UPLC/Q-TOF-MS analysis

NO.	Compound	Molecular formula	t_R/min	Ion mode	$[M+H]^+/[M-H]^-$ Calculated *m/z*	Observed *m/z*	Error (10^{-6})	MS/MS (*m/z*)	Area	Attribution	Chemical class
135	Cinncassiol A 19-O-β-D-glucopyranoside	$C_{26}H_{40}O_{12}$	11.53	[M-H]-	543.2442	543.2513	13.1	363.0459, 167.0891	986	GZ	Terpenoid glycosides
136	5-Hydroxymethylfurfural	$C_6H_6O_3$	7.35	[M-H]-	125.0239	125.0242	2.4	108.0230, 79.0190	521	BS, MDP	Others
137	2-Hydroxy-cinnamaldehyde	$C_9H_8O_2$	32.06	[M+H]+	149.0603	149.0592	-7.4	121.0283, 65.0386	ND	GZ	Phenylpropanoids
138	R-mandelamide	$C_8H_9NO_2$	10.44	[M+H]+	152.0712	152.0714	1.3	134.0237, 106.0649	ND	TR	Others
139	3-Hydroxy-4-methoxyaceto-phenone	$C_9H_{10}O_3$	12.51	[M+H]+	167.0708	167.0717	5.4	149.0610, 125.0603, 118.0406	ND	BS, MDP	Phenolic compounds
140	Cassiferaldehyde	$C_{10}H_{10}O_3$	12.71	[M+H]+	179.0708	179.0700	-4.5	161.0605, 151.0752, 133.0652, 123.0803, 105.0699	ND	GZ	Phenylpropanoids
141	Paeonilactone B	$C_{10}H_{12}O_4$	5.00	[M+H]+	197.0814	197.0815	0.5	151.0755, 133.0636	ND	BS, MDP	Terpenoids

Continued Table S1 Tentative annotation of chemical constituents in GZFL extract based on UPLC/Q-TOF-MS analysis

NO.	Compound	Molecular formula	t_R/min	Ion mode	$[M+H]^+/[M-H]^-$ Calculated *m/z*	Observed *m/z*	Error (10^{-6})	MS/MS (*m/z*)	Area	Attribution	Chemical class
142	2-Methoxycinnamic acid	$C_{10}H_{10}O_3$	12.55	[M+H+H₂O]+	197.0814	197.0829	7.6	179.0694, 151.0396, 107.0485, 77.0381	ND	GZ	Phenylpropanoids
143	Paeonilactone D	$C_{10}H_{16}O_4$	2.43	[M+H]+	201.1126	201.1130	2.0	99.9350, 81.0359	ND	BS	Terpenoids
144	Sydowic acid	$C_{15}H_{20}O_4$	15.76	[M-H]-	263.1283	263.1291	3.0	219.1414, 204.1176, 189.0935, 153.0924, 136.0533, 122.0383, 92.0262	ND	GZ	Terpenoids
145	Prenyl glucoside	$C_{11}H_{20}O_6$	2.76	$[M+NH_4]+$	266.1604	266.1607	1.1	163.0502, 145.0506, 127.0390, 85.0283, 69.0333	ND	GZ	Terpenoid glycosides
146	Quercetin	$C_{15}H_{10}O_7$	15.66	[M-H]-	301.0348	301.0351	1.0	273.0415, 178.9996, 151.0042	ND	GZ, BS, MDP	Flavonoids

Continued Table S1 Tentative annotation of chemical constituents in GZFL extract based on UPLC/Q-TOF-MS analysis

NO.	Compound	Molecular formula	t_R/min	Ion mode	$[M+H]^+/[M-H]^-$ Calculated *m/z*	Observed *m/z*	Error (10^{-6})	MS/MS (*m/z*)	Area	Attribution	Chemical class
147	Paeoniflorigenone	$C_{17}H_{18}O_6$	12.55	[M+H]+	319.1182	319.1187	1.6	301.1063, 249.0550, 197.0812, 186.0945	ND	BS, MDP	Terpenoids
148	N-Methylhernagine	$C_{20}H_{23}NO_4$	12.12	[M+H]+	342.1705	342.1720	4.4	287.1138, 265.0877, 237.0920	ND	BS	Alkaloids
149	6-O-β-D-Glucopyranosyl lactinolide	$C_{16}H_{26}O_9$	2.98	[M+H]+	363.1655	363.1672	4.7	381.0822, 283.1414, 116.0669	ND	GZ	Terpenoid glycosides
150	Prupersin F	$C_{21}H_{23}NO_8$	14.28	[M-H]-	416.1345	416.1364	4.6	/	ND	TR	Other glycosides
151	Isosalipurposide	$C_{21}H_{22}O_{10}$	12.85	[M-H]-	433.1135	433.1160	5.8	271.0621, 151.0043, 119.0533	ND	BS	Flavonoid glycosides
152	Luteoloside	$C_{21}H_{20}O_{11}$	3.35	[M-H]-	447.0927	447.0886	-9.2	373.1454	ND	GZ	Flavonoid glycosides
153	Quercitrin	$C_{21}H_{20}O_{11}$	13.56	[M-H]-	447.0927	447.0935	1.8	301.0203	ND	GZ	Flavonoid glycosides
154	Astragalin	$C_{21}H_{20}O_{11}$	14.78	[M-H]-	447.0927	447.0938	2.5	197.0325, 150.9952	ND	BS	Flavonoid glycosides

Continued Table S1 Tentative annotation of chemical constituents in GZFL extract based on UPLC/Q-TOF-MS analysis

NO.	Compound	Molecular formula	t_R/min	Ion mode	$[M+H]^+/[M-H]^-$ Calculated *m/z*	Observed *m/z*	Error (10^{-6})	MS/MS (*m/z*)	Area	Attribution	Chemical class
155	16α-Hydroxy-lanosta-8, 24 (31)-dien-21-oic-acid	$C_{30}H_{46}O_3$	26.37	[M+H]+	455.3525	455.3531	1.3	313.2556	ND	FL	Terpenoids
156	Dehydrotrametenolic acid	$C_{30}H_{46}O_3$	26.44	[M+H]+	455.3525	455.3537	2.6	437.3407, 313.2549, 295.2433	ND	FL	Terpenoids
157	Alisol I 23-acetate	$C_{30}H_{46}O_3$	23.73	[M+H]+	455.3525	455.3538	2.9	437.3420, 419.3354, 341.3022	ND	FL	Terpenoids
158	Mudanoside B	$C_{18}H_{24}O_{14}$	9.96	[M-H]-	463.1088	463.1098	2.2	301.0586, 169.0155	ND	MDP	Phenolic glycosides
159	3-Oxo-16α-hydroxylanosta-7,9(11),24-trien-21-oic acid	$C_{30}H_{44}O_4$	25.70	[M-H]-	467.3161	467.3163	0.4	389.2898	ND	FL	Terpenoids
160	3,4-Seco-lanosta-4(28),7,9(11),24-tetraen-3,21-dioic acid	$C_{30}H_{42}O_4$	26.20	[M+H]+	467.3161	467.3188	5.8	325.2184	ND	FL	Terpenoids
161	Digalloylglucose	$C_{20}H_{20}O_{14}$	11.24	[M-H]-	483.0775	483.0793	3.7	/	ND	BS, MDP	Hydrolyzable tannins

Continued Table S1 Tentative annotation of chemical constituents in GZFL extract based on UPLC/Q-TOF-MS analysis

NO.	Compound	Molecular formula	t_R/min	Ion mode	$[M+H]^+/[M-H]^-$ Calculated *m/z*	Observed *m/z*	Error (10^{-6})	MS/MS (*m/z*)	Area	Attribution	Chemical class
162	6,16α-Dihydroxydehydrotrametenonic acid	$C_{30}H_{44}O_5$	22.50	[M+H]+	485.3267	485.3276	1.9	439.1517, 437.3265, 419.2722, 389.2600, 325.2616	ND	FL	Terpenoids
163	3β,15α-Dihydroxy-24-oxolanosta-8-en-21-oic acid	$C_{30}H_{48}O_5$	21.66	[M-H]-	487.3424	487.3434	2.1	443.2177, 385.3013	ND	FL	Terpenoids
164	Daedaleanic acid B	$C_{30}H_{48}O_5$	21.63	[M-H]-	487.3424	487.3430	1.2	469.3345, 443.3255, 441.3081, 427.3191, 409.3377	ND	FL	Terpenoids
165	3α,16α,25-Trihydroxylanosta-8,24-dien-21-oic acid	$C_{30}H_{48}O_5$	22.91	[M-H]-	487.3424	487.3415	-1.9	441.3271, 389.2620, 371.2634, 355.2500	ND	FL	Terpenoids
166	4"-Hydroxyl-albiflorin	$C_{23}H_{28}O_{12}$	11.67	[M+H]+	497.1659	497.1704	9.1	197.0806, 179.0706, 161.0598, 121.0281	ND	BS	Terpenoid glycosides

Continued Table S1 Tentative annotation of chemical constituents in GZFL extract based on UPLC/Q-TOF-MS analysis

NO.	Compound	Molecular formula	t_R/min	Ion mode	$[M+H]^+/[M-H]^-$ Calculated *m/z*	Observed *m/z*	Error (10^{-6})	MS/MS (*m/z*)	Area	Attribution	Chemical class
167	Poricoic acid E	$C_{30}H_{44}O_6$	19.30	[M-H]-	499.3060	499.3047	-2.6	427.2881, 425.2742, 387.2102, 369.2656	ND	FL	Terpenoids
168	Hydroxy-dehydrotumulosic acid	$C_{31}H_{48}O_5$	21.57	[M-H]-	499.3424	499.3433	1.8	345.1500, 297.2239	ND	FL	Terpenoids
169	16α,25(29)-Dihydroxyeburiconic acid	$C_{31}H_{48}O_5$	21.61	[M-H]-	499.3424	499.3441	3.4	329.2507, 171.0011	ND	FL	Terpenoids
170	1-O-β-D-Glucopyranosyl-8-O-benzoylpaeonisuffrone	$C_{23}H_{28}O_{10}$	14.10	[M+HCOO]-	509.1659	509.1696	7.3	359.1919, 121.0311	ND	BS	Terpenoid glycosides
171	Glochiflavanoside B	$C_{24}H_{30}O_{12}$	13.93	[M-H]-	509.1659	509.1690	6.1	463.2206, 341.1201, 283.0702, 197.0810, 121.0310, 77.0415	ND	BS	Flavonoid glycosides

Continued Table S1 Tentative annotation of chemical constituents in GZFL extract based on UPLC/Q-TOF-MS analysis

NO.	Compound	Molecular formula	t_R/min	Ion mode	$[M+H]^+/[M-H]^-$		Error (10^{-6})	MS/MS (*m/z*)	Area	Attribution	Chemical class
					Calculated *m/z*	Observed *m/z*					
172	3,4-Dicaffeoylquinic acid	$C_{25}H_{24}O_{12}$	3.52	[M-H]-	515.1190	515.1241	9.9	237.0454	ND	BS	Phenylpropanoids
173	Pachymic acid	$C_{33}H_{52}O_5$	31.15	[M-H]-	527.3737	527.3764	5.1	509.2900, 483.4079, 481.3459, 467.3694, 465.3321, 449.3451, 421.3618, 405.3257, 355.2342, 295.2318	ND	FL	Terpenoids
174	Oxypaeoniflorin sulfonate	$C_{23}H_{28}O_{14}S$	10.57	[M-H]-	559.1122	559.1131	1.6	421.0821, 259.0930, 213.0521, 137.0374	ND	BS, MDP	Terpenoid glycosides
175	Isoviolanthin	$C_{27}H_{30}O_{14}$	13.84	[M-H]-	577.1557	577.1557	0.0	457.1361	ND	BS	Flavonoid glycosides
176	Benzoylpaeoniflorin	$C_{30}H_{32}O_{12}$	17.69	[M-H]-	583.1816	583.1783	-5.7	121.0303	ND	MDP	Terpenoid glycosides

Continued Table S1 Tentative annotation of chemical constituents in GZFL extract based on UPLC/Q-TOF-MS analysis

NO.	Compound	Molecular formula	t_R/min	Ion mode	$[M+H]^+/[M-H]^-$ Calculated *m/z*	Observed *m/z*	Error (10^{-6})	MS/MS (*m/z*)	Area	Attribution	Chemical class
177	Mudanpioside E sulphonate	$C_{24}H_{30}O_{15}S$	10.83	[M-H]-	589.1227	589.1296	11.7	121.8468	ND	BS	Terpenoid glycosides
178	Tiliroside	$C_{30}H_{26}O_{13}$	12.1	[M-H]-	593.1295	593.1212	-14.0	285.0378	ND	GZ	Flavonoid glycosides
179	Quercetin-3-O-glucoside-7-O-rhamnoside	$C_{27}H_{30}O_{16}$	12.79	[M-H]-	609.1456	609.1497	6.7	447.1476, 300.0411, 126.0280	ND	GZ	Flavonoid glycosides
180	Kaempferol 3, 7-diglucoside	$C_{27}H_{30}O_{16}$	13.79	[M-H]-	609.1456	609.1491	5.8	283.0307	ND	BS, MDP	Flavonoid glycosides
181	Suffruticoside A	$C_{27}H_{32}O_{16}$	12.32	[M-H]-	611.1612	611.1632	3.3	445.0971, 283.3968, 165.0549	ND	MDP	Phenolic glycosides
182	Suffruticoside B	$C_{27}H_{32}O_{16}$	12.46	[M-H]-	611.1612	611.1648	5.9	445.1010, 169.0156, 165.0560	ND	MDP	Phenolic glycosides
183	Suffruticoside C	$C_{27}H_{32}O_{16}$	13.31	[M-H]-	611.1612	611.1641	4.8	445.1008, 343.0688, 283.0439, 169.0151	ND	MDP	Phenolic glycosides

Continued Table S1 Tentative annotation of chemical constituents in GZFL extract based on UPLC/Q-TOF-MS analysis

NO.	Compound	Molecular formula	t_R/min	Ion mode	$[M+H]^+/[M-H]^-$ Calculated *m/z*	Observed *m/z*	Error (10^{-6})	MS/MS (*m/z*)	Area	Attribution	Chemical class
184	Suffruticoside D	$C_{27}H_{32}O_{16}$	11.99	[M-H]-	611.1612	611.1623	1.8	445.1001, 283.0470, 169.0145	ND	MDP	Phenolic glycosides
185	Galloylalbiflorin	$C_{30}H_{32}O_{15}$	13.22	[M-H]-	631.1663	631.1690	4.3	313.0578, 169.0148, 125.0249	ND	BS, MDP	Terpenoid glycosides
186	Trigalloy glucose	$C_{27}H_{24}O_{18}$	11.78	[M-H]-	635.0884	635.0919	5.5	483.0817, 169.0156	ND	BS, MDP	Hydrolyzable tannins
187	4-O-Methylgalloylpaeoniflorin	$C_{31}H_{34}O_{15}$	14.55	[M-H]-	645.1820	645.1860	6.2	/	ND	BS, MDP	Terpenoid glycosides
188	Galloyloxypaeoniflorin	$C_{30}H_{32}O_{16}$	12.22	[M-H]-	647.1612	647.1643	4.8	211.0273, 169.0149, 125.0250	ND	MDP	Terpenoid glycosides
189	Suffruticoside E	$C_{26}H_{38}O_{17}$	11.74	[M+HCOO]-	667.2086	667.2092	0.9	621.2041, 455.1441, 165.0527	ND	MDP	Phenolic glycosides

Continued Table S1 Tentative annotation of chemical constituents in GZFL extract based on UPLC/Q-TOF-MS analysis

NO.	Compound	Molecular formula	t_R/min	Ion mode	$[M+H]^+/[M-H]^-$ Calculated *m/z*	Observed *m/z*	Error (10^{-6})	MS/MS (*m/z*)	Area	Attribution	Chemical class
190	Isomaltopaeoniflorin or 6-O-β-D-glucopyranosylalbiflorin	$C_{29}H_{38}O_{16}$	12.38	[M+HCOO]-	687.2136	687.2178	6.1	641.2118, 593.1889, 471.1519, 323.1019, 165.0568, 121.0308	ND	BS	Terpenoid glycosides
191	Paeonin D	$C_{37}H_{36}O_{16}$	17.28	[M-H]-	735.1925	735.1956	4.2	613.1577, 169.0146	ND	BS	Terpenoid glycosides
192	Digalloylpaeoniflorin	$C_{37}H_{36}O_{19}$	14.09	[M-H]-	783.1773	783.1810	4.7	631.1746, 465.0675, 313.0580, 125.0216	ND	BS	Terpenoid glycosides
193	Tetragalloyglucose	$C_{34}H_{28}O_{22}$	12.23	[M-H]-	787.0994	787.1040	5.8	635.0922, 617.0788, 313.0763, 169.0145	ND	BS, MDP	Hydrolyzable tannins
194	Paeoniflorin B	$C_{36}H_{42}O_{17}$	14.38	[M+HCOO]-	791.2399	791.2371	-3.5	745.2337	ND	MDP	Terpenoid glycosides

Continued Table S1 Tentative annotation of chemical constituents in GZFL extract based on UPLC/Q-TOF-MS analysis

NO.	Compound	Molecular formula	t_R/min	Ion mode	$[M+H]^+/[M-H]^-$ Calculated *m/z*	Observed *m/z*	Error (10^{-6})	MS/MS (*m/z*)	Area	Attribution	Chemical class
195	Diglucosylpaeoniflorin	$C_{35}H_{48}O_{21}$	12.15	[M-H]-	803.2610	803.2637	3.4	773.2556, 755.2401, 641.2314, 611.3270, 489.1731, 165.0574	ND	BS	Terpenoid glycosides
196	1,2,3,4,6-Pentagalloylglucose	$C_{41}H_{32}O_{26}$	12.77	[M-H]-	939.1103	939.1141	4.1	769.0943, 617.0819, 169.0152	ND	BS, MDP	Hydrolyzable tannins
197	Hexagalloyl glucose	$C_{48}H_{36}O_{30}$	13.3	[M-H]-	1091.1213	1091.1260	3.9	/	ND	BS, MDP	Hydrolyzable tannins

[1] GZ: *Ramulus Cinnamomi*; FL: *Poria*; TR: *Semen Persicae*; MDP: *Cortex Moutan*; BS: *Radix Paeoniae Alba.*

[2] ND: Not Detected.

Table S2 Common components in the component-target network of GZFL

	Ramulus Cinnamomi (GZ)	*Poria* (FL)	*Cortex Moutan* (MDP)	*Semen Persicae* (TR)	*Radix Paeoniae Alba* (BS)
A	+	-	+	-	+
B	+	-	-	-	+
C	+	-	-	+	-
D	+	-	+	-	+
E	+	-	+	+	+
F	+	+	+	+	+

[1] GZ: *Ramulus Cinnamomi*; FL: *Poria*; TR: *Semen Persicae*; MDP: *Cortex Moutan*; BS: *Radix Paeoniae Alba.*

[2] "+" indicates the presence of the component in the corresponding herb, whereas "–" indicates its absence.

Supplementary Note S1. MS/MS Fragmentation Analysis of Representative GZFL Constituents

Representative MS/MS fragmentation characteristics of selected GZFL constituents are described below to provide additional support for the tentative structural annotations summarized in Table S1.

1.1 Fragmentation Analysis of Phenolic Compounds

Phenolic compounds detected in the drug-containing serum of GZFL-treated rats were mainly derived from *Radix Paeoniae Alba* and *Cortex Moutan*. Taking paeonol as a representative example, its molecular ion was observed at *m/z* 165.0561 $[M-H]^-$ in negative ion mode. The molecular formula was assigned as $C_9H_{10}O_3$ based on the acquired MS data and literature information. The characteristic MS/MS fragment ion at m/z 150.0326 $[M-H-CH_3]^-$ was generated by the loss of a methyl group, while additional characteristic product ions were observed at m/z 122.0374 and 91.0194.

Vanillin was selected as another representative phenolic compound. Its molecular ion was observed at *m/z* 151.0417 $[M-H]^-$ in negative ion mode. The molecular formula was assigned as $C_8H_8O_3$ based on the acquired MS data and literature information. The characteristic MS/MS fragment ion at m/z 136.0153 $[M-H-CH_3]^-$ was generated by the loss of a methyl group, while additional characteristic product ions were observed at m/z 123.0347, 121.0279, and 91.0200.

1.2 Fragmentation Analysis of Glycosidic Compounds

Glycosidic compounds detected in the drug-containing serum of GZFL-treated rats included several structural subclasses and were mainly derived from *Radix Paeoniae Alba* and *Cortex Moutan*. Taking paeoniflorin as a representative example, its molecular ion was observed at m/z 481.1707 $[M+H]^+$ in positive ion mode. The molecular formula was assigned as $C_{23}H_{28}O_{11}$ based on the acquired MS data and literature information. The characteristic MS/MS fragment ions were observed at m/z 375.1271 and 123.0801.

1.3 Fragmentation Analysis of Phenylpropanoids

Phenylpropanoid constituents detected in the drug-containing serum of GZFL-treated rats were mainly derived from *Ramulus Cinnamomi*. Taking cinnamic acid as a representative example, its molecular ion was observed at *m/z* 167.0710 $[M+H+H_2O]^+$ in positive ion mode. The molecular formula was assigned as $C_9H_8O_2$ based on the acquired MS data and literature information. The fragment ion at m/z 149.0603 $[M+H]^+$ was generated by the loss of H_2O (18 Da) from the precursor ion. Further fragmentation yielded characteristic product ions at m/z 121.0653, 103.0539, 91.0543, and 77.0388.

1.4 Fragmentation Analysis of Steroids

Steroidal constituents detected in the drug-containing serum of GZFL-treated rats were mainly derived from *Radix Paeoniae Alba*, *Cortex Moutan*, and *Po*ria. Taking 3β,16α-dihydroxylanosta-7,9(11),24-trien-21-oic acid as a representative example, its

molecular ion was observed at *m/z* 469.3333 $[M-H]^-$ in negative ion mode. The molecular formula was assigned as $C_{30}H_{46}O_4$ based on the acquired MS data and literature information. The characteristic MS/MS fragment ions were observed at m/z 451.2700 and 407.3274.

1.5 Fragmentation Analysis of Terpenoid-Related Compounds

Terpenoid-related compounds detected in the drug-containing serum of GZFL-treated rats were mainly derived from *Radix Paeoniae Alba* and *Cortex Moutan*. Taking picrocrocinic acid as a representative example, its molecular ion was observed at *m/z* 345.1547 $[M-H]^-$ in negative ion mode. The molecular formula was assigned as $C_{16}H_{26}O_8$ based on the acquired MS data and literature information. The characteristic MS/MS fragment ions were observed at m/z 179.0563, 165.0943, and 59.0156.

1.6 Fragmentation Analysis of Flavonoid-Related Compounds

Flavonoid-related compounds detected in the drug-containing serum of GZFL-treated rats were mainly derived from *Radix Paeoniae Alba* and *Ramulus Cinnamomi*. Taking isoquercitrin as a representative example, its molecular ion was observed at *m/z* 463.0920 $[M-H]^-$ in negative ion mode. The molecular formula was assigned as $C_{21}H_{20}O_{12}$ based on the acquired MS data and literature information. The characteristic MS/MS fragment ion at m/z 301.0358 $[M-H-C_6H_{10}O_5]^-$ was generated by the loss of a hexose moiety (162 Da), corresponding to the deprotonated quercetin aglycone. Further fragmentation yielded the product ion at m/z 271.0259, while additional characteristic product ions were observed at m/z 255.0353 and 243.0371.

Supplementary Method S1. LC-MS/MS Conditions

1. LC-MS/MS Conditions for Compound Identification

1.1 Liquid Chromatography Conditions

Chromatographic separation was performed using an XSelect® HSS T3 column (3.5 μm, 4.6 × 150 mm) maintained at 40 °C. The mobile phase consisted of ultrapure water containing 0.1% formic acid (A) and acetonitrile (B), with a flow rate of 0.5 mL/min. The gradient elution program was as follows: 0–4.0 min, 5% B; 4.0–10.0 min, 5%–40% B; 10.0–20.0 min, 40%–65% B; 20.0–22.0 min, 65% B; 22.0–23.0 min, 65%–90% B; 23.0–28.0 min, 90% B; and 28.0–29.0 min, 5% B.

1.2 Mass Spectrometry Conditions

Analyses were performed using an AB SCIEX TripleTOF 5600 instrument equipped with an electrospray ionization (ESI) source operated in negative ion mode. The TOF MS scan range was m/z 50–1200 and the product ion scan range was m/z 50–1200. The source temperature was 550 °C and the ion spray voltage floating was −4,500 V.

2. LC-MS/MS Conditions for Pharmacometabolomics Analysis

2.1 Liquid Chromatography Conditions

Chromatographic separation was performed using a Waters XBridge Amide column (3.5 μm, 4.6 × 100 mm) maintained at 40 °C. The aqueous phase (A) consisted of 5 mM ammonium acetate in ultrapure water adjusted to pH 9.0 with aqueous ammonia and containing 5% acetonitrile, while the organic phase (B) was acetonitrile. The flow rate was 0.4 mL/min. The gradient elution program was as follows: 0–3.0 min, 85% B; 3.0–6.0 min, 85%–30% B; 6.0–15.0 min, 30%–2% B; 15.0–18.0 min, 2% B; 18.0–19.0 min, 2%–85% B; and 19.0–26.0 min, 85% B.

2.2 Mass Spectrometry Conditions

Analyses were performed using an AB SCIEX TripleTOF 5600 instrument equipped with an ESI source operated in negative ion mode. The TOF MS scan range was *m/z* 50–1000 and the product ion scan range was *m/z* 50–900. The source temperature was 500 °C, and the ion spray voltage floating was −4,500 V.

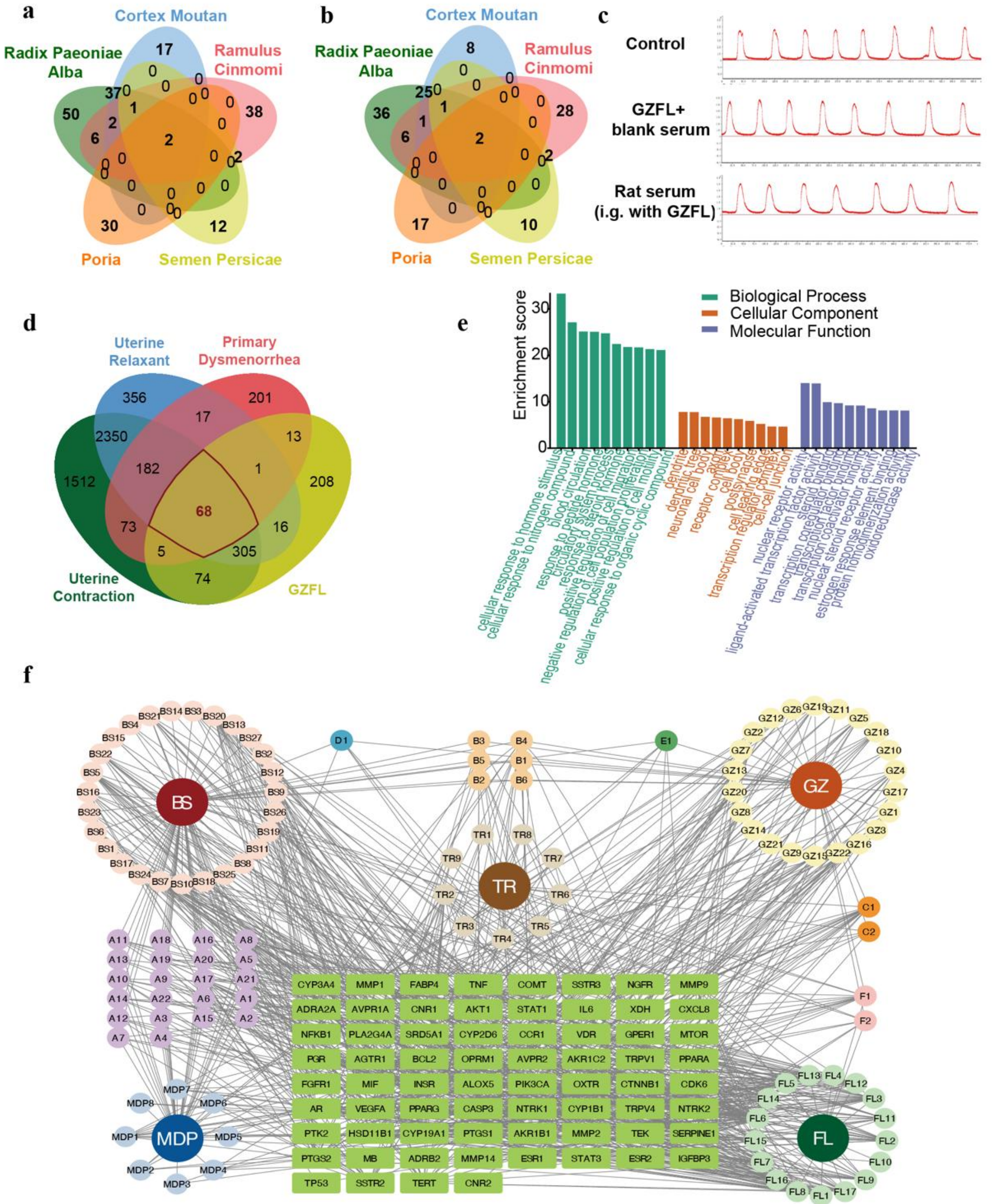

Figure S1. Supplementary characterization of the material basis, uterine contractile effects, and network pharmacological analysis of GZFL. (a) Distribution of the herbal origins of the 197 components characterized in GZFL extract. (b) Distribution of the herbal origins of the 136 serum-exposed components detected in drug-containing rat serum. (c) Representative ex vivo uterine contraction traces of the control, GZFL plus blank serum, and GZFL-containing rat serum groups. (d) Venn diagram illustrating the intersection of predicted GZFL pharmacological targets with targets associated with uterine contraction, uterine relaxation, and primary dysmenorrhea, yielding 68 candidate therapeutic targets. (e) Gene Ontology (GO) enrichment analysis of the 68 candidate therapeutic targets. (f) GZFL component–target interaction network involving the potential active components and 68 candidate therapeutic targets.

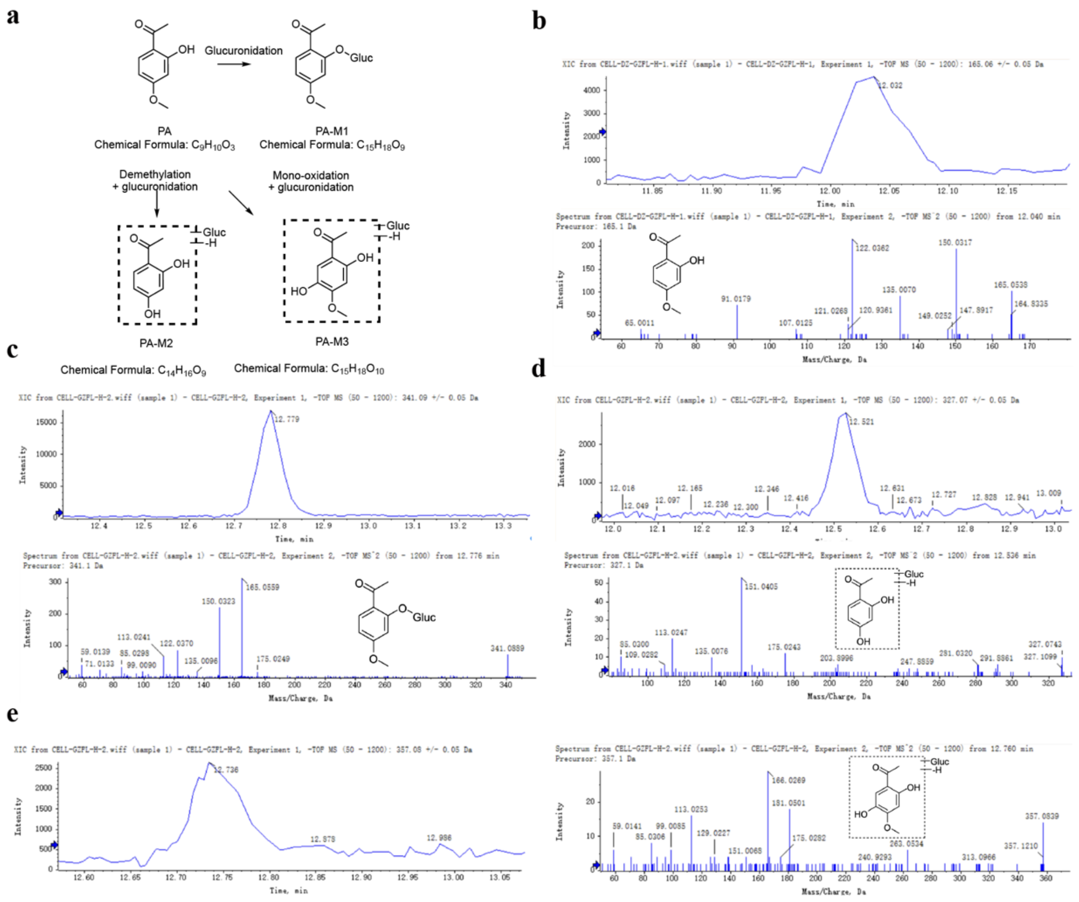

Figure S2. Proposed metabolic pathways and UPLC/Q-TOF-MS characterization of paeonol and its metabolites in primary human hepatocytes. (a) Proposed metabolic pathways of paeonol (PA) in primary human hepatocytes. Extracted ion chromatograms and corresponding product-ion spectra of (b) PA, (c) PA-M1, (d) PA-M2, and (e) PA-M3. PA-M1, PA-M2, and PA-M3 were tentatively assigned based on accurate-mass and MS/MS fragmentation data. Detailed mass spectrometric information is provided in Table 4.

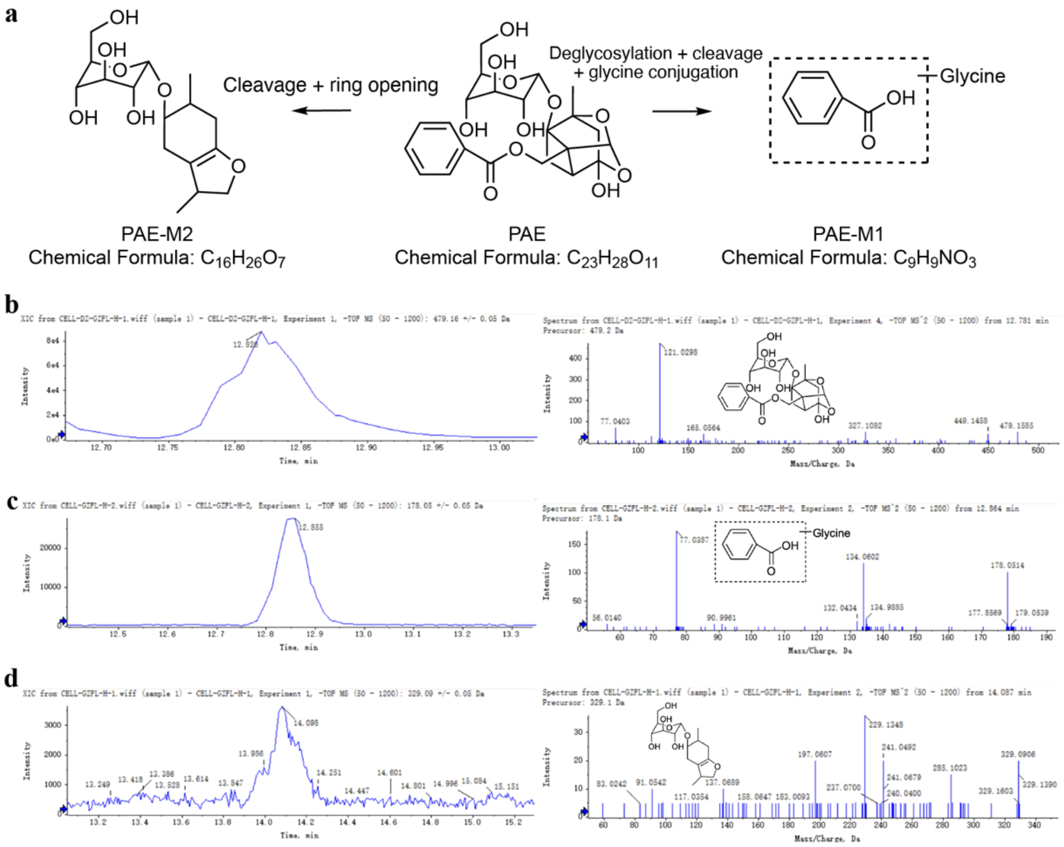

Figure S3. Proposed metabolic pathways and UPLC/Q-TOF-MS characterization of paeoniflorin and its metabolites in primary human hepatocytes. (a) Proposed metabolic pathways of paeoniflorin (PAE) in primary human hepatocytes. Extracted ion chromatograms and corresponding product-ion spectra of (b) PAE, (c) PAE-M1, and (d) PAE-M2. PAE-M1 and PAE-M2 were tentatively assigned based on accurate-mass and MS/MS fragmentation data. Detailed mass spectrometric information is provided in Table 4.

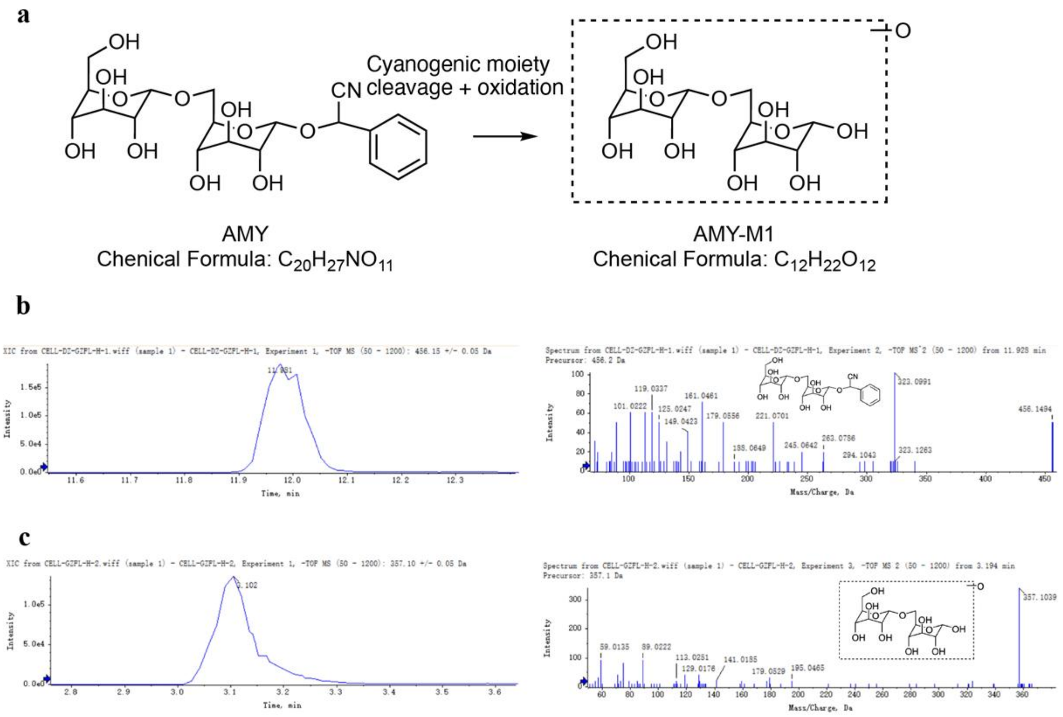

Figure S4. Proposed metabolic pathway and UPLC/Q-TOF-MS characterization of amygdalin and its metabolite in primary human hepatocytes. (a) Proposed metabolic pathway of amygdalin (AMY) in primary human hepatocytes. Extracted ion chromatograms and corresponding product-ion spectra of (b) AMY and (c) AMY-M1. AMY-M1 was tentatively assigned based on accurate-mass and MS/MS fragmentation data. Detailed mass spectrometric information is provided in Table 4.

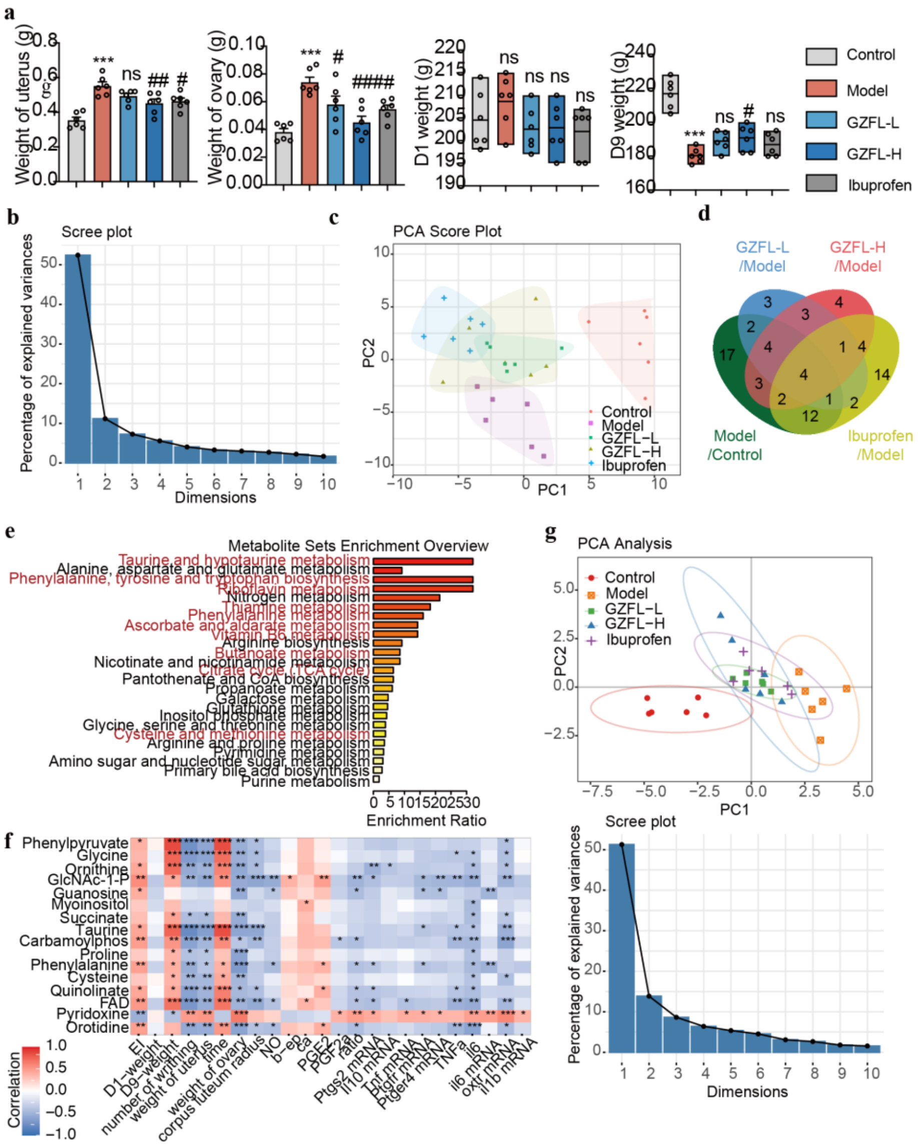

Figure S5. Supplementary pharmacodynamic and serum metabolomic analyses of GZFL in the acute dysmenorrhea rat model. (a) Uterine and ovarian weights and body weights on days 1 and 9 across experimental groups. (b) Scree plot of principal components derived from PCA of pharmacodynamic indicators. (c) PCA score plot of serum metabolomic profiles across experimental groups. (d) Venn diagram of differentially expressed endogenous metabolites across pairwise group comparisons. (e) Metabolite set enrichment analysis of differentially expressed endogenous metabolites. (f) Spearman correlation analysis between differential endogenous metabolites and pharmacodynamic efficacy indicators. (g) PCA score plot and scree plot based on the integrated differential endogenous metabolite profiles across experimental groups. Data are presented as mean ± SEM (n = 6). Compared with the control group: *P < 0.05, **P < 0.01, and ***P < 0.001; compared with the model group: #P < 0.05, ##P < 0.01, ###P < 0.001.

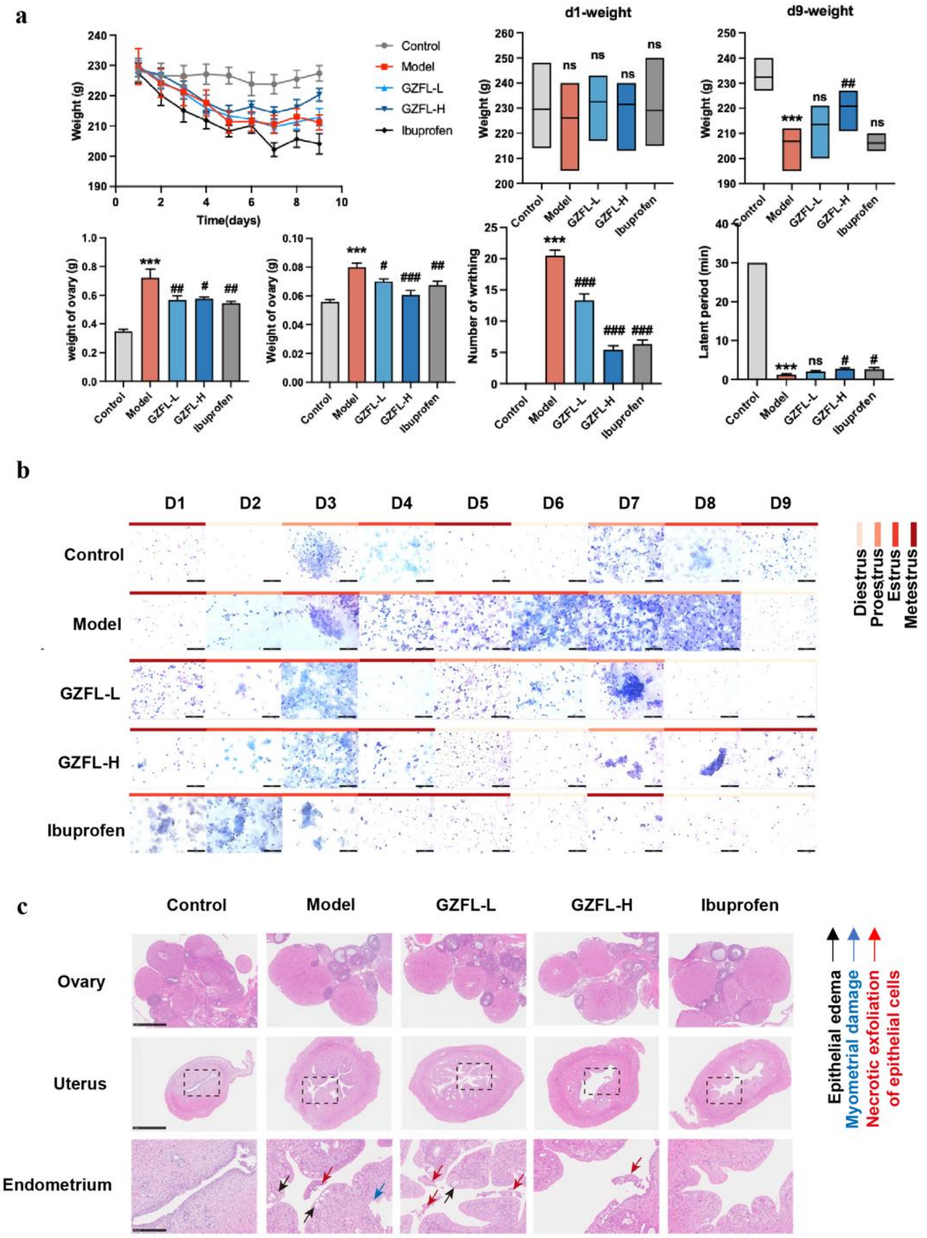

Figure S6. In vivo efficacy of GZFL against dysmenorrhea in rats with cold coagulation and blood stasis. (a) Body weight, uterine and ovarian weights, writhing times, and latency period of rats in each group after modeling. (b) Estrous cycle changes in rats in each group during modeling (scale bar = 100 μm). (c) Histopathological sections of the ovary, uterus, and endometrium in each group (ovary and uterus: scale bar = 400 μm; endometrium: scale bar = 200 μm). Data are presented as mean ± SEM (n = 6). Compared with the control group: *P < 0.05, **P < 0.01, and ***P < 0.001; compared with the model group: #P < 0.05, ##P < 0.01, ###P < 0.001.

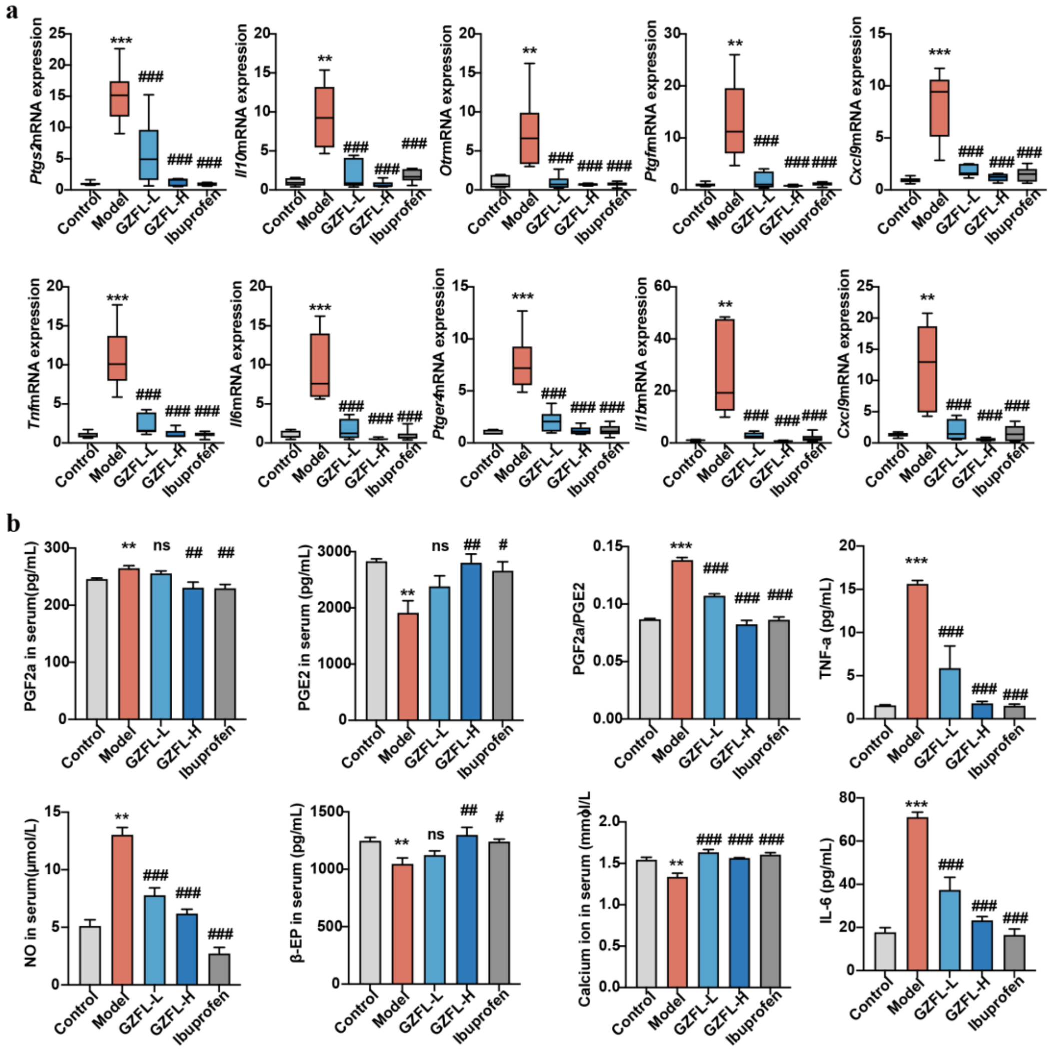

Figure S7. Effects of GZFL on inflammation-related gene expression and serum biochemical indicators in rats with cold coagulation and blood stasis dysmenorrhea. (a) mRNA expression of inflammation-related genes in the uterus of rats in each group. (b) Serum biochemical indicators in rats in each group. Data are presented as mean ± SEM (n = 6). Compared with the control group: *P < 0.05, **P < 0.01, and ***P < 0.001; compared with the model group: #P < 0.05, ##P < 0.01, ###P < 0.001.

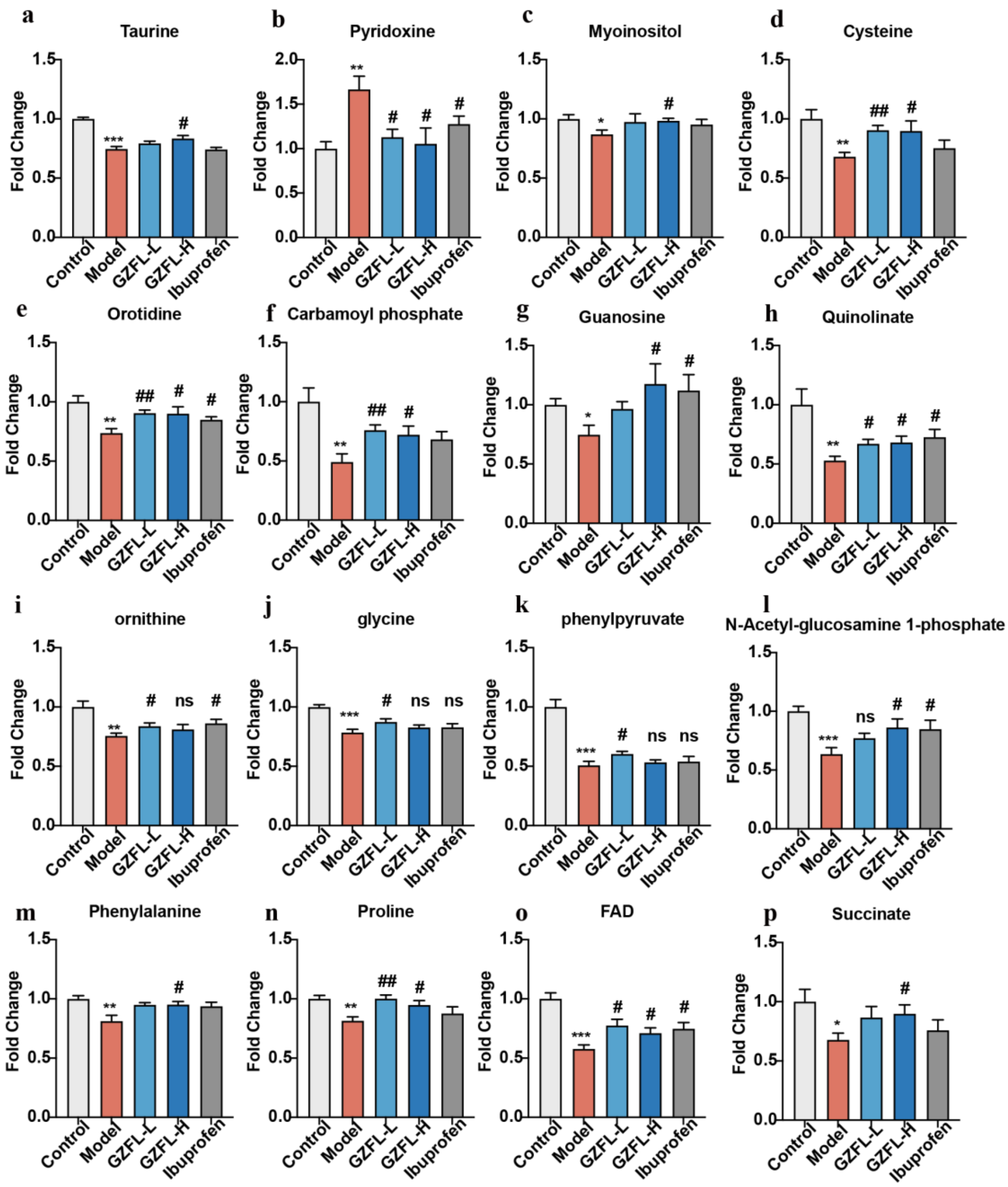

Figure S8. Changes in the relative levels of differential endogenous metabolites in serum following GZFL treatment. Relative levels of representative differential endogenous metabolites in the control, model, GZFL-L, GZFL-H, and ibuprofen groups. Data are presented as mean ± SEM (n = 6). Compared with the control group: *P < 0.05, **P < 0.01, and ***P < 0.001; compared with the model group: #P < 0.05, ##P < 0.01, ###P < 0.001.

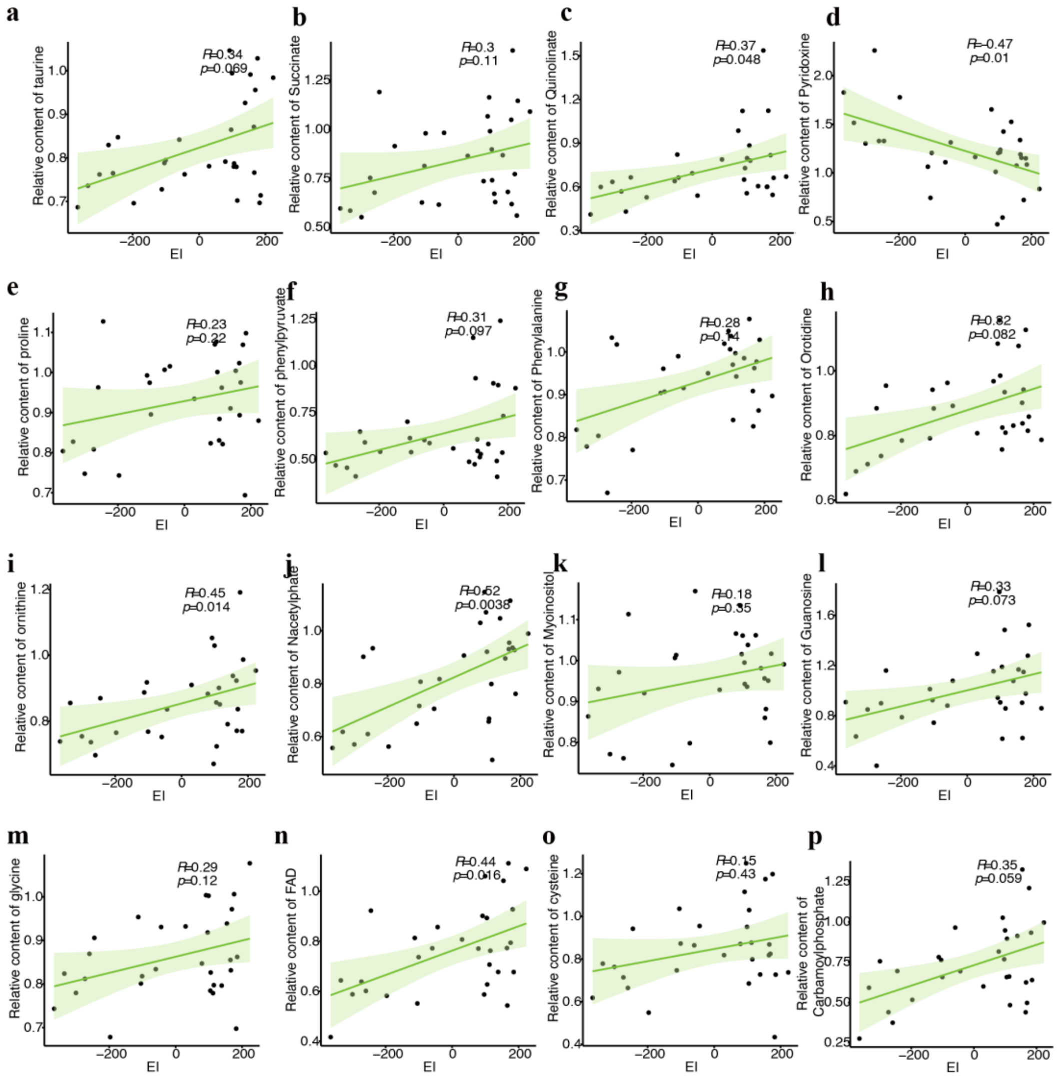

Figure S9. Spearman correlation analysis between differential endogenous metabolites and the integrated efficacy index (EI). Spearman correlation analysis between the relative levels of differential endogenous metabolites and EI. Correlation coefficients and corresponding P values are indicated in each panel.

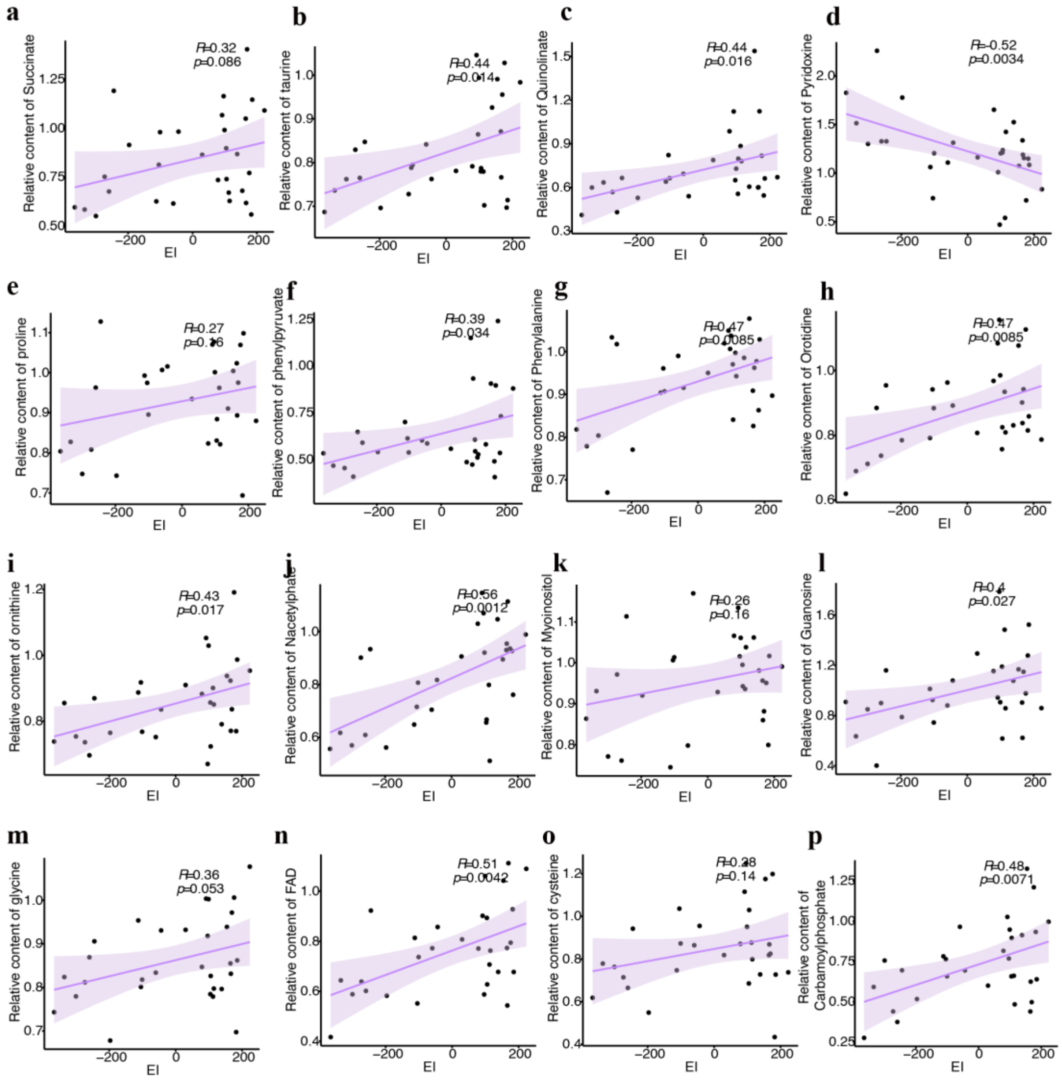

Figure S10. Pearson correlation analysis between differential endogenous metabolites and the integrated efficacy index (EI). Pearson correlation analysis between the relative levels of differential endogenous metabolites and EI. Correlation coefficients and corresponding P values are indicated in each panel.

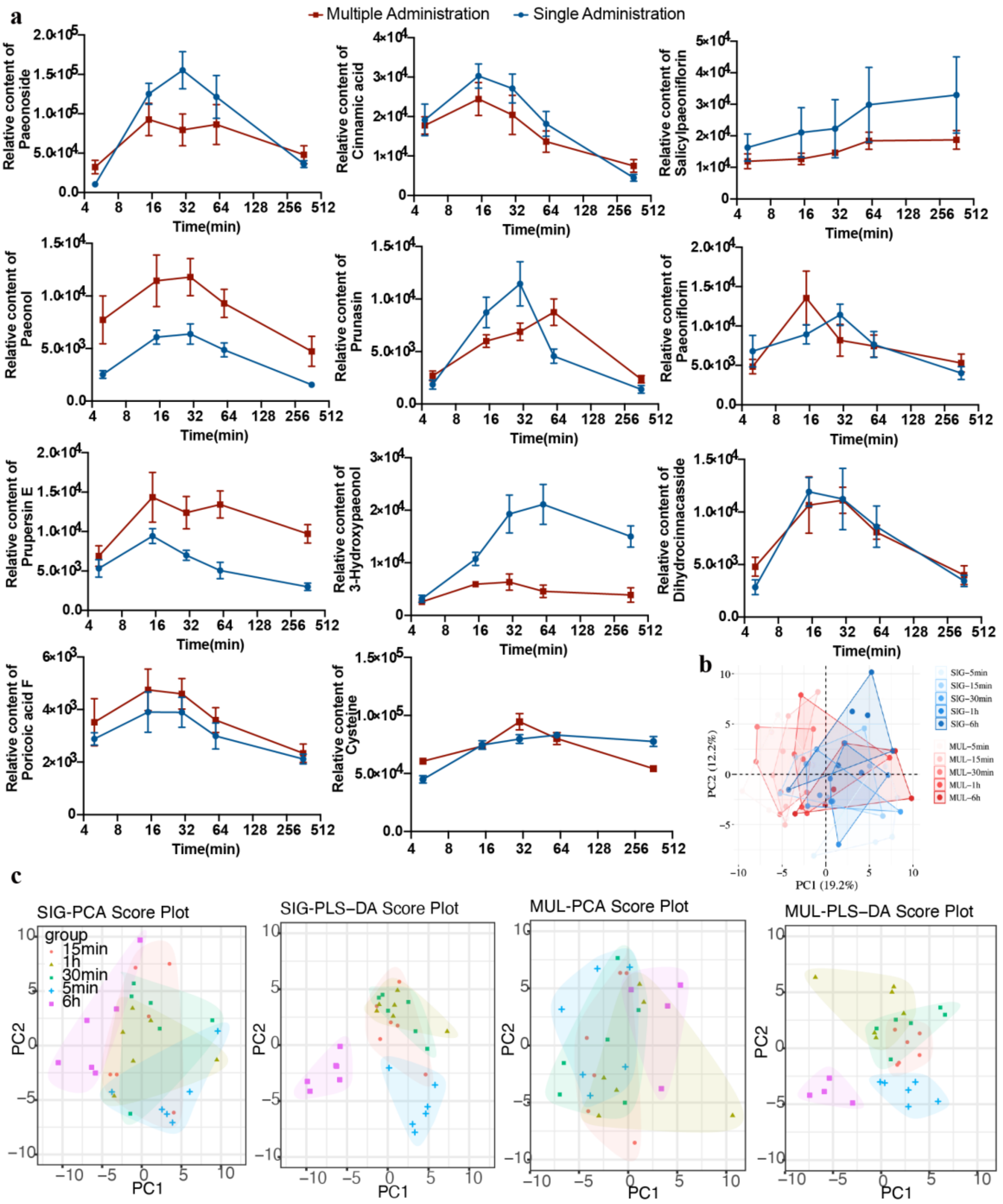

Figure S11. Changes in serum levels of potential active components and multivariate analysis of serum endogenous metabolites. (a) Relative concentration–time profiles of potential active components following single and multiple administrations of GZFL. (b) PCA score plot comparing serum endogenous metabolic profiles at different time points following single and multiple administrations of GZFL. (c) PCA and PLS-DA score plots of serum endogenous metabolites at different time points following single and multiple administrations of GZFL.

a

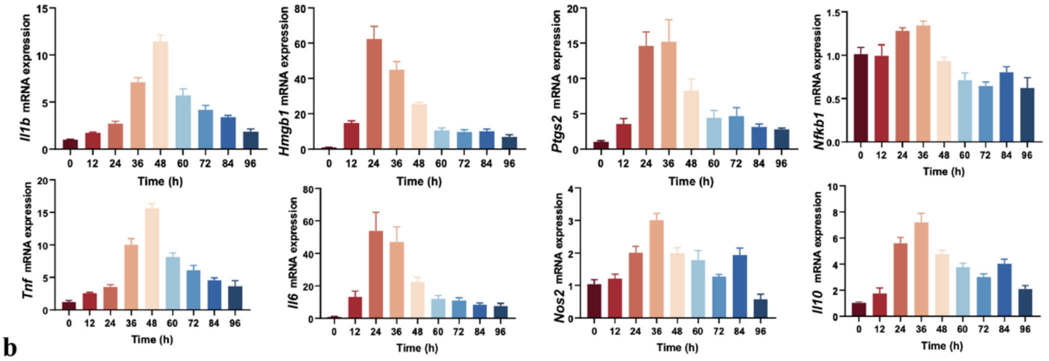

b

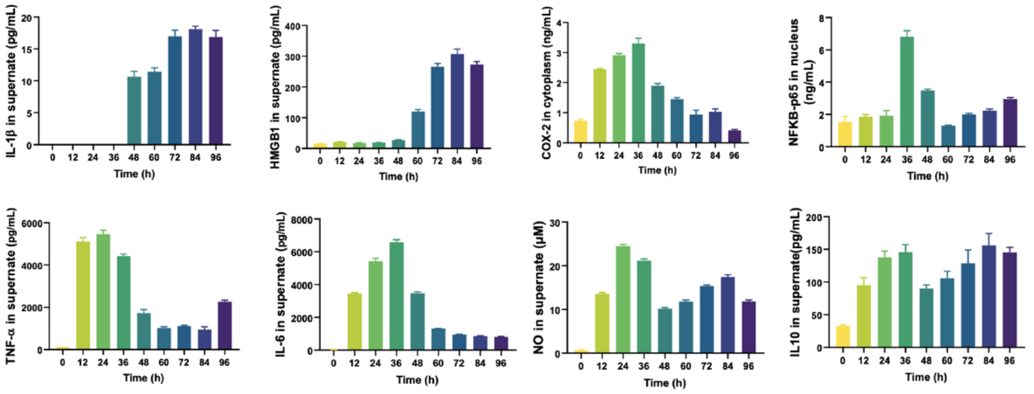

Figure S12. Time-course changes in inflammatory responses in LPS-stimulated RAW264.7 cells. (a) Time-course changes in the mRNA expression of representative inflammation-related factors in LPS-stimulated RAW264.7 cells. (b) Time-course changes in representative inflammatory mediators and proteins in LPS-stimulated RAW264.7 cells.

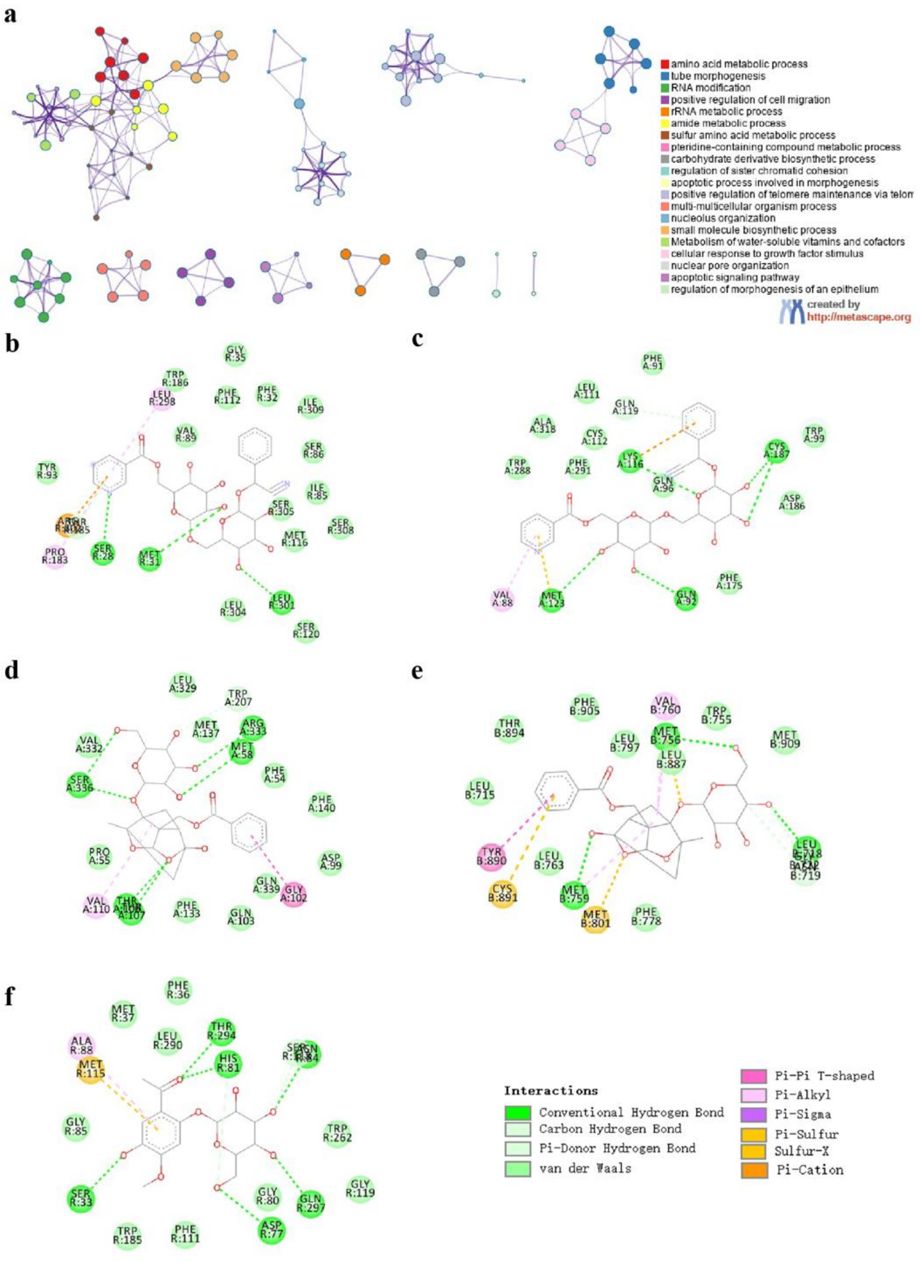

Figure S13. Metascape enrichment network and representative molecular docking interactions of GZFL-derived components with dysmenorrhea-associated targets. (a) Network visualization of enriched biological processes among the candidate therapeutic targets. Representative two-dimensional interaction diagrams of (b) Prupersin E with PTGER2, (c) Prupersin E with OXTR, (d) Paeoniflorin with PTGER3, (e) Paeoniflorin with PGR, and (f) PA-M11 with PTGFR. Dashed lines indicate predicted non-covalent interactions between the ligands and surrounding amino acid residues, with interaction types indicated in the legend.
